# Bacterial repetitive extragenic palindromic sequences are DNA targets for Insertion Sequence elements

**DOI:** 10.1186/1471-2164-7-62

**Published:** 2006-03-24

**Authors:** Raquel Tobes, Eduardo Pareja

**Affiliations:** 1 Bioinformatics Unit, Era7 Information Technologies SL, BIC Granada CEEI, Parque Tecnológico de Ciencias de la Salud – Armilla Granada 18100, Spain

## Abstract

**Background:**

Mobile elements are involved in genomic rearrangements and virulence acquisition, and hence, are important elements in bacterial genome evolution. The insertion of some specific Insertion Sequences had been associated with repetitive extragenic palindromic (REP) elements. Considering that there are a sufficient number of available genomes with described REPs, and exploiting the advantage of the traceability of transposition events in genomes, we decided to exhaustively analyze the relationship between REP sequences and mobile elements.

**Results:**

This global multigenome study highlights the importance of repetitive extragenic palindromic elements as target sequences for transposases. The study is based on the analysis of the DNA regions surrounding the 981 instances of Insertion Sequence elements with respect to the positioning of REP sequences in the 19 available annotated microbial genomes corresponding to species of bacteria with reported REP sequences. This analysis has allowed the detection of the specific insertion into REP sequences for ISPsy8 in *Pseudomonas syringae *DC3000, ISPa11 in *P. aeruginosa *PA01, ISPpu9 and ISPpu10 in *P. putida KT2440*, and ISRm22 and ISRm19 in *Sinorhizobium meliloti *1021 genome. Preference for insertion in extragenic spaces with REP sequences has also been detected for ISPsy7 in *P. syringae *DC3000, ISRm5 in *S. meliloti *and ISNm1106 in *Neisseria meningitidis *MC58 and Z2491 genomes. Probably, the association with REP elements that we have detected analyzing genomes is only the tip of the iceberg, and this association could be even more frequent in natural isolates.

**Conclusion:**

Our findings characterize REP elements as hot spots for transposition and reinforce the relationship between REP sequences and genomic plasticity mediated by mobile elements. In addition, this study defines a subset of REP-recognizer transposases with high target selectivity that can be useful in the development of new tools for genome manipulation.

## Background

The term "REP sequences" encompasses repetitive and palindromic sequences with a length between 21 and 65 bases [1] detected in the extragenic space of some bacterial genomes. The function of REP elements is not completely determined but there are important processes in which REP sequences are involved. It was proposed that REP sequences play a role as transcriptional attenuators [2] although it was later stated that REP sequences are not specific terminators [3]. Based on their role as mRNA stabilizers [4], it has also been suggested that REP elements are involved in the fine tuning of gene-expression [5]. REP sequences are binding sites for DNA polymerase I [6], for DNA gyrase [7], and for Integration Host Factor (IHF) [8], all of which play a key role in bacterial DNA physiology. There are also some cases in which REP sequences appear as targets for transposition and recombination events. In this sense, it has been shown that IS1397 and IS621 insert specifically within REP sequences of *Escherichia coli *and that ISKpn1 insert into REP sequences of *Klebsiella pneumoniae *[9-12]. REP sequences also appear at the recombination junctions of lambda bio phages [13] and amplification of plasmid F_128 is initiated by REP-REP recombination [14].

REP elements and binding sites for global regulators share common features such as size, palindromic structure, and multiple locations in the extragenic space of the genomes. The DNA binding sites for global regulators are placed in multiple sites along the genome, far from their corresponding genes. This fact makes it difficult to detect all the binding sites corresponding to a global regulator without a specific definition of their binding sequence on the DNA. However, in the case of transposases, each DNA binding-site is placed around the insertion point of the mobile element. Hence, each transposition event stays registered on the genome, allowing the tracing of the last DNA sites bound by each transposase. Considering that there were a sufficient number of available genomes corresponding to organisms with a described presence of REP, and exploiting the advantage of the traceability of transposition events in genomes, we decided to analyze the relationship between REP sequences and mobile elements. We have carried out an exhaustive study of all the insertion sites of mobile elements in the genomes with REP elements. This analysis has allowed us to detect that REP sequences are specific targets of insertion for IS elements in the genomes of *Pseudomonas syringae pv. tomato *DC3000, *Pseudomonas aeruginosa *PA01, *Pseudomonas putida *KT2440, *Sinorhizobium meliloti *1021, and a probable association in *Neisseria meningitidis *MC58 and *Neisseria meningitidis *Z2491.

## Results

Analyzing the results obtained in our study of the association between REP sequences and mobile elements, we have distinguished two types of associations: (i) type 1 association, in which the percentage of association is 100% and each IS copy is inserted in the same position of a REP sequence, making it possible to define the DNA target consensus sequence (Tables 1 and 2 and Figure 2) and (ii) type 2 association, in which the IS elements are near to, or adjacent to REP sequences, but fragments of broken REP sequences just flanking IS elements are not detected (Tables 1 and 2).

**Figure 1:**
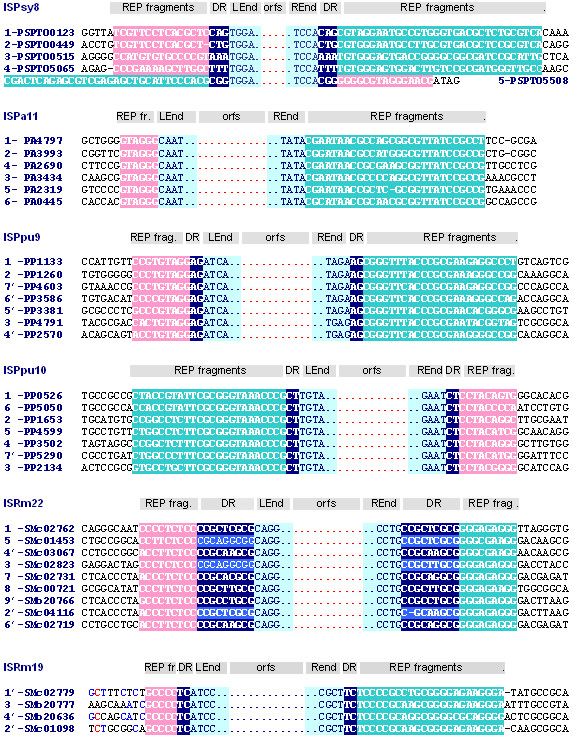
**Multiple alignment of the flanking DNA sequences of the Insertion Sequence elements that present a type 1 association with REP elements**. The fragments of broken REP sequences are indicated in pink and aquamarine. The direct repeats (DR) appear shadowed in blue at both extremes of the sequences of the Insertion Sequence elements. The arrangement of the different parts of the IS element is indicated at the top grey bar as "LEnd" for left end, "orf" for the transposase orfs and "REnd" for the right end.

**Figure 2:**
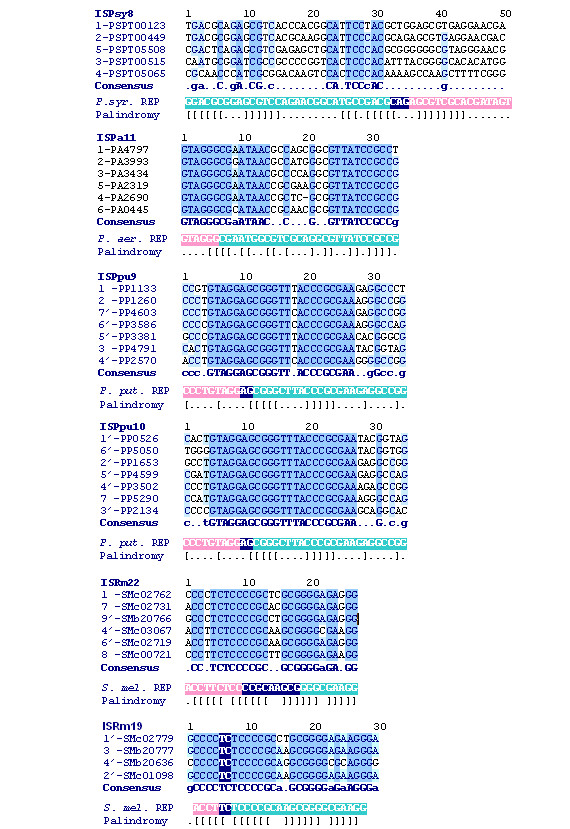
**Reconstructed REP sequences at the insertion sites of ISs with type 1 association with REP sequences**. The reconstructed REP sequences displayed in the figure have generally gone unnoticed because it is needed to join the two fragments that are intervened by the IS element to reconstruct the complete REP sequence. The figure shows the multiple alignment of the reconstructed REP sequences with the conserved bases shadowed in blue. The canonical REP sequence is at the bottom of each alignment. One of the REP sequence fragments is in pink, the direct repeat generated in the transposition event is shadowed in blue, and the other REP sequence fragment is in aquamarine. The palindromy of canonical REP sequences is indicated in bracket notation.

We have detected a type 1 association for ISPsy8 in *P. syringae *DC3000, for ISPa11 in *P. aeruginosa *PA01, for ISPpu9 and ISPpu10 in *P. putida *KT2440, and for ISRm22 and ISRm19 in *S. meliloti *1021 genome. Figure 1 shows IS elements flanked by the two fragments of the broken REP sequences, and the alignments of the reconstructed REP sequences corresponding to the insertion sites are shown in Figure 2. In addition, the alignments of the complete sequences of each IS element, including their flanking regions, are in the additional material [see Additional file 1, file 2, file 3, file 4, file 5 and file 6]. Remarkably, in all cases of type 1 association, 100% of the copies of each IS are associated to REP (Tables 1 and 2), proving a high selectivity for their REP sequence target. The results of this sequence analysis allow us to affirm that REP elements are target sequences for transposases.

There are five ISPsy8 elements in the *P. syringae *DC3000 genome and in all cases, their insertions were into a REP sequence. ISPsy8 always broke the REP element at exactly the same point of the sequence, generating a direct repeat of three bases (Figure 1) [see Additional file 1]. A conserved arrangement that consists of a fragment of the REP sequence, a direct repeat of three bases, the left end of the ISPsy8, the transposase OrfA, the transposase OrfB, the right end, the other direct repeat, and the remaining fragment of REP sequence is maintained in all ISPsy8 insertion areas (Figure 1) [see Additional file 1]. In four cases, the broken REP elements are in the minus strand, and in one case, the broken REP element is located in the plus strand (Figure 1). However, in all cases, the transposase ORFs are in the plus DNA strand [see Additional file 1]. The point of insertion within the REP element is exactly between the bases occupying positions 32 and 33 of the REP sequence. All these REP elements share a consensus sequence (Figure 2) adjacent to the ISPsy8 insertion point. A direct repeat of three base pairs, corresponding to positions 33, 34 and 35 of each broken REP sequence, is generated and appears at both extremes of the IS element (Figure 1) [see Additional file 1]. Palindromy can probably induce REP sequences to adopt hairpin secondary structures. Strikingly, the ISPsy8 insertion site is located just at the symmetry axis of one of the two probable hairpin structures predicted for REP sequence of *P. syringae *[5] (Figure 2) [see Additional file 1]. The allocation of ISPsy8 into clusters of REP elements was determinant for the detection of REP elements broken at the ISPsy8 insertion point. In four cases, ISPsy8 was inserted into a cluster of REP sequences and in one case, it was inserted into an isolated REP element [see Additional file 7]. When we joined the two fragments located at both sides of ISPsy8, the REP sequence appeared perfectly reconstructed (Figure 2) [see Additional file 1]. In the cases where the broken REP sequence formed part of a cluster, its reconstructed sequence was very similar to the REP sequences that shared the same orientation in the cluster [see Additional file 8]. This was as expected considering that each cluster used to have two differentiated types of REP sequences [5]. This seems to support the idea that REP sequence fragments flanking ISs do not form part of the IS inverted repeats, but they are the fragments of the target sequences broken by IS transposition.

In *P. aeruginosa *PA01, there are six copies of ISPa11, and we found the same fragments of REP sequence flanking each ISPa11 copy (Figure 1) [see Additional file 2]. In this case, we defined the insertion sites that lacked the usual direct repeats (Figure 1). The arrangement detected for ISPsy8 was also conserved for ISPa11(Figure 1). In this case, all broken REP sequences were in the same DNA strand [see Additional file 2]. For ISPa11, the point of insertion within the REP element is between the sixth and seventh bases of the REP sequence. The shared consensus sequence obtained reconstructing and aligning the six broken REP sequences is displayed in Figure 2.

In *P. putida *KT2440, all copies of ISPpu9 and ISPpu10 were inserted into *P. putida *REP sequences (Figure 1) [see Additional file 3 and file 4]. Both ISs generate direct repeats of two base pairs at the insertion point (Figure 1). Our data are in agreement with insertion site data previously reported [15]. It is important to note that, although ISPpu9 and ISPpu10 sequences are not highly similar, both are inserted exactly between the ninth and tenth bases of the REP sequence. In addition, the insertion site consensus sequence is extraordinarily conserved, and the insertion site sequence is practically identical for all copies in both ISs (Figure 2).

In *S. meliloti *1021, all copies of ISRm22 were inserted into *S. meliloti *REP elements (Figure 1) [see Additional file 5]. The analysis of ISRm22 flanking regions allowed us to characterize the ISRm22 insertion sites and to describe direct repeats of six base pairs generated at the insertion points. There are 9 copies of ISRm22 in the *S. meliloti *1021 genome but, curiously, only six copies have perfectly conserved direct repeats at both extremes of the IS. The three direct repeats marked with light blue background in Figure 1 are not perfect direct repeats. From observing their sequences, it could be the result of recombination events between the copies. Homologous inter- or intramolecular recombination between two IS elements, each with a different direct repeat, would result in a hybrid element carrying one direct repeat of each parent, and is the case for these three ISRm22 instances [16]. Formation of adjacent deletions resulting from duplicative intramolecular transposition could also result in a single copy of the direct repeat located on each of the reciprocal deletion products [16]. We have reconstructed the REP sequences corresponding to the six ISRm22 copies with identical direct repeats, thus obtaining a clearly defined consensus sequence at the point of insertion. This consensus is highly palindromic and the insertion point is located just at the palindromy axis (Figure 2). The other case of type 1 association detected in *S. meliloti *1021 genome was ISRm19. The four instances of ISRm19 were also inserted into REP sequences (Figures 1 and 2) [see Additional file 6]

ISPsy7 in *P. syringae *DC3000, ISRm5 in *S. meliloti*, and ISNm1106 in *N. meningitidis *MC58 and in *N. meningitidis *Z2491 genomes present a type 2 association with REP sequences (Tables 1 and 2). The alignments corresponding to their sequences and their flanking regions are in the additional material [see Additional file 9, file 10, file 11 and file 12]. The set of proteins with COG3547 that include proteins annotated as transposases and as PivNM also presents a type 2 association in *N. meningitidis *MC58 (Tables 1 and 2).

## Discussion

We have detected associations between REP sequences and IS elements in 6 out of 19 analyzed genomes. In the set of genomes without association, there are cases with absent IS elements along the genome (*Rickettsia conorii*), and cases with scarce presence (*A. tumefaciens*). In addition, we have adopted a strict criterion to select the IS elements associated with REP sequences. In several cases, the limited number of IS copies do not allow the determination of IS elements as a REP recognizer (Table 1). One of these cases could be the case of IS1397 in *E. coli*. It is experimentally proved that IS1397 [11,12,17] can insert into REP elements but, within the available genomes of Enterobacteriaceae, there are only two instances of IS1397, which are in *E. coli *CFT073 (Table 1). In one of these instances, IS1397 is clearly inserted into an *E. coli *REP element [see Additional file 13], but does not fulfill the association criteria of our study. Probably, the association with REP elements detected in analyzing genomes was only the tip of the iceberg, and it could be that many IS elements chose REP sequences as their targets in natural isolates.

While many IS elements display little obvious target site selectivity, some IS elements display considerable selectivity [18]. We have detected a set of elements displaying high target selectivity by REP sequences (elements with type 1 association, Table 2). There is experimental evidence for IS1397 and ISKpn1, suggesting that transposases themselves appear to be responsible for target specificity [12]. The results of our study show that REP-targeting is not restricted to only one IS family, but it extends to five different IS families: IS3, IS110, IS4, IS256 and IS5. This is not surprising since the features of the DNA-target and of the transposase domain responsible for target choice, are not included in the criteria to define IS families [16].

There are two families that include elements with experimental evidence of target selectivity by REP sequences. One family is the IS3 family which includes IS1397 and ISKpn1 [11,12,17]. The other one is the IS110 family, to which belongs IS621 [10]. We have detected additional IS elements belonging to these two families that specifically transpose into REP elements. The element representing the IS3 family that we have detected with a strong type 1 association with *P. syringae *REP sequences is ISPsy8 (Tables 1 and 2). Members of the IS3 family are similar in many aspects, and form an extremely coherent and highly related family. Usually, the transposase is encoded by two ORFs that are sometimes overlapping. The OrfB products, similar to retroviral integrases [19,20], carry a DD(35)E motif and are responsible for catalytic activity. The target recognition capability is usually located in the OrfA protein, which in various members of the family exhibits a relatively strong helix-turn-helix motif that could provide sequence-specific binding to DNA [21,22]. Many members also carry a putative leucine zipper located at the end of OrfA that could be involved in multimerization [23]. The N-terminal domain of the OrfA protein of ISPsy8 is positive for the Pfam Hidden Markov Model profile PF01527, named Transposase_8 (Table 2). The region identified by this profile includes a helix-turn-helix motif at the N terminus followed by a leucine zipper motif that is also present in other IS3 family elements. Probably, this HTH motif is involved in DNA target choice. There are experiments proving that IS30 needs an H-HTH motif [24], similar to the H-HTH motif involved in the DNA binding of the response regulator FixJ, in order to bind specific DNA target sequences [24]. This data suggests that some transposases could recognize palindromic REP sequences in a similar way that some transcriptional regulators recognize their palindromic binding sites.

The IS110 family is the other family with one element, the IS621, with an experimentally proved REP sequence target [10]. In our genomic analysis results, this family is the family most represented in the set of IS elements associated with REP sequences (Table 2). IS110 is a very special family of IS elements that has characteristics very different from the other families. The majority of their members have not inverted repeats flanking the transposase gene and little overall similarity can be detected between the ends. The mechanism of transposition of these elements is not well determined. However, the target sequences of the members IS117 from *Streptomyces coelicolor *and IS900 from *Mycobacterium paratuberculosis *exhibit similarities to the circle junction, suggesting an insertion mechanism by site specific recombination [16,25,26]. The site-specific invertase Piv from *Moraxella lacunata *also belongs to the IS110 family. This protein is included in the IS110 family, because it exhibits amino acid homology with the transposases of this family. The tertiary structure of amino-terminal domain of Piv invertase has been modelled [27], based on crystal structures of catalytic domains of HIV-1 integrase [28], avian sarcoma virus integrase (ASV) [29], and Tn5 transposase-related inhibitor protein [30], and the predicted structure matched with mutagenesis studies [27]. These results led Tobiasson and colleagues to propose that Piv invertase and the IS110 transposases could mediate DNA recombination by a common mechanism involving a catalytic DED or DDD motif [27]. Our study adds data that relates the IS110 family with site specific recombination processes. ISPa11, ISPpu9 and ISPpu10 exhibit a high selectivity in their target choice and could share mechanisms of target recognition and/or catalytic activity with some site-specific recombinases and viral integrases.

Using pairwise whole genome alignments, it is possible to segment bacterial genomes into a common conserved backbone and strain-specific sequences called loops [31]. These strain-specific loops include mobile elements, genes adapted to specific ecological environments, genes involved in pathogenicity, and other genes acquired by horizontal gene transfer. Strikingly, whole genome comparative analysis in Escherichia coli strains showed that strain-specific loops are associated with BIMEs (composed by different types of *E. coli *REP elements) [31]. In parallel, the mapping of the IS elements in different *E. coli *strains revealed that ISs are associated with deletion of genome fragments and incorporation of horizontally acquired genes [32]. In addition, some phenotypic features of *E. coli *are explained by the inactivation of genes by IS elements. This is the case for the absence of expression of the OmpC porin with the correspondingly elevated expression of the OmpF porin reported for *E. coli *B [32]. Thus, REP elements and IS elements are related with similar genome evolution events. Our detection of REP elements as frequent targets for transposases could explain the involvement of both in common genome plasticity phenomena. All these facts suggest that REP-recognizer transposases could be contributing to the repertoire of bacterial adaptive mechanisms.

The IS4 family had not been previously related to REP sequence target selectivity, but our genome analysis has detected that ISRm22, a member of this family, has its nine copies inserted into REP elements along the *S. meliloti *1021 genome (Figure 1 and 2 and Table 2). There is data about the Tn5 transposon that helps to understand this IS4 family. The Tn5 transposon is comprised of a cluster of antibiotic resistance genes bordered by two IS50 Insertion Sequences. IS50 belongs to the IS4 family and a truncated version of the IS50 transposase that contains the catalytic active site, termedTn5 transposase-related inhibitor protein, has been crystallized [30]. The structure of its catalytic domain is probably similar to the Piv invertase member of the IS110 family of transposases (See above), connecting both families with detected REP-recognizer members. One of the characteristics frequently found for T*n5 *transposition target sites is the palindromic structure of the insertion site, and also, there is a frequent occurrence of GC pairs at each end of the Direct Repeats [33,34]. The insertion sites that we have detected for ISRm22 fulfill both requirements (Figure 2). Another proposed characteristic of T*n5 *transposition is the preferable integration in actively transcribing or highly super-coiled DNA regions [33]. In this sense, REP sequences are frequently located in regions between convergent genes. These DNA fragments are especially prone to be highly supercoiled since simultaneous transcription of both convergent genes can generate increased positive supercoiling at the end of the genes [3]. Through testing the frequency of Tn5 insertion into specifically designed synthetic target sequences, it has been found that IS50 recognizes a preferred 9-bp sequence as its target. Moreover, sequences resembling this consensus target function optimally when embedded in a cluster of overlapping similar sequences [33]. In accordance with these Tn5 data, we have found that the majority of ISRm22 copies are inserted into a cluster of REP sequences.

In the type 1 association cases (ISPa11, ISPpu9, ISPpu10, ISRm22 and ISRm19) the conserved sequence encompasses almost the complete REP sequence (Figure 2). All consensus sequences share a high percentage of GCs, a greater conservation in GCs than in ATs, a palindromic structure, and a similar length (with the exception of ISPsy8 which displays a shorter consensus). In spite of the differences in their corresponding transposase sequences, ISPpu9 and ISPpu10 show the same point of insertion within the consensus sequence. REP-recognizer ISs could share some features in their target recognition domains. The determination of the transposases belonging to this subset could provide new clues to search for a common mechanism of recognizing the DNA target.

Target selectivity differs significantly between different ISs. While some ISs display high target specificity, other elements exhibit regional preferences that could reflect more global parameters such as local DNA structure [16]. Thus, regional specificity has been related with GC or AT abundance, degree of supercoiling, DNA bending, replication related factors, and transcription related factors [16]. Transposition activity is frequently modulated by various host factors. The list of such factors includes the histone-like protein IHF, which has been experimentally proved to bind REP sequences. Another two REP-binder proteins, DNA polymerase I [35,36] and DNA gyrase [37-39] have also been implicated in transposition activity. Clusters of REP sequences could provide an appropriate context to recruit all the elements playing a role in transposition. The detected type 2 associations could reflect a favourable context for transposition provided by REP sequence clusters in combination with a minor stringency for the DNA target.

REP elements have also been related to recombination events. Thus, REP sequences have been found at the recombination junctions of lambda bio transducing phages [13] and it has been experimentally detected that amplification of plasmid F_128 is initiated by REP-REP recombination [14]. REP elements are DNA points especially suitable for undergoing transposition or recombination events, because they are frequently placed at extragenic spaces limited by convergent genes [5]. Their extragenic location would warrant that transposition did not disrupt genes. Their preference for spaces between convergent genes would make it probable that transcriptional regulatory signals remained unaltered, since the end of two genes is not a site for recruitment of transcriptional regulators. Moreover, taking into account that bacteriophage Mu is excluded from insertion in regions of DNA to which regulatory proteins are bound [40], spaces between convergent genes would have the additional advantage of being sites always free of bound regulators. Furthermore, spaces limited by convergent genes usually are spaces between two independent transcriptional units. Hence, REP sequences could be used as tags, generally positioned at the end of the genes, indicating genome points especially advantageous for transposition. Thus, the characterization of some REP elements as hot spots for recombination and transposition suggests that, probably, REP elements are key elements in adaptive bacterial evolution. REP sequences provide genome points that warrant secure recombination and transposition without severe detrimental effects. Moreover, REP sequences are genome elements that can vary in position and number supplying additional variability to this set of selectable points of insertion. Taking this into consideration, it is probable that comparative genomics studies between phylogenetically close strains could be more revealing. Transposition plays a crucial role in horizontal gene transfer in bacteria, including the spread of antibiotic resistance [41-43]. In addition, some virulence genes are regulated by transposition [44] and it is proven that some insertions, deletions, inversions and chromosome fusions are caused by transposition [45,46]. REP sequences could be playing a role in these important mechanisms.

## Conclusion

This global study highlights the importance of REP sequences as DNA targets for the transposition of mobile elements and supplies new data that throws light on REP sequence role, transposase DNA target choice, and genomic plasticity. In addition, the targets for transposition characterized in this study could open the door to new tools for genome manipulation.

## Methods

There are 19 completely annotated microbial genomes with REPs that correspond to 13 species of bacteria (Table 1) [see Additional file 14], and in all of them, we have investigated all the points of insertion of mobile elements. We have used the genome annotations available at the NCBI website [47]

Multiple alignments of the sequences have been obtained using the program Multalign [48,49] and CLUSTAL W [50,51]. In some cases, the extremes of the ISs were not clearly defined and we have defined them by comparing and aligning the sequences of all copies. Manual correction of some alignments and alignment of fragments was necessary in cases of partial sequences and copies with internal insertions.

We have defined the boundaries of each IS copy based on data obtained from the annotations of the genomes and from ISfinder database [52], but in many cases, there were not available data and we have studied the IS ends by managing multiple alignment data and the meticulous analysis of the sequences.

To facilitate the detection of association between the genome positions corresponding to REP elements and IS genome positions, we have used C++ programming.

To analyze the domain structure of ISs we have used Pfam database [53,54].

After analyzing the presence of REP sequences in the extragenic regions flanking the 981 instances of mobile elements present in the 19 analyzed genomes, we considered that there was association between REP sequences and mobile elements when the number of copies was equal or greater than four, and when the percentage of association was greater than 45% (Table 1).

## Authors' contributions

RT participated in the conception and design of the study, in the acquisition, analysis and interpretation of data, carried out the bioinformatics tasks and wrote the draft of the manuscript.

EP participated in the conception and design of the study, in the analysis of the data, and in the critical review of the manuscript.

## Supplementary Material

Additional file 1 **Alignment of DNA sequences from all copies of ISPsy8 in *Pseudomonas syringae *DC3000 and their flanking regions**. In the case named IS8-5 the broken REP element is in the plus strand and 32 bases of the REP element are upstream of the IS element while the remaining 20 bases are downstream. Symmetrically, in the four cases (IS8-1, IS8-2, IS8-3 and IS8-4) in which the REP element is placed in the minus strand, the 20 last bases of the REP sequence are in the minus strand upstream ISPsy8 and the first 31 bases of REP sequence are downstream. Click here for file

Additional File 2 Alignment of DNA sequences from all copies of ISPa11 in *Pseudomonas aeruginosa *and their flanking regions. Click here for file

Additional File 3 Alignment of DNA sequences from all copies of ISPpu9 in *Pseudomonas putida *KT2440 and their flanking regions. Click here for file

Additional File 4 Alignment of DNA sequences from all copies of ISPpu10 in *Pseudomonas putida *KT2440 and their flanking regions. Click here for file

Additional File 5 Alignment of DNA sequences from all copies of ISRm22 in *Sinorhizobium meliloti *and their flanking regions. Click here for file

Additional File 6 Alignment of DNA sequences from all copies of ISRm19 in *Sinorhizobium meliloti *and their flanking regions. Click here for file

Additional File 7 Positions of all ISPsy8 copies with adjacent genes and REP sequences. Click here for file

Additional File 8 Alignment of each reconstructed REP sequence with REP sequences with its same orientation and cluster. Click here for file

Additional File 9 Alignment of DNA sequences from all copies of ISPsy7 in *Pseudomonas syringae *DC3000 and their flanking regions. Click here for file

Additional File 10 Alignment of DNA sequences from all copies of ISRm5 in *Sinorhizobium meliloti *and their flanking regions. Click here for file

Additional File 11 Alignment of DNA sequences from all copies of ISNm1106 in *Neisseria meningitidis *MC58 with type 2 association with REP sequences and their flanking regions. Click here for file

Additional File 12 Copies of IS1106 in *Neisseria meningitidis *Z2491with type 2 association with REP sequences and their flanking regions. Click here for file

Additional File 13 IS1397 inserted into an *E. coli *REP element in *E. coli *CFT073 genome Click here for file

Additional File 14 Analyzed genomes Click here for file

Additional File 15 Canonical REP sequences corresponding to each analyzed species Click here for file

## Figures and Tables

**Table 1:** Analysis of the association of Insertion Sequence elements with REP sequences. Table 1 shows the results of the analysis of the presence of REP sequences in the extragenic spaces flanking mobile elements. The fifth column shows the association ratio between the number of mobile elements containing REP sequences in the flanking extragenic spaces and the total number of mobile elements in the analyzed genome. The canonical REP sequence for each analyzed species is in the additional material [see Additional file 15]

Genomes	GenBank Accession	IS copies	Mobile elements	Assoc. ratio	Assoc. %	REPs number
*Pseudomonas syringae *pv. tomato str. DC3000 [5]	AE016853	128	**ISPsy8**	**5/5**	**100%**	323
			**ISPsy7**	**6/10**	**60%**	
			ISPsy 9	1/1	100%	
			ISPsy6	1/13	7.7%	
			ISPsy5	3/36	8.3%	
*Pseudomonas aeruginosa *PAO1 [1]	AE004091	6	**ISPa11**	**6/6**	**100%**	157
*Pseudomonas putida *KT2440 [3]	AE015451	51	**ISPpu9**	**7/7**	**100%**	938
			**ISPpu10**	**7/7**	**100%**	
			ISPpu11	1/2	50%	
			ISPpu15	1/4	25%	
			ISPpu14	1/5	20%	
			Group II intron	2/9	22.2%	
*Escherichia coli *CFT073 [17]	AE014075	45	IS1397	1/2	50%	227
			IS150	1/2	50%	
*Escherichia coli *K12 [17]	U00096	44	Transp. (169aa)	1/7	14.3%	290
*Escherichia coli *O157:H7[17]	BA000007	47	IS5	1/11	9.1%	212
*Escherichia coli *O157:H7 EDL933[17]	AE005174	39	---	---	---	209
*Salmonella enterica *subsp. enterica serovar Typhi str. CT18 [55]	AL513382	29	---	---	---	228
*Salmonella typhimurium *LT2 [55]	AL513382	16	IS200	1/25	4%	207
*Shigella flexneri *2a str. 2457T [55]	AE014073	266	---	---	---	189
			IS1	6/101	6%	
			IS4	3/11	27.3%	
			IS2	2/37	5.4%	
			IS91	1/7	14.3%	
			IS3	1/6	16.7%	
*Shigella flexneri *2a str. 301 [55]	AE005674	84	ISSfl4	1/4	25%	193
			Transposase(insB)	1/1	100%	
			**IS1106**	**5/9**	**55.5%**	
*Neisseria meningitidis *MC58 [56]	AE002098	32	IS1655	2/13	15.4%	747
			**Transposase and PivNM COG3547**	**3/5**	**60%**	
*Neisseria meningitidis *Z2491 [56]	AL157959	51	IS1016C2	1/3	33.3%	673
			**IS1106**	**7/15**	**46.7%**	
			IS1655	2/6	33.3%	
			IS1016	2/17	11.8%	
*Agrobacterium tumefaciens *str. C58 UWash [1]	AE008688 AE008689	6	Transposase and PivNM COG3547	1/5	20%	116
*Agrobacterium tumefaciens *str. C58 Cereon[1]	AE007869 AE007870	5	---	---	---	121
*Sinorhizobium meliloti *1021 [1, 57]	AL591688	53	---	---	---	294
			**ISRm22**	**9/9**	**100%**	
			**ISRm19**	**2/2**	**100%**	
			ISRm11	1/6	16.6%	
			**ISRm5**	**5/7**	**71.4%**	
	AL591985 (pSymB)	11	**ISRm21**	**2/3**	**66.6%**	
			**ISRm22**	**1/1**	**100%**	
			**ISRm19**	**2/2**	**100%**	
			ISRm2011	1/2	50%	
			ISRm17	1/1	100%	
*Deinococcus radiodurans *R1 [58]	AE000513	31	---	---	---	149
*Rickettsia conorii *str. Malish 7 [59]	AE006914	0	---	---	---	237
*Mycobacterium tuberculosis *CDC1551 [1]	AE000516	37	IS1558	1/2	50%	64

13 species	19 genomes	981	36 mobile elem.	107/981	11%	

**Table 2:** Insertion Sequence elements with association with REP sequences. Table 2 shows the cases with an association ratio greater than 45% and with a total number of mobile elements in the genome equal or greater than four

IS element	IS family	Pfam-domains	Strains	Type	Assoc. ratio
**ISPsy8**	IS3	Transposase_8 (orfA) rve (orfB)	*Pseudomonas syringae pv. tomato *DC3000	**1**	**5/5**
**ISPa11**	IS110	Transposase_20 Transposase_9	*Pseudomonas aeruginosa *PA01	**1**	**6/6**
**ISPpu9**	IS110	Transposase_9 Transposase_20	*Pseudomonas putida *KT2440	**1**	**7/7**
**ISPpu10**	IS110	Transposase_9 Transposase_20	*Pseudomonas putida *KT2440	**1**	**7/7**
**ISRm22**	IS4	Transposase_11	*Sinorhizobium meliloti *1021	**1**	**10/10**
**ISRm19**	IS110	Transposase_20	*Sinorhizobium meliloti *1021	**1**	**4/4**
ISPsy7	IS110	Transposase_9 Transposase_20	*Pseudomonas syringae pv. tomato *DC3000	2	6/10
ISRm5	IS256	Transposase_mut	*Sinorhizobium meliloti *1021	2	5/7
ISNm1106	IS5	Transposase_11	*Neisseria meningitidis *MC58	2	4/7
ISNm1106	IS5	Transposase_11	*Neisseria meningitidis *Z2491	2	7/14
Transp. and PivNM COG3547	IS110	Transposase_9. Transposase_20.	*Neisseria meningitidis *MC58	2	3/5
