# Supplementary material for: Bacterial repetitive extragenic palindromic sequences are DNA targets for Insertion Sequence elements
Source: BMC Genomics. 2006 Mar 24;7:62. doi: 10.1186/1471-2164-7-62 (PMC1525189; doi:10.1186/1471-2164-7-62)
Supplement: Additional file 1 — Alignment of DNA sequences from all copies of ISPsy8 in Pseudomonas syringae DC3000 and their flanking regions. In the case named IS8-5 the broken REP element is in the plus strand and 32 bases of the REP element are upstream of the IS element while the remaining 20 bases are downstream. Symmetrically, in the four cases (IS8-1, IS8-2, IS8-3 and IS8-4) in which the REP element is placed in the minus strand, the 20 last bases of the REP sequence are in the minus strand upstream ISPsy8 and the first 31 bases of REP sequence are downstream. [file 1471-2164-7-62-S1.pdf]

```
1                       REP fragments    DR   Left End                                            orf A               130
IS8-1       TGAAAAACTTCCGCACGACAGTTGAGGTTATCGTTCCTCACGCTCCAGTGGACTGCCCCCAAGAAGTTGGACACCAATCCGACCTAATGGGGGTGTTCAAATGGCCAAGTATTCAGAGCAATTCAAGCT
IS8-2      CACGATAGTTACGCTTTAGCTCAGACACCTG-TCGTTCCTCACGCTCTGTGGACTGCCCCCAAGAAGTTGGACACCAATCCGACCTAATGGGGGTGTTCAAATGGCCAAGTATTCAGAGCAATTCAAGCT
IS8-3       TCTTCGCGAACAAGTTCGCTCCCACAGGGGCCATGTGTGCCCCGTAAATGGACTGCCCCCAAGAAGTTGGACACCAATCCGACCTAATGGGGGTGTTCAAATGGCCAAGTATTCAGAGCAATTCAAGCT
IS8-4      CGCTCAGCGTCATACGCAACTCTGAAAGAG-CCCGAAAAGCTTGGCTTTTGGACTGCCCCCAAGAAGTTGGACACCAATCCGACCTAATGGGGGTGTTCAAATGGCCAAGTATTCAGAGCAATTCAAGCT
IS8-5      CGCGTCCTCTTCGCGAC-TCAGAGCGTCGAGAGCTGCATTCCCACGCGGTGGACTGCCCCCAAGAAGTTGGACACCAATCCGACCTAATGGGGGTGTTCAAATGGCCAAGTATTCAGAGCAATTCAAGCT
Consensus  cgCgaaaccTaCgacacaaCtcagaaaggggcCgTgcaTcaCgctccagTGGACTGCCCCCAAGAAGTTGGACACCAATCCGACCTAATGGGGGTGTTCAAATGGCCAAGTATTCAGAGCAATTCAAGCT

131                                          orf A                                                                        260
IS8-1      CACCGTCGTCAAAGCCTATCTTGCAGGCAACATAGGCTTTCGCAAGGTGTCCAGCCAGTTCTCGATCGATTCCAGCCTGCTTCGACGCTGGGTCGCCAATTACAAGAGCCATGGGCACACAGGCCATCGT
IS8-2      CACCGTCGTCAAAGCCTATCTTGCAGGCAACATAGGCTTTCGCAAGGTGTCCAGCCAGTTCTCGATCGATTCCAGCCTGCTTCGACGCTGGGTCGCCAATTACAAGAGCCATGGGCACACAGGCCATCGT
IS8-3      CACCGTCGTCAAAGCCTATCTTGCAGGCAACATAGGCTTTCGCAAGGTGTCCAGCCAGTTCTCGATCGATTCCAGCCTGCTTCGACGCTGGGTCGCCAATTACAAGAGCCATGGGCACACAGGCCATCGT
IS8-4      CACCGTCGTCAAAGCCTATCTTGCAGGCAACATAGGCTTTCGCAAGGTGTCCAGCCAGTTCTCGATCGATTCCAGCCTGCTTCGACGCTGGGTCGCCAATTACAAGAGCCATGGGCACACAGGCCATCGT
IS8-5      CACCGTCGTCAAAGCCTATCTTGCAGGCAACATAGGCTTTCGCAAGGTGTCCAGCCAGTTCTCGATCGATTCCAGCCTGCTTCGACGCTGGGTCGCCAATTACAAGAGCCATGGGCACACAGGCCATCGT
Consensus  CACCGTCGTCAAAGCCTATCTTGCAGGCAACATAGGCTTTCGCAAGGTGTCCAGCCAGTTCTCGATCGATTCCAGCCTGCTTCGACGCTGGGTCGCCAATTACAAGAGCCATGGGCACACAGGCCATCGT

261                                          orf A                                                                        390
IS8-1      AAGCCTGGTCTTCGGTACAGCGAAGCCTTCAAACGCTCAGTGCTGGAGCACAAGCGCGAGCACGGGCTGTCCCTTCGCCAGACCGCAGCGCATTTTGGTATTGGTGATCCTGGCCAAATAGTCATTTGGG
IS8-2      AAGCCTGGTCTTCGGTACAGCGAAGCCTTCAAACGCTCAGTGCTGGAGCACAAGCGCGAGCACGGGCTGTCCCTTCGCCAGACCGCAGCGCATTTTGGTATTGGTGATCCTGGCCAAATAGTCATTTGGG
IS8-3      AAGCCTGGTCTTCGGTACAGCGAAGCCTTCAAACGCTCAGTGCTGGAGCACAAGCGCGAGCACGGGCTGTCCCTTCGCCAGACCGCAGCGCATTTTGGTATTGGTGATCCTGGCCAAATAGTCATTTGGG
IS8-4      AAGCCTGGTCTTCGGTACAGCGAAGCCTTCAAACGCTCAGTGCTGGAGCACAAGCGCGAGCACGGGCTGTCCCTTCGCCAGACCGCAGCGCATTTTGGTATTGGTGATCCTGGCCAAATAGTCATTTGGG
IS8-5      AAGCCTGGTCTTCGGTACAGCGAAGCCTTCAAACGCTCAGTGCTGGAGCACAAGCGCGAGCACGGGCTGTCCCTTCGCCAGACCGCAGCGCATTTTGGTATTGGTGATCCTGGCCAAATAGTCATTTGGG
Consensus  AAGCCTGGTCTTCGGTACAGCGAAGCCTTCAAACGCTCAGTGCTGGAGCACAAGCGCGAGCACGGGCTGTCCCTTCGCCAGACCGCAGCGCATTTTGGTATTGGTGATCCTGGCCAAATAGTCATTTGGG

391                                          orf A                                                                        520
IS8-1      AACAGCAGCATTACAGTAACGATCTAGCCCCCTACGTTCCCAAGAGAAGAAAGTCTGTTGCCATGCCCAAGAAGCCTTATCCCACCGCACCAGTAATCGACGACGATACGCAGAAGACGCGTGACCAGTT
IS8-2      AACAGCAGCATTACAGTAACGATCTAGCCCCCTACGTTCCCAAGAGAAGAAAGTCTGTTGCCATGCCCAAGAAGCCTTATCCCACCGCACCAGTAATCGACGACGATACGCAGAAGACGCGTGACCAGTT
IS8-3      AACAGCAGCATTACAGTAACGATCTAGCCCCCTACGTTCCCAAGAGAAGAAAGTCTGTTGCCATGCCCAAGAAGCCTTATCCCACCGCACCAGTAATCGACGACGATACGCAGAAGACGCGTGACCAGTT
IS8-4      AACAGCAGCATTACAGTAACGATCTAGCCCCCTACGTTCCCAAGAGAAGAAAGTCTGTTGCCATGCCCAAGAAGCCTTATCCCACCGCACCAGTAATCGACGACGATACGCAGAAGACGCGTGACCAGTT
IS8-5      AACAGCAGCATTACAGTAACGATCTAGCCCCCTACGTTCCCAAGAGAAGAAAGTCTGTTGCCATGCCCAAGAAGCCTTATCCCACCGCACCAGTAATCGACGACGATACGCAGAAGACGCGTGACCAGTT
Consensus  AACAGCAGCATTACAGTAACGATCTAGCCCCCTACGTTCCCAAGAGAAGAAAGTCTGTTGCCATGCCCAAGAAGCCTTATCCCACCGCACCAGTAATCGACGACGATACGCAGAAGACGCGTGACCAGTT

521                                          orf A                                          Intergenic region             650
IS8-1      AATGGCCGAGCTCGAATACCTGCGCATGGAGAACGCTTACCTAAAAAAGTTGGAAGAGTTGAAGGAGCAGCAACGACGGCAAAAGAAAAAGCCCTGATCGTCAAACAACTGAGGAGCCGCTTCTCGCTCT
IS8-2      AATGGCCGAGCTCGAATACCTGCGCATGGAGAACGCTTACCTAAAAAAGTTGGAAGAGTTGAAGGAGCAGCAACGACGGCAAAAGAAAAAGCCCTGATCGTCAAACAACTGAGGAGCCGCTTCTCGCTCT
IS8-3      AATGGCCGAGCTCGAATACCTGCGCATGGAGAACGCTTACCTAAAAAAGTTGGAAGAGTTGAAGGAGCAGCAACGACGGCAAAAGAAAAAGCCCTGATCGTCAAACAACTGAGGAGCCGCTTCTCGCTCT
IS8-4      AATGGCAGAGCTCGAATACCTGCGCATGGAGAACGCTTACCTAAAAAAGTTGGAAGAGTTGAAGGAGCAGCAACGACGGCAAAAGAAAAAGCCCTGATCGTCAAACAACTGAGGAGCCGCTTCTCGCTCT
IS8-5      AATGGCCGAGCTCGAATACCTGCGCATGGAGAACGCTTACCTAAAAAAGTTGGAAGAGTTGAAGGAGCAGCAACGACGGCAAAAGAAAAAGCCCTGATCGTCAAACAACTGAGGAGCCGCTTCTCGCTCT
Consensus  AATGGCCGAGCTCGAATACCTGCGCATGGAGAACGCTTACCTAAAAAAGTTGGAAGAGTTGAAGGAGCAGCAACGACGGCAAAAGAAAAAGCCCTGATCGTCAAACAACTGAGGAGCCGCTTCTCGCTCT

651   Intergenic region                      orf B                                                                        780
IS8-1      GCGCCCTTCTGACGCTGGCGGGCTTGGCGCGCAGTACGTTCTACTATCAGGTTCAGGTGCAATCCAGACCCGATCCAGATGCTGCTCTAAAGCAGGAGGTAGAGCGGATTTATCACGAGGAGAGAGGTCT
```

```
IS8-2      GCGCCCTTCTGACGCTGGCGGGCTTGGCGCGCAGTACGTTCTACTATCAGGTTCAGGTGCAATCCAGACCCGATCCAGATGCTGCTCTAAAGCAGGAGGTAGAGCGGATTTATCACGAGGAGAGAGGTCT
IS8-3      GCGCCCTTCTGACGCTGGCGGGCTTGGCGCGCAGTACGTTCTACTATCAGGTTCAGGTGCAATCCAGACCCGATCCAGATGCTGCTCTAAAGCAGGAGGTAGAGCGGATTTATCACGAGGAGAGAGGTCT
IS8-4      GCGCCCTTCTGACGCTGGCGGGCTTGGCGCGCAGTACGTTCTACTATCAGGTTCAGGTGCAATCCAGACCCGATCCAGATGCTGCTCTAAAGCAGGAGGTAGAGCGGATTTATCACGAGGAGAGAGGTCT
IS8-5      GCGCCCTTCTGACGCTGGCGGGCTTGGCGCGCAGTACGTTCTACTATCAGGTTCAGGTGCAATCCAGACCCGATCCAGATGCTGCTCTAAAGCAGGAGGTAGAGCGGATTTATCACGAGGAGAGAGGTCT
Consensus  GCGCCCTTCTGACGCTGGCGGGCTTGGCGCGCAGTACGTTCTACTATCAGGTTCAGGTGCAATCCAGACCCGATCCAGATGCTGCTCTAAAGCAGGAGGTAGAGCGGATTTATCACGAGGAGAGAGGTCT

           781                                                     orf B                                                              910
IS8-1      TTATGGGGCTCGGCGCATCACAGCGGTTATCCGCAACTCAGGCACGCTGGTCAACAAGAAGGTTGTGGAGAGATTGATGGCTGAACTGGGTCTGCGCTCCGTTGTTCGACCAAAAAAATACCGTTCCTAT
IS8-2      TTATGGGGCTCGGCGCATCACAGCGGTTATCCGCAACTCAGGCACGCTGGTCAACAAGAAGGTTGTGGAGAGATTGATGGCTGAACTGGGTCTGCGCTCCGTTGTTCGACCAAAAAAATACCGTTCCTAT
IS8-3      TTATGGGGCTCGGCGCATCACAGCGGTTATCCGCAACTCAGGCACGCTGGTCAACAAGAAGGTTGTGGAGAGATTGATGGCTGAACTGGGTCTGCGCTCCGTTGTTCGACCAAAAAAATACCGTTCCTAT
IS8-4      TTATGGGGCTCGGCGCATCACGGCGGTTATCCGCAACTCAGGCACGCTGGTCAACAAGAAGGTTGTGGAGAGATTGATGGCTGAACTGGGTCTGCGCTCCGTTGTTCGACCAAAAAAATACCGTTCCTAT
IS8-5      TTATGGGGCTCGGCGCATCACAGCGGTTATCCGCAACTCAGGCACGCTGGTCAACAAGAAGGTTGTGGAGAGATTGATGGCTGAACTGGGTCTGCGCTCCGTTGTTCGACCAAAAAAATACCGTTCCTAT
Consensus  TTATGGGGCTCGGCGCATCACAGCGGTTATCCGCAACTCAGGCACGCTGGTCAACAAGAAGGTTGTGGAGAGATTGATGGCTGAACTGGGTCTGCGCTCCGTTGTTCGACCAAAAAAATACCGTTCCTAT

           911                                                     orf B                                                             1040
IS8-1      AAGGGCACCGTCGGAAAAATTGCACCGAATTTGCTGGAGCGCAATTTCACGGCGCAACGCCCGAACCAGAAATGGGTGACCGATGTGACCGAGTTTAAAGTGGCCAATCGAAAGCTGTATCTCTCGCCTG
IS8-2      AAGGGCACCGTCGGAAAAATTGCACCGAATTTGCTGGAGCGCAATTTCACGGCGCAACGCCCGAACCAGAAATGGGTGACCGATGTGACCGAGTTTAAAGTGGCCAATCGAAAGCTGTATCTCTCGCCTG
IS8-3      AAGGGCACCGTCGGAAAAATTGCACCGAATTTGCTGGAGCGCAATTTCACGGCGCAACGCCCGAACCAGAAATGGGTGACCGATGTGACCGAGTTTAAAGTGGCCAATCGAAAGCTGTATCTCTCGCCTG
IS8-4      AAGGGCACCGTCGGAAAAATTGCACCGAATTTGCTGGAGCGCAATTTCACGGCGCAACGCCCGAACCAGAAATGGGTGACCGATGTGACCGAGTTTAAAGTGGCCAATCGAAAGCTGTATCTCTCGCCTG
IS8-5      AAGGGCACCGTCGGAAAAATTGCACCGAATTTGCTGGAGCGCAATTTCACGGCGCAACGCCCGAACCAGAAATGGGTGACCGATGTGACCGAGTTTAAAGTGGCCAATCGAAAGCTGTATCTCTCGCCTG
Consensus  AAGGGCACCGTCGGAAAAATTGCACCGAATTTGCTGGAGCGCAATTTCACGGCGCAACGCCCGAACCAGAAATGGGTGACCGATGTGACCGAGTTTAAAGTGGCCAATCGAAAGCTGTATCTCTCGCCTG

           1041                                                    orf B                                                             1170
IS8-1      TGATGGACCTGTACAACGGTGAGATCGTGGCGTACGAATTGTCTACCAGGCCGTGCTTTGAGCTGGTCACCAGTATGCTGGACAAGGCGTTGCAGCAGTTGCAAGACGAGCCGAAGCTTGTGATGCACTC
IS8-2      TGATGGACCTGTACAACGGTGAGATCGTGGCGTACGAATTGTCTACCAGGCCGTGCTTTGAGCTGGTCACCAGTATGCTGGACAAGGCGTTGCAGCAGTTGCAAGACGAGCCGAAGCTTGTGATGCACTC
IS8-3      TGATGGACCTGTACAACGGTGAGATCGTGGCGTACGAATTGTCTACCAGGCCGTGCTTTGAGCTGGTCACCAGTATGCTGGACAAGGCGTTGCAGCAGTTGCAAGACGAGCCGAAGCTTGTGATGCACTC
IS8-4      TGATGGACCTGTACAACGGTGAGATCGTGGCGTACGAATTGTCTACCAGGCCGTGCTTTGAGCTGGTCACCAGTATGCTGGACAAGGCGTTGCAGCAGTTGCAAGACGAGCCGAAGCTTGTGATGCACTC
IS8-5      TGATGGACCTGTACAACGGTGAGATCGTGGCGTACGAATTGTCTACCAGGCCGTGCTTTGAGCTGGTCACCAGTATGCTGGACAAGGCGTTGCAGCAGTTGCAAGACGAGCCGAAGCTTGTGATGCACTC
Consensus  TGATGGACCTGTACAACGGTGAGATCGTGGCGTACGAATTGTCTACCAGGCCGTGCTTTGAGCTGGTCACCAGTATGCTGGACAAGGCGTTGCAGCAGTTGCAAGACGAGCCGAAGCTTGTGATGCACTC

           1171                                                    orf B                                                             1300
IS8-1      GGATCAAGGCTGGCAGTATCAACACGCGCAGTACCGTCAGAAACTGGCGGCAAAGGGTGTGAAGCAAAGCATGTCTCGTAAGGGAAATTGCCTGGACAACGCTGCGATGGAAAGCTTTTTTGGTACGCTT
IS8-2      GGATCAAGGCTGGCAGTATCAACACGCGCAGTACCGTCAGAAACTGGCGGCAAAGGGTGTGAAGCAAAGCATGTCTCGTAAGGGAAATTGCCTGGACAACGCTGCGATGGAAAGCTTTTTTGGTACGCTT
IS8-3      GGATCAAGGCTGGCAGTATCAACACGCGCAGTACCGTCAGAAACTGGCGGCAAAGGGTGTGAAGCAAAGCATGTCTCGTAAGGGAAATTGCCTGGACAACGCTGCGATGGAAAGCTTTTTTGGTACGCTT
IS8-4      GGATCAAGGCTGGCAGTATCAACACGCGCAGTACCGTCAGAAACTGGCGGTAAAGGGTGTGAAGCAAAGCATGTCTCGTAAGGGAAATTGCCTGGACAACGCTGCGATGGAAAGCTTTTTTGGTACGCTT
IS8-5      GGATCAAGGCTGGCAGTATCAACACGCGCAGTACCGTCAGAAACTGGCGGCAAAGGGTGTGAAGCAAAGCATGTCTCGTAAGGGAAATTGCCTGGACAACGCTGCGATGGAAAGCTTTTTTGGTACGCTT
Consensus  GGATCAAGGCTGGCAGTATCAACACGCGCAGTACCGTCAGAAACTGGCGGCAAAGGGTGTGAAGCAAAGCATGTCTCGTAAGGGAAATTGCCTGGACAACGCTGCGATGGAAAGCTTTTTTGGTACGCTT

           1301                                                    orf B                                                             1430
IS8-1      AAGTCCGAGTTTTTCTACTTGAAACGATTTGAAAGCATAGAAGAGCTGACGGCAGGTCTGGAGGAGTACATCCGCTACTACAACCATGACCGCATTAAGCTGAAGCTAGGCGGCCTGAGCCCCGTAAAAT
IS8-2      AAGTCCGAGTTTTTCTACTTGAAACGATTTGAAAGCATAGAAGAGCTGACGGCAGGTCTGGAGGAGTACATCCGCTACTACAACCATGACCGCATTAAGCTGAAGCTAGGCGGCCTGAGCCCCGTAAAAT
IS8-3      AAGTCCGAGTTTTTCTACTTGAAACGATTTGAAAGCATAGAAGAGCTGACGGCAGGTCTGGAGGAGTACATCCGCTACTACAACCATGACCGCATTAAGCTGAAGCTAGGCGGCCTGAGCCCCGTAAAAT
IS8-4      AAGTCCGAGTTTTTCTACTTGAAACGATTTGAAAGCATAGAAGAGCTGACGGCAGGTCTGGAGGAGTACATCCGCTACTACAACCATGACCGCATTAAGCTGAAGCTAGGCGGCCTGAGCCCCGTAAAAT
IS8-5      AAGTCCGAGTTTTTCTACTTGAAACGATTTGAAAGCATAGAAGAGCTGACGGCAGGTCTGGAGGAGTACATCCGCTACTACAACCATGACCGCATTAAGCTGAAGCTAGGCGGCCTGAGCCCCGTAAAAT
```

```
Consensus  AAGTCCGAGTTTTTCTACTTGAAACGATTTGAAAGCATAGAAGAGCTGACGGCAGGTCTGGAGGAGTACATCCGCTACTACAACCATGACCGCATTAAGCTGAAGCTAGGCGGCCTGAGCCCCGTAAAAT

           1431      orf B     Right End                     DR   REP fragments                                         1555

IS8-1      ACAGGACTCAGGCGGCAAGCTAAAACTGTCCAACTTCCGGGGGGCAGTCCACAGCGTAGGAATGCCGTGGGTGACGCTCTGCGTCACAAATCTGCGCTGCGCCGCATACTCAAGACCGGACGC
IS8-2      ACAGGACTCAGGCGGCAAGCTAAAACTGTCCAACTTCCGGGGGGCAGTCCACTGCGTGGGAATGCCTTGCGTGACGCTCCGCGTCACAGGTTTGCGCAGCGTCGCCCGATAGTGCAAGGCGAT
IS8-3      ACAGGACTCAGGCGGCAAGCTAAAACTGTCCAACTTCCGGGGGGCAGTCCAAAATGTGGGAGTGACCGGGGCGGCGATCCGCATTGCTCACGAAGAGGCCGCTGCAGCCGCTTGATGTATCAG
IS8-4      ACAGGACTCAGGCGGCAAGCTAAAACTGTCCAACTTCCGGGGGGCAGTCCATTTTGTGGGAGTGGACTTGTCCGCGAT--GGGTTGCGAAGCGGCCCTAAACCAGACGACTTTGGTTGCGCCT
IS8-5      ACAGGACTCAGGCGGCAAGCTAAAACTGTCCAACTTCCGGGGGGCAGTCCACGGGGGGCGTA-GGGAACGATAGCCCTGACTATCGTGCTGAAATTCTGTGTCGATATGCTGATCTCAACTAT
Consensus  ACAGGACTCAGGCGGCAAGCTAAAACTGTCCAACTTCCGGGGGGCAGTCCAcagcGTGGGAaTGccctgGgtggCGcTccGcgTcgCaaagcagcgCtgcGcCGcacgaccgtGatcgaccat.
```
