# Supplementary material for: Bacterial repetitive extragenic palindromic sequences are DNA targets for Insertion Sequence elements
Source: BMC Genomics. 2006 Mar 24;7:62. doi: 10.1186/1471-2164-7-62 (PMC1525189; doi:10.1186/1471-2164-7-62)
Supplement: Additional File 2 — Alignment of DNA sequences from all copies of ISPa11 in Pseudomonas aeruginosa and their flanking regions. [file 1471-2164-7-62-S2.pdf]

Flanking regions upstream ISPa11 orfs

```
          1                                                                                        90
ISPa11-1  CGGATCGTGGCCGGGCGGCGGCAGCAGGAGGATGTCGATGATCGGCTTGGCGTCGAGCCCGGGCACCGCGGTGCTGCCGACATGCTCGAT
ISPa11-2    CAAGGGCGGCGGCCGGATAGTCGGCGCCTGGCCGCTGGAGGATGTTCCGCTGAGCCGCTCGCAGCGCATCGAGCTGCAACGGCAACTG
ISPa11-4   AGGTGAAGTACGTGACCTGTCTCGACCTCGGCGGCTGGGGCGCGGTGTTCACCGAGGGCTGGGACCGCCAGGTACGGTTCGTGCGCGAG
ISPa11-3       GCCGCCCTCAAGGGCTGATCCCGGGCGTACCCCGCCGAGGCGGCGGATAACCGCGTGGCGGTTATTCGCCCTACACGGGGAGGGG
ISPa11-5       CGGCGACAGCTGGTTCGTCAGCAAGGGCACCCCGGTGCTCTGGGAGGTTGCCGACGGCGGCTTCGTCAAGCACTACTACGCGGTG
ISPa11-6       AGACGCCGGTGGAAT--ACCGCAACCCGCCGCCCACCCTCGGCCAGCACACCGACGAGGTCCTCGAGACGCTCCTCGGCCTGGAC
Consensus ......g..g.Cgg..gg.....c..c....g..ccc.gg.........g.t...cg.c.gg..cc.c...g..c...ac..gc..g.g

          91                                                                                      180
ISPa11-1  GCCCAGCCCGGGCAGCGCCAGCGCCGTGCGGATGGCCTCGGCTTC-GGCGGCGAAGTGTTGT--------------GGCCAGGCGGGGTC
ISPa11-2  GCCGCCCGCGGACACGATCCGGGCGCGGTGGATGGCATCA---TC-GGCGCCAATACGCGCA--------------AGGCGATCCGCGCC
ISPa11-4  G---AGGGCAAGAAGATCAAGACCGAGATCAATGGCGTGTGGATCTACCCGCCGGCGGCGGA--------------CCGCGCCGTGGCGC
ISPa11-3  ACAAGCGGTAGGGCGAATAACGCCCCAGGCGTTATCCGCCGAAACGCCTGCCGAGGTGGCGG-----------ATAACCGCGTAGCGGTT
ISPa11-5  GCTTGAGACACCG-AAGAGACGGCGAGCCGTTACGCAAGGCGTGCGCCTGCGGCGCTGCCCAACCCTCGACACGCCCCCGTAGGGCGAAT
ISPa11-6  GCG-GCCGCGCTG-GAAAGGCTGCGCGACGGCAAGGTGATCTGAGCCCCGCGCCCCGCCCCA-----------GCGACGCATCGGCGGAC
Consensus gc....gc.....ga...a..gCg.g..gg.t.gc.......c..ccgcc.....gc..a...............c.c....g.gg.c

          181                                                                                     270
ISPa11-1  GGCAGGCAGCACCTCGATGCGGTCGTCGTCGGCGTTGCTCCAGCGTTGGCCGTCGAAGC---GGGGCGGTTGGGGTGAGGACATGGCGGA
ISPa11-2  TGCCAGCAGGAGTTCGGCTGGCCGGCGGACGGCTATCCGACCCCGGCGCTGCTCGACCG---CCTGCGGACGCCATAGGGCCGCCGGCGG
ISPa11-4  TGGCGGCGGCCGACCCGCTGATTCCGGTGGTGGCGTAGGGCGATTTTCCCTCACCCCAA---CCCTCTCCCGCAGGCGGGAGAGGATGCC
ISPa11-3  ATTCGCCC-----TACACGGGGAGAGGACAAGCGGTAGGGCGAATAACGCCCCAGGCGTTATCCGCCGAAACGCCTGCCGAGGTGGCGGA
ISPa11-5  AACCGCTCGCGGTTATCCGCCGTGAAACCCGTCGTAACGGCGGATAGCGCCTGCGGCGCTATTCGCCCTACCGGCAAGC---------CG
ISPa11-6  AACGCCCCACCGTACCCCGCGCAACACCGCGACGTAG-GGCGCATAACCGCAACGCGGTTATCCGCCGGCCAGCCGAGCATCG--GCGGA
Consensus ..ccg.c.gc.g.tc..cg.g.......cggcg.t..ggCg..t..c.cc..cg.cg....ccg.Cg..c.g....g.g.....g.gg.

          271                                            REP frg     Left End                  360

ISPa11-1  CTCCGGGGAT-----ATGCGGGAGATTGGCGGCGATGGAGAGCTGGGGTAGGGCAATGAAATGGACTCCTCCCTCCTACGGCGTCAAGCG
ISPa11-2  AGCTTACGACCCCACGTCCGCCGCGACCGCTCGCGGCACACCGGTTCGTAGGGCAATGAAATGGACTCCTCCCTCCTACGGCGTCAAGCG
ISPa11-4  GTGCAGCGTAGAGGGATGCAGCGG--CGCGCGGATGCCTGGCTTCCGGTAGGGCAATGAAATGGACTCCTCCCTCCTACGGCGTCAAGCG
ISPa11-3  TAACCGCGTGGCGGTTATTCGCCCTGCGCGGGG--AGGGAGCAAGCGGTAGGGCAATGAAATGGACTCCTCCCTCCTACGGCGTCAAGCG
ISPa11-5  TTACGCCCGGCG--------ACGCTGCCCAACCCTCGACATGTCCCCGTAGGGCAATGAAATGGACTCCTCCCTCCTACGGCGTCAAGCG
ISPa11-6  TAACGCCCCTGGCGTTATTCGCCCTACCGTACCCCGCGCAGCACCACGTAGGGCAATGAAATGGACTCCTCCCTCCTACGGCGTCAAGCG
Consensus ...c..cg...........gc.c..c...........a.c.....GTAGGGCAATGAAATGGACTCCTCCCTCCTACGGCGTCAAGCG


          361          Left End                       orf                                     450

ISPa11-1  CCAGACTAATGGTGCAGCCACAAGTCCCGGAGGGGGAGTCAGCATGAACCTTAGTCGCATTGGTCTGGATCTGGCAAAACAAGTATTCCA
ISPa11-2  CCAGACTAATGGTGCAGCCACAAGTCCCGGAGGGGGAGTCAGCATGAACCTTAGTCGCATTGGTCTGGATCTGGCAAAACAAGTATTCCA
ISPa11-4  CCAGACTAATGGTGCAGCCACAAGTCCCGGAGGGGGAGTCAGCATGAACCTTAGTCGCATTGGTCTGGATCTGGCAAAACAAGTATTCCA
ISPa11-3  CCAGACTAATGGTGCAGCCACAAGTCCCGGAGGGGGAGTCAGCATGAACCTTAGTCGCATTGGTCTGGATCTGGCAAAACAAGTATTCCA
```

```
ISPa11-5  CCAGACTAATGGTGCAGCCACAAGTCCCGGAGGGGGAGTCAGCATGAACCTTAGTCGCATTGGTCTGGATCTGGCAAAACAAGTATTCCA
ISPa11-6  CCAGACTAATGGTGCAGCCACAAGTCCCGGAGGGGGAGTCAGCATGAACCTTAGTCGCATTGGTCTGGATCTGGCAAAACAAGTATTCCA
Consensus CCAGACTAATGGTGCAGCCACAAGTCCCGGAGGGGGAGTCAGCATGAACCTTAGTCGCATTGGTCTGGATCTGGCAAAACAAGTATTCCA

          451                                      orf                                      540

ISPa11-1  GGTGCACGGCGTTGATCGTCACGAGCATGTGGTATGTCGTCGCCAACTCAAGCGGGCACAGGTGCGGGATTTCTTTCGCCAACTGCCGCC
ISPa11-2  GGTGCACGGCGTTGATCGTCACGAGCATGTGGTATGTCGTCGCCAACTCAAGCGGGCACAGGTGCGGGATTTCTTTCGCCAACTGCCGCC
ISPa11-4  GGTGCACGGCGTTGATCGTCACGAGCATGTGGTATGTCGTCGCCAACTCAAGCGGGCACAGGTGCGGGATTTCTTTCGCCAACTGCCGCC
ISPa11-3  GGTGCACGGCGTTGATCGTCACGAGCATGTGGTATGTCGTCGCCAACTCAAGCGGGCACAGGTGCGGGATTTCTTTCGCCAACTGCCGCC
ISPa11-5  GGTGCACGGCGTTGATCGTCACGAGCATGTGGTATGTCGTCGCCAACTCAAGCGGGCACAGGTGCGGGATTTCTTTCGCCAACTGCCGCC
ISPa11-6  GGTGCACGGCGTTGATCGTCACGAGCATGTGGTATGTCGTCGCCAACTCAAGCGGGCACAGGTGCGGGATTTCTTTCGCCAACTGCCGCC
Consensus GGTGCACGGCGTTGATCGTCACGAGCATGTGGTATGTCGTCGCCAACTCAAGCGGGCACAGGTGCGGGATTTCTTTCGCCAACTGCCGCC

          541                                      orf                                      630

ISPa11-1  GTGCCTGGTGGCGATGGAGGCCTGCGGCAGTGCGCACTACTGGGCGCGCGAGTTGCGGGAGCTGGGCCACACGGTACGCCTGATCGCACC
ISPa11-2  GTGCCTGGTGGCGATGGAGGCCTGCGGCAGTGCGCACTACTGGGCGCGCGAGTTGCGGGAGCTGGGCCACACGGTACGCCTGATCGCACC
ISPa11-4  GTGCCTGGTGGCGATGGAGGCCTGCGGCAGTGCGCACTACTGGGCGCGCGAGTTGCGGGAGCTGGGCCACACGGTACGCCTGATCGCACC
ISPa11-3  GTGCCTGGTGGCGATGGAGGCCTGCGGCAGTGCGCACTACTGGGCGCGCGAGTTGCGGGAGCTGGGCCACACGGTACGCCTGATCGCACC
ISPa11-5  GTGCCTGGTGGCGATGGAGGCCTGCGGCAGTGCGCACTACTGGGCGCGCGAGTTGCGGGAGCTGGGCCACACGGTACGCCTGATCGCACC
ISPa11-6  GTGCCTGGTGGCGATGGAGGCCTGCGGCAGTGCGCACTACTGGGCGCGCGAGTTGCGGGAGCTGGGCCACACGGTACGCCTGATCGCACC
Consensus GTGCCTGGTGGCGATGGAGGCCTGCGGCAGTGCGCACTACTGGGCGCGCGAGTTGCGGGAGCTGGGCCACACGGTACGCCTGATCGCACC

          631             orf             666.........................

ISPa11-1  GCAGTTCGTAAAACCCTACGTCAAGGGTGACAAGCA........................
ISPa11-2  GCAGTTCGTAAAACCCTACGTCAAGGGTGACAAGCA........................
ISPa11-4  GCAGTTCGTAAAACCCTACGTCAAGGGTGACAAGCA........................
ISPa11-3  GCAGTTCGTAAAACCCTACGTCAAGGGTGACAAGCA........................
ISPa11-5  GCAGTTCGTAAAACCCTACGTCAAGGGTGACAAGCA........................
ISPa11-6  GCAGTTCGTAAAACCCTACGTCAAGGGTGACAAGCA........................
Consensus GCAGTTCGTAAAACCCTACGTCAAGGGTGACAAGCA........................
```

Flanking regions downstream ISPa11 orfs

```
          1                                  orf                                     Right End      90

ISPa11-1  CCTGGCCAACAAGAATGCCCGGATCCTTTGGGCTTTACTCAGCCGGGAGACATGCTACCGGCCCGGTTGAGCGTTCCTGCCACACCGTAG
ISPa11-2  CCTGGCCAACAAGAATGCCCGGATCCTTTGGGCTTTACTCAGCCGGGAGACATGCTACCGGCCCGGTTGAGCGTTCCTGCCACACCGTAG
ISPa11-3  CCTGGCCAACAAGAATGCCCGGATCCTTTGGGCTTTACTCAGCCGGGAGACATGCTACCGGCCCGGTTGAGCGTTCCTGCCACACCGTAG
ISPa11-5  CCTGGCCAACAAGAATGCCCGGATCCTTTGGGCTTTACTCAGCCGGGAGACATGCTACCGGCCCGGTTGAGCGTTCCTGCCACACCGTAG
ISPa11-4  CCTGGCCAACAAGAATGCCCGGATCCTTTGGGCTTTACTCAGCCGGGAGACATGCTACCGGCCCGGTTGAGCGTTCCTGCCACACCGTAG
ISPa11-6  CCTGGCCAACAAGAATGCCCGGATCCTTTGGGCTTTACTCAGCCGGGAGACATGCTACCGGCCCGGTTGAGCGTTCCTGCCACACCGTAG
Consensus CCTGGCCAACAAGAATGCCCGGATCCTTTGGGCTTTACTCAGCCGGGAGACATGCTACCGGCCCGGTTGAGCGTTCCTGCCACACCGTAG

          91                                   Right End                                       180

ISPa11-1  TTGAAACATCCACCACGATTGCTCAGTGAATGACAATGATGACGAACCGGTCGAACCGGCCTGCATGAAACCTGGTTTATACGTGGGCTC
ISPa11-2  TTGAAACATCCACCACGATTGCTCAGTGAATGACAATGATGACGAACCGGTCGAACCGGCCTGCATGAAACCTGGTTTATACGTGGGCTC
ISPa11-3  TTGAAACATCCACCACGATTGCTCAGTGAATGACAATGATGACGAACCGGTCGAACCGGCCTGCATGAAACCTGGTTTATACGTGGGCTC
ISPa11-5  TTGAAACATCCACCACGATTGCTCAGTGAATGACAATGATGACGAACCGGTCGAACCGGCCTGCATGAAACCTGGTTTATACGTGGGCTC
ISPa11-4  TTGAAACATCCACCACGATTGCTCAGTGAATGACAATGATGACGAACCGGTCGAACCGGCCTGCATGAAACCTGGTTTATACGTGGGCTC
ISPa11-6  TTGAAACATCCACCACGATTGCTCAGTGAATGACAATGATGACGAACCGGTCGAACCGGCCTGCATGAAACCTGGTTTATACGTGGGCTC
Consensus TTGAAACATCCACCACGATTGCTCAGTGAATGACAATGATGACGAACCGGTCGAACCGGCCTGCATGAAACCTGGTTTATACGTGGGCTC

          181                                  Right End                                       270

ISPa11-1  CCTGCTGCAGTGAAGCAAAGCCGTTAGGGCGATCAGGTATGCAGGCGCGCATTTCATCAGGGCTCGGGAGTTGCAACACCACTCCATGAA
ISPa11-2  CCTGCTGCAGTGAAGCAAAGCCGTTAGGGCGATCAGGTATGCAGGCGCGCATTTCATCAGGGCTCGGGAGTTGCAACACCACTCCATGAA
ISPa11-3  CCTGCTGCAGTGAAGCAAAGCCGTTAGGGCGATCAGGTATGCAGGCGCGCATTTCATCAGGGCTCGGGAGTTGCAACACCACTCCATGAA
ISPa11-5  CCTGCTGCAGTGAAGCAAAGCCGTTAGGGCGATCAGGTATGCAGGCGCGCATTTCATCAGGGCTCGGGAGTTGCAACACCACTCCATGAA
ISPa11-4  CCTGCTGCAGTGAAGCAAAGCCGTTAGGGCGATCAGGTATGCAGGCGCGCATTTCATCAGGGCTCGGGAGTTGCAACACCACTCCATGAA
ISPa11-6  CCTGCTGCAGTGAAGCAAAGCCGTTAGGGCGATCAGGTATGCAGGCGCGCATTTCATCAGGGCTCGGGAGTTGCAACACCACTCCATGAA
Consensus CCTGCTGCAGTGAAGCAAAGCCGTTAGGGCGATCAGGTATGCAGGCGCGCATTTCATCAGGGCTCGGGAGTTGCAACACCACTCCATGAA

          271                             Right End                               REP fragment  360

ISPa11-1  GCCGGATATACGGATGCAGTCGTACACAGGTTTGAAATCAAGACAAACACTGGCAAACCGGGAGGAGTCCATATACGAATAACGCCAGCG
ISPa11-2  GCCGGATATACGGATGCAGTCGTACACAGGTTTGAAATCAAGACAAACACTGGCAAACCGGGAGGAGTCCATATACGGATAACGCCATGG
ISPa11-3  GCCGGATATACGGATGCAGTCGTACACAGGTTTGAAATCAAGACAAACACTGGCAAACCGGGAGGAGTCCATATACGAATAACGCCCCAG
ISPa11-5  GCCGGATATACGGATGCAGTCGTACACAGGTTTGAAATCAAGACAAACACTGGCAAACCGGGAGGAGTCCATATACGAATAACCGCTC-G
ISPa11-4  GCCGGATATACGGATGCAGTCGTACACAGGTTTGAAATCAAGACAAACACTGGCAAACCGGGAGGAGTCCATATACGAATAACCGCGAAG
ISPa11-6  GCCGGATATACGGATGCAGTCGTACACAGGTTTGAAATCAAGACAAACACTGGCAAACCGGGAGGAGTCCATATACGCATAACCGCAACG
Consensus GCCGGATATACGGATGCAGTCGTACACAGGTTTGAAATCAAGACAAACACTGGCAAACCGGGAGGAGTCCATATACGaATAAC..C...g
```

```
          REP fragment                                                                                      450
ISPa11-1  GCGTTATCCGCCTTCC-GCGAGCGGGGTGCGGGTAAAACCCGGGAGTCTCAGAAGTCCAGTGCCTGCACCCCTGGATGCTGCGGATCGAA
ISPa11-2  GCGTTATCCGCCGCTG-CGGCGTCCAGGACCTAGTCATCCGCGCAGAACCGCAGGATAGCGTCCGCCACCTGGGTGGCCGCCTCGCGCTG
ISPa11-3  GCGTTATCCGCCGAAACGCCTGCCGAGGTGGCGGATAACCGCGTAGCGGTTAT--TCGCCCTACACGGGGAGGGAGCAAGCGGTAGGGCG
ISPa11-5  CGGTTATCCGCCGTGAAACCCGGCGTAACGGCGCATAGCGCCTGCGGCGCTAT--TCGCCCTACTCGGCAAGCCGTTCCGCCCGGAGGCG
ISPa11-4  CGGTTATCCGCCGTTGCCTCGGTTCTCTTGGGAGCGTGCGTCTTCAGCGCTGGCGTTCCACCGCCGGACGGGCGAGCAGAAGGCGCAACG
ISPa11-6  CGGTTATCCGCCGGCCAGCCGAGCATCGGCGGATAACGCCCCTGGCGTCAT----TCGCCCTACCGACCCCGTGTAGAAGGCGGTCGATG
Consensus g.GTTATCCGCCg.....cc.g.c......g...a.a.Cc.c...g...ct....tc.ccct.C....c..g.g.....g.cg...g..g

          451                                                                                       540
ISPa11-1  CCCATCCAGGCGCATGCGCTCGGCGAATGCCTCCCGCGCCGCGTCTCCCTGGAGAAACGCGCTCCGCGCCGAGGCCCAGTCGTCCGGTTG
ISPa11-2  CGGAAAATGGCCGACGCCCTCCAGCA--CCAGCCGCTGGTAACGCCCGCTGAAGTAGTGCTCGCGTCCGCGGGAGCCGTCGGGGTGGGTG
ISPa11-3  AATAACGCCCCAGGCGTTATCCGCCGAAACGCCTGCCGAGGTGGCG--GATAACCGCGTAGCGGTTATTCGCCCTACACGGGGAGGGAGC
ISPa11-5  CTGCCCAGCCCTCGACATGTCCCCGTAGGGCGAATAACCGCTCGCG--GTTATCCGCCGTG---AAACCCGGCGTAACGGCGGATAGCGC
ISPa11-4  AGGCCCCCAGCGTTTCGCCATGACCCAGGCCGTCGAGCGGCTG-----GAAGTCCAGCGTGAGCCCGGCAACCCGGCGCAGGTCATCTAC
ISPa11-6  CCGGCGCCTCGATGGAAGCGCCACTCAGGCCGCCAGGCGGCCGCCGGCGATCGCCTCGGAGGCCTTGAGCATGGCCCGCAACAGCACCGC
Consensus c.g..c....c.......ctcc.c..a.gccgcc.....g..g.c...g....cc...g.g..c.....cg.....c....g....g..c

          541                                                                                       630
ISPa11-1  CAGCCGTACCATCCAGCCCTCGCCGTAGCAAT----CCCGGTTGATCAGCGCCGGGCGCTTGACCACCGCCGGGTTGCTCGCCAGCAGGG
ISPa11-2  CAGGCATCCGCGCCGCCGTGCAGCACCAGGGT----CGGCAGCGCCAGGCGCGGTGCCGGGAGCAAGCGCTGTTCCGCCGCCCCGTAGCG
ISPa11-3  AAGCGGTAGGGCGAATAACGCCCCCAGGCGTTATCCGCCGTAGCCTCTGCCGAGCTGGCGGTTATTCGTCCTACGCCGGGCTTTCCTCCA
ISPa11-5  CTGCGGCGCTATTCGCCCTACCGGCAAGCCGTTACGCCCGGCGACGCTGCCCAACCCTCGACATGTCCCCGTAGGGCGAA-TAACCGCTC
ISPa11-4  CAGCCGCGCCATCGCCTGGCCCGGCTCGGCTTT-CAGCGAGCCGCGCGGGCTGGCCGGTTCGGCGCTGCCGCGCCAGAGCAGGAGTTCCG
ISPa11-6  GCAGCCGGCGGCCAGGTCGTCGGGGCTGGCGTT-CTCGATCTCGTTGTGGCTGATGCCGTTCTCGCAGGGCACGAAGATCATCCCCGCCG
Consensus cagccg..c...c.......C.g....g...T..c.cc.....gc.c.Gcc..g..c......c.....c......g........c.ccg

          631                  654
ISPa11-1  TCCCGGCCAACGGGCTGCGG
ISPa11-2  CGGCTTCCCCGCGACGAAGCCC
ISPa11-3  GCGGCTGCTCGCGCAGCAG
ISPa11-5  GCGGTTATCCGCCGTGAAACCCGG
ISPa11-4  CGCGCTTTCCGCGCAGGCGCT
ISPa11-6  GACCGAGTTCGGCGAGGAAG
Consensus .....t...cgcg..g.ag.....
```
