# Supplementary material for: Bacterial repetitive extragenic palindromic sequences are DNA targets for Insertion Sequence elements
Source: BMC Genomics. 2006 Mar 24;7:62. doi: 10.1186/1471-2164-7-62 (PMC1525189; doi:10.1186/1471-2164-7-62)
Supplement: Additional File 3 — Alignment of DNA sequences from all copies of ISPpu9 in Pseudomonas putida KT2440 and their flanking regions. [file 1471-2164-7-62-S3.pdf]

```
                                                                                 REP frag.  DR                  Left End                               130
             1
1-1296422    CGGACCGGGCCCTTTCGCGGGTAAACCCGCTCCTACAGGGGACCATTGTTCCGTGTAGGAGATCACCCAGCCCTTCCGGGCTGCGCTGTCGCGCAGCAAACATGAGGTGTACGGCTTCAGTTCCGCAGCG
2-1439937    GGGTGAACCCGCTCCTACAGGTACGGCGCAGAATTCGAAATCTGTGGGGGCCCTGTAGGAGATCACCCAGCCCTTCCGGGCTGCGCTGTCGCGCAGCAAACATGAGGTGTACGGCTTCAGTTCCGCAGCG
7'-5224683   TAGCCAAATAGATGATGGTTGCCTGGGCCGGCCCTTTCGCGGGTAAACCGCCCTGTAGGAGATCACCCAGCCCTTCCGGGCTGCGCTGTCGCGCAGCAAACATGAGGTGTACGGCTTCAGTTCCGCAGCG
6'-4075799   GGTAAACCCGCTCCTACAACGTGCGGCACAGTTTTCAAAGGCTGTGACATCCCCGTAGGAGATCACCCAGCCCTTCCGGGCTGCGCTGTCGCGCAGCAAACATGAGGTGTACGGCTTCAGTTCCGCAGCG
5'-3828362    ACCGCCGGTGATGTAAAGTCGCTTCGCTGACGTGTTTTTCAGCGCCCTCGCCCGTAGGAGATCACCCAGCCCTTCCGGGCTGCGCTGTCGCGCAGCAAACATGAGGTGTACGGCTTCAGTTCCGCAGCG
Consensus    .....a....g.t.t.....g...gg.cc.gc.tt.....g......c. cCc.GTAGGAGATCACCCAGCCCTTCCGGGCTGCGCTGTCGCGCAGCAAACATGAGGTGTACGGCTTCAGTTCCGCAGCG

             131                                                   Left End                                                              .  .
1-1296422    ACGCTGACATTTTGAAAAAGAAAAGGCGGTTGGCCCGGTAGGGCCAGCCGCCTTTTGCTTTGTTAGGGTTCGTGTGCGCGGGACAGAGATCGTAGCCAGCACTGGTGTGTGTCTGCACACCCGCGTAGGA
2-1439937    ACGCTGACATTTTGAAAAAGAAAAGGCGGTTGGCCCGGTAGGGCCAGCCGCCTTTTGCTTTGTTAGGGTTCGTGTGCGCGGGACAGAGATCGTAGCCAGCACTGGTGTGTGTCTGCACACCCGCGTAGGA
7'-5224683   ACGCTGACATTTTGAAAAAGAAAAGGCGGTTGGCCCGGTAGGGCCAGCCGCCTTTTGCTTTGTTAGGGTTCGTGTGCGCGGGACAGAGATCGTAGCCAGCACTGGTGTGTGTCTGCACACCCGCGTAGGA
6'-4075799   ACGCTGACATTTTGAAAAAGAAAAGGCGGTTGGCCCGGTAGGGCCAGCCGCCTTTTGCTTTGTTAGGGTTCGTGTGCGCGGGACAGAGATCGTAGCCAGCACTGGTGTGTGTCTGCACACCCGCGTAGGA
5'-3828362   ACGCTGACATTTTGAAAAAGAAAAGGCGGTTGGCCCGGTAGGGCCAGCCGCCTTTTGCTTTGTTAGGGTTCGTGTGCGCGGGACAGAGATCGTAGCCAGCACTGGTGTGTGTCTGCACACCCGCGTAGGA
Consensus    ACGCTGACATTTTGAAAAAGAAAAGGCGGTTGGCCCGGTAGGGCCAGCCGCCTTTTGCTTTGTTAGGGTTCGTGTGCGCGGGACAGAGATCGTAGCCAGCACTGGTGTGTGTCTGCACACCCGCGTAGGA

             261                                                     ORF                                                               390
1-1296422    GAGTGATCCGCACGGTATCGGCGTGAAGTCCCTGTAGGCATCAGCTGATTCAGCTGTGACCACGTATGAGAGAGTGAACCCTTTCGCCACCGTGTGCCCCGCGCGCCCTTTATCCTGCGAGGATTCATCA
2-1439937    GAGTGATCCGCACGGTATCGGCGTGAAGTCCCTGTAGGCATCAGCTGATTCAGCTGTGACCACGTATGAGAGAGTGAACCCTTTCGCCACCGTGTGCCCCGCGCGCCCTTTATCCTGCGAGGATTCATCA
7'-5224683   GAGTGATCCGCACGGTATCGGCGTGAAGTCCCTGTAGGCATCAGCTGATTCAGCTGTGACCACGTATGAGAGAGTGAACCCTTTCGCCACCGTGTGCCCCGCGCGCCCTTTATCCTGCGAGGATTCATCA
6'-4075799   GAGTGATCCGCACGGTATCGGCGTGAAGTCCCTGTAGGCATCAGCTGATTCAGCTGTGACCACGTATGAGAGAGTGAACCCTTTCGCCACCGTGTGCCCCGCGCGCCCTTTATCCTGCGAGGATTCATCA
5'-3828362   GAGTGATCCGCACGGTATCGGCGTGAAGTCCCTGTAGGCATCAGCTGATTCAGCTGTGACCACGTATGAGAGAGTGAACCCTTTCGCCACCGTGTGCCCCGCGCGCCCTTTATCCTGCGAGGATTCATCA
Consensus    GAGTGATCCGCACGGTATCGGCGTGAAGTCCCTGTAGGCATCAGCTGATTCAGCTGTGACCACGTATGAGAGAGTGAACCCTTTCGCCACCGTGTGCCCCGCGCGCCCTTTATCCTGCGAGGATTCATCA

             391                                                     ORF                                                               520
1-1296422    TGGCGCGCAAGCCTTCCAAGCAACGCTTTACCGTCGTCCATCCCGATTGCGCGGCGATCGATGTCGGTGGTCGAGAGCATTTCGTGGCGGTCGATCCCCGGCACGAAAATCCCGTCCAGTCGTTCACGTC
2-1439937    TGGCGCGCAAGCCTTCCAAGCAACGCTTTACCGTCGTCCATCCCGATTGCGCGGCGATCGATGTCGGTGGTCGAGAGCATTTCGTGGCGGTCGATCCCCGGCACGAAAATCCCGTCCAGTCGTTCACGTC
7'-5224683   TGGCGCGCAAGCCTTCCAAGCAACGCTTTACCGTCGTCCATCCCGATTGCGCGGCGATCGATGTCGGTGGTCGAGAGCATTTCGTGGCGGTCGATCCCCGGCACGAAAATCCCGTCCAGTCGTTCACGTC
6'-4075799   TGGCGCGCAAGCCTTCCAAGCAACGCTTTACCGTCGTCCATCCCGATTGCGCGGCGATCGATGTCGGTGGTCGAGAGCATTTCGTGGCGGTCGATCCCCGGCACGAAAATCCCGTCCAGTCGTTCACGTC
5'-3828362   TGGCGCGCAAGCCTTCCAAGCAACGCTTTACCGTCGTCCATCCCGATTGCGCGGCGATCGATGTCGGTGGTCGAGAGCATTTCGTGGCGGTCGATCCCCGGCACGAAAATCCCGTCCAGTCGTTCACGTC
Consensus    TGGCGCGCAAGCCTTCCAAGCAACGCTTTACCGTCGTCCATCCCGATTGCGCGGCGATCGATGTCGGTGGTCGAGAGCATTTCGTGGCGGTCGATCCCCGGCACGAAAATCCCGTCCAGTCGTTCACGTC

             521                                                     ORF                                                               650
1-1296422    CTTTACTGACGACCTGCTCAAGATGGCTAACTGGCTTGAAAGCCTGGGGATCAAGGTCGTTGCCATGGAATCCACGGGGGTTTATTGGATTCCAATTTACGAGATTCTCAGCGAGCGCGGTTTTGACGTT
2-1439937    CTTTACTGACGACCTGCTCAAGATGGCTAACTGGCTTGAAAGCCTGGGGATCAAGGTCGTTGCCATGGAATCCACGGGGGTTTATTGGATTCCAATTTACGAGATTCTCAGCGAGCGCGGTTTTGACGTT
7'-5224683   CTTTACTGACGACCTGCTCAAGATGGCTAACTGGCTTGAAAGCCTGGGGATCAAGGTCGTTGCCATGGAATCCACGGGGGTTTATTGGATTCCAATTTACGAGATTCTCAGCGAGCGCGGTTTTGACGTT
6'-4075799   CTTTACTGACGACCTGCTCAAGATGGCTAACTGGCTTGAAAGCCTGGGGATCAAGGTCGTTGCCATGGAATCCACGGGGGTTTATTGGATTCCAATTTACGAGATTCTCAGCGAGCGCGGTTTTGACGTT
5'-3828362   CTTTACTGACGACCTGCTCAAGATGGCTAACTGGCTTGAAAGCCTGGGGATCAAGGTCGTTGCCATGGAATCCACGGGGGTTTATTGGATTCCAATTTACGAGATTCTCAGCGAGCGCGGTTTTGACGTT
Consensus    CTTTACTGACGACCTGCTCAAGATGGCTAACTGGCTTGAAAGCCTGGGGATCAAGGTCGTTGCCATGGAATCCACGGGGGTTTATTGGATTCCAATTTACGAGATTCTCAGCGAGCGCGGTTTTGACGTT
```

```
           651                                                ORF                                                         780
1-1296422  TATCTCGTCAATGCCAGAGCAACTCGGCAAATCACGGGCCGTAAATCAGATGTGCTGGATTGCCAGTGGATCTGGCAGCTGATGACTCATGGACTGCTCAGAGGCGCATTCCGCCCCGATGATCTGACCT
2-1439937  TATCTCGTCAATGCCAGAGCAACTCGGCAAATCACGGGCCGTAAATCAGATGTGCTGGATTGCCAGTGGATCTGGCAGCTGATGACTCATGGACTGCTCAGAGGCGCATTCCGCCCCGATGATCTGACCT
7'-5224683 TATCTCGTCAATGCCAGAGCAACTCGGCAAATCACGGGCCGTAAATCAGATGTGCTGGATTGCCAGTGGATCTGGCAGCTGATGACTCATGGACTGCTCAGAGGCGCATTCCGCCCCGATGATCTGACCT
6'-4075799 TATCTCGTCAATGCCAGAGCAACTCGGCAAATCACGGGCCGTAAATCAGATGTGCTGGATTGCCAGTGGATCTGGCAGCTGATGACTCATGGACTGCTCAGAGGCGCATTCCGCCCCGATGATCTGACCT
5'-3828362 TATCTCGTCAATGCCAGAGCAACTCGGCAAATCACGGGCCGTAAATCAGATGTGCTGGATTGCCAGTGGATCTGGCAGCTGATGACTCATGGACTGCTCAGAGGCGCATTCCGCCCCGATGATCTGACCT
Consensus  TATCTCGTCAATGCCAGAGCAACTCGGCAAATCACGGGCCGTAAATCAGATGTGCTGGATTGCCAGTGGATCTGGCAGCTGATGACTCATGGACTGCTCAGAGGCGCATTCCGCCCCGATGATCTGACCT

           781                                                ORF                                                         910
1-1296422  GCTGCGTCCGGTCATTGGTCAGGCAGCGTGCTTCCAAAGTGAAAGACCAGGCGCAGACGCTGAACCGGATGCAAAAGGCCATGAGCCAAATGAACATCCAGCTGGCCAATGTCATCAGTGATATTTCCGG
2-1439937  GCTGCGTCCGGTCATTGGTCAGGCAGCGTGCTTCCAAAGTGAAAGACCAGGCGCAGACGCTGAACCGGATGCAAAAGGCCATGAGCCAAATGAACATCCAGCTGGCCAATGTCATCAGTGATATTTCCGG
7'-5224683 GCTGCGTCCGGTCATTGGTCAGGCAGCGTGCTTCCAAAGTGAAAGACCAGGCGCAGACGCTGAACCGGATGCAAAAGGCCATGAGCCAAATGAACATCCAGCTGGCCAATGTCATCAGTGATATTTCCGG
6'-4075799 GCTGCGTCCGGTCATTGGTCAGGCAGCGTGCTTCCAAAGTGAAAGACCAGGCGCAGACGCTGAACCGGATGCAAAAGGCCATGAGCCAAATGAACATCCAGCTGGCCAATGTCATCAGTGATATTTCCGG
5'-3828362 GCTGCGTCCGGTCATTGGTCAGGCAGCGTGCTTCCAAAGTGAAAGACCAGGCGCAGACGCTGAACCGGATGCAAAAGGCCATGAGCCAAATGAACATCCAGCTGGCCAATGTCATCAGTGATATTTCCGG
Consensus  GCTGCGTCCGGTCATTGGTCAGGCAGCGTGCTTCCAAAGTGAAAGACCAGGCGCAGACGCTGAACCGGATGCAAAAGGCCATGAGCCAAATGAACATCCAGCTGGCCAATGTCATCAGTGATATTTCCGG

           911                                                ORF                                                        1040
1-1296422  TGTAACTGGCATGAAGATTTTGCGGGCCATCTGCGCAGGTGAACGGGACCCAGTGCAACTGGCTGAACTAACCGACCGCCGCATCAAGGCAGGCAAGGAGGCTGTCGCTCGGAGTCTTCATGGCAATTGG
2-1439937  TGTAACTGGCATGAAGATTTTGCGGGCCATCTGCGCAGGTGAACGGGACCCAGTGCAACTGGCTGAACTAACCGACCGCCGCATCAAGGCAGGCAAGGAGGCTGTCGCTCGGAGTCTTCATGGCAATTGG
7'-5224683 TGTAACTGGCATGAAGATTTTGCGGGCCATCTGCGCAGGTGAACGGGACCCAGTGCAACTGGCTGAACTAACCGACCGCCGCATCAAGGCAGGCAAGGAGGCTGTCGCTCGGAGTCTTCATGGCAATTGG
6'-4075799 TGTAACTGGCATGAAGATTTTGCGGGCCATCTGCGCAGGTGAACGGGACCCAGTGCAACTGGCTGAACTAACCGACCGCCGCATCAAGGCAGGCAAGGAGGCTGTCGCTCGGAGTCTTCATGGCAATTGG
5'-3828362 TGTAACTGGCATGAAGATTTTGCGGGCCATCTGCGCAGGTGAACGGGACCCAGTGCAACTGGCTGAACTAACCGACCGCCGCATCAAGGCAGGCAAGGAGGCTGTCGCTCGGAGTCTTCATGGCAATTGG
Consensus  TGTAACTGGCATGAAGATTTTGCGGGCCATCTGCGCAGGTGAACGGGACCCAGTGCAACTGGCTGAACTAACCGACCGCCGCATCAAGGCAGGCAAGGAGGCTGTCGCTCGGAGTCTTCATGGCAATTGG

           1041                                               ORF                                                        1170
1-1296422  CGGCGCGAGCATTTGCATGCGCTAACTCAGGAATTGGCTGCCTATGACTTCCTGGAGCAGCAAATTGCAGATTGTGACGACGCCATAAAAGCCGCGTTAGAGCAGTTGCCGGTGCTGCAAAACAAGCCAG
2-1439937  CGGCGCGAGCATTTGCATGCGCTAACTCAGGAATTGGCTGCCTATGACTTCCTGGAGCAGCAAATTGCAGATTGTGACGACGCCATAAAAGCCGCGTTAGAGCAGTTGCCGGTGCTGCAAAACAAGCCAG
7'-5224683 CGGCGCGAGCATTTGCATGCGCTAACTCAGGAATTGGCTGCCTATGACTTCCTGGAGCAGCAAATTGCAGATTGTGACGACGCCATAAAAGCCGCGTTAGAGCAGTTGCCGGTGCTGCAAAACAAGCCAG
6'-4075799 CGGCGCGAGCATTTGCATGCGCTAACTCAGGAATTGGCTGCCTATGACTTCCTGGAGCAGCAAATTGCAGATTGTGACGACGCCATAAAAGCCGCGTTAGAGCAGTTGCCGGTGCTGCAAAACAAGCCAG
5'-3828362 CGGCGCGAGCATTTGCATGCGCTAACTCAGGAATTGGCTGCCTATGACTTCCTGGAGCAGCAAATTGCAGATTGTGACGACGCCATAAAAGCCGCGTTAGAGCAGTTGCCGGTGCTGCAAAACAAGCCAG
Consensus  CGGCGCGAGCATTTGCATGCGCTAACTCAGGAATTGGCTGCCTATGACTTCCTGGAGCAGCAAATTGCAGATTGTGACGACGCCATAAAAGCCGCGTTAGAGCAGTTGCCGGTGCTGCAAAACAAGCCAG

           1171                                               ORF                                                        1300
1-1296422  AGCCATCCAAGAAGCCTTTACGGAGCCCACACCGAAACGGCGCCCAACAGACTGTATTGCATCAGACTTTGTGGAAAGTTCTTGGCGTGGACCTAACCGCAATTCCAACCATTGGGGTGGACACTGCATT
2-1439937  AGCCATCCAAGAAGCCTTTACGGAGCCCACACCGAAACGGCGCCCAACAGACTGTATTGCATCAGACTTTGTGGAAAGTTCTTGGCGTGGACCTAACCGCAATTCCAACCATTGGGGTGGACACTGCATT
7'-5224683 AGCCATCCAAGAAGCCTTTACGGAGCCCACACCGAAACGGCGCCCAACAGACTGTATTGCATCAGACTTTGTGGAAAGTTCTTGGCGTGGACCTAACCGCAATTCCAACCATTGGGGTGGACACTGCATT
6'-4075799 AGCCATCCAAGAAGCCTTTACGGAGCCCACACCGAAACGGCGCCCAACAGACTGTATTGCATCAGACTTTGTGGAAAGTTCTTGGCGTGGACCTAACCGCAATTCCAACCATTGGGGTGGACACTGCATT
5'-3828362 AGCCATCCAAGAAGCCTTTACGGAGCCCACACCGAAACGGCGCCCAACAGACTGTATTGCATCAGACTTTGTGGAAAGTTCTTGGCGTGGACCTAACCGCAATTCCAACCATTGGGGTGGACACTGCATT
Consensus  AGCCATCCAAGAAGCCTTTACGGAGCCCACACCGAAACGGCGCCCAACAGACTGTATTGCATCAGACTTTGTGGAAAGTTCTTGGCGTGGACCTAACCGCAATTCCAACCATTGGGGTGGACACTGCATT
```

```
           1301                                           ORF                                          1430
1-1296422  AGTGCTGGCAGGGGAGATCGGTACAGATCTATCACGCTTCCCGTCCTCACAGCACTTCTGCTCTTGGTTGGGACTGGCTCCCCCTACCCGAATTTCCGGCGGTCATCGACTGGCAGGTGGTGGGCCCAAA
2-1439937  AGTGCTGGCAGGGGAGATCGGTACAGATCTATCACGCTTCCCGTCCTCACAGCACTTCTGCTCTTGGTTGGGACTGGCTCCCCCTACCCGAATTTCCGGCGGTCATCGACTGGCAGGTGGTGGGCCCAAA
7′-5224683 AGTGCTGGCAGGGGAGATCGGTACAGATCTATCACGCTTCCCGTCCTCACAGCACTTCTGCTCTTGGTTGGGACTGGCTCCCCCTACCCGAATTTCCGGCGGTCATCGACTGGCAGGTGGTGGGCCCAAA
6′-4075799 AGTGCTGGCAGGGGAGATCGGTACAGATCTATCACGCTTCCCGTCCTCACAGCACTTCTGCTCTTGGTTGGGACTGGCTCCCCCTACCCGAATTTCCGGCGGTCATCGACTGGCAGGTGGTGGGCCCAAA
5′-3828362 AGTGCTGGCAGGGGAGATCGGTACAGATCTATCACGCTTCCCGTCCTCACAGCACTTCTGCTCTTGGTTGGGACTGGCTCCCCCTACCCGAATTTCCGGCGGTCATCGACTGGCAGGTGGTGGGCCCAAA
Consensus  AGTGCTGGCAGGGGAGATCGGTACAGATCTATCACGCTTCCCGTCCTCACAGCACTTCTGCTCTTGGTTGGGACTGGCTCCCCCTACCCGAATTTCCGGCGGTCATCGACTGGCAGGTGGTGGGCCCAAA

           1431                                           ORF                                          1560
1-1296422  ATAGTCAATCGAGCAGCGCAAGCACTCAAGCAGGCTGCATCCAATGCCCGTAACGACAAGGGTTTCATTGGCGCATCGCACCGAGCCAGACTGACTCGAATGGATACCAGCTGCGCCATCAAGGCCACTG
2-1439937  ATAGTCAATCGAGCAGCGCAAGCACTCAAGCAGGCTGCATCCAATGCCCGTAACGACAAGGGTTTCATTGGCGCATCGCACCGAGCCAGACTGACTCGAATGGATACCAGCTGCGCCATCAAGGCCACTG
7′-5224683 ATAGTCAATCGAGCAGCGCAAGCACTCAAGCAGGCTGCATCCAATGCCCGTAACGACAAGGGTTTCATTGGCGCATCGCACCGAGCCAGACTGACTCGAATGGATACCAGCTGCGCCATCAAGGCCACTG
6′-4075799 ATAGTCAATCGAGCAGCGCAAGCACTCAAGCAGGCTGCATCCAATGCCCGTAACGACAAGGGTTTCATTGGCGCATCGCACCGAGCCAGACTGACTCGAATGGATACCAGCTGCGCCATCAAAGCCACTG
5′-3828362 ATAGTCAATCGAGCAGCGCAAGCACTCAAGCAGGCTGCATCCAATGCCCGTAACGACAAGGGTTTCATTGGCGCATCGCACCGAGCCAGACTGACTCGAATGGATACCAGCTGCGCCATCAAAGCCACTG
Consensus  ATAGTCAATCGAGCAGCGCAAGCACTCAAGCAGGCTGCATCCAATGCCCGTAACGACAAGGGTTTCATTGGCGCATCGCACCGAGCCAGACTGACTCGAATGGATACCAGCTGCGCCATCAA.GCCACTG

           1561                                           ORF                                          1690
1-1296422  CGCATCAGTTGGCACGTCTGGTGTACAACCTGTTAACCAAGAAGCAGGCTTATGTTGAACAAGGTCTTGAGGAGTTCGAAACCAGAAGCCAAGACCGGCAGGTCCGGGCTTTGCTTCGCAAAGCCCGGAA
2-1439937  CGCATCAGTTGGCACGTCTGGTGTACAACCTGTTAACCAAGAAGCAGGCTTATGTTGAACAAGGTCTTGAGGAGTTCGAAACCAGAAGCCAAGACCGGCAGGTCCGGGCTTTGCTTCGCAAAGCCCGGAA
7′-5224683 CGCATCAGTTGGCACGTCTGGTGTACAACCTGTTAACCAAGAAGCAGGCTTATGTTGAACAAGGTCTTGAGGAGTTCGAAACCAGAAGCCAAGACCGGCAGGTCCGGGCTTTGCTTCGCAAAGCCCGGAA
6′-4075799 CGCATCAGTTGGCACGTCTGGTGTACAACCTGTTAACCAAGAAGCAGGCTTATGTTGAACAAGGTCTTGAGGAGTTCGAAGCCAGAAGCCAAGACCGGCAGGTCCGGGCTTTGCTTCGCAAAGCCCGGAA
5′-3828362 CGCATCAGTTGGCACGTCTGGTGTACAACCTGTTAACCAAGAAGCAGGCTTATGTTGAACAAGGTCTTGAGGAGTTCGAAGCCAGAAGCCAAGACCGGCAGGTCCGGGCTTTGCTTCGCAAAGCCCGGAA
Consensus  CGCATCAGTTGGCACGTCTGGTGTACAACCTGTTAACCAAGAAGCAGGCTTATGTTGAACAAGGTCTTGAGGAGTTCGAA.CCAGAAGCCAAGACCGGCAGGTCCGGGCTTTGCTTCGCAAAGCCCGGAA

           1691       ORF                                     Right End                                1820
1-1296422  ACTGGGGTATCAACTGGTGGCCGCTTGAGTGCTTAGAAAACAATGGGTTGCATTTTGTTTGATGAGAGATCACCCAGCCCTTCCGGGCTGCGCTGTCGCACAGCAAACAGGATGCGCATGACGTCAGATC
2-1439937  ACTGGGGTATCAACTGGTGGCCGCTTGAGTGCTTAGAAAACAATGGGTTGCATTTTGTTTGATGAGAGATCACCCAGCCCTTCCGGGCTGCGCTGTCGCACAGCAAACAGGATGCGCATGACGTCAGATC
7′-5224683 ACTGGGGTATCAACTGGTGGCCGCTTGAGTGCTTAGAAAACAATGGGTTGCATTTTGTTTGATGAGAGATCACCCAGCCCTTCCGGGCTGCGCTGTCGCACAGCAAACAGGATGCGCATGACGTCAGATC
6′-4075799 ACTGGGGTATCAACTGGTGGCCGCTTGAGTGCTTAGAAAACAATGGGTTGCATTTTGTTTGATGAGAGATCACCCAGCCCTTCCGGGCTGCGCTGTCGCACAGCAAACAGGATGCGCATGACGTCAGATC
5′-3828362 ACTGGGGTATCAACTGGTGGCCGCTTGAGTGCTTAGAAAACAATGGGTTGCATTTTGTTTGATGAGAGATCACCCAGCCCTTCCGGGCTGCGCTGTCGCACAGCAAACAGGATGCGCATGACGTCAGATC
Consensus  ACTGGGGTATCAACTGGTGGCCGCTTGAGTGCTTAGAAAACAATGGGTTGCATTTTGTTTGATGAGAGATCACCCAGCCCTTCCGGGCTGCGCTGTCGCACAGCAAACAGGATGCGCATGACGTCAGATC

           1821                                        Right End                                       1950
1-1296422  TGCACTGACATCGAGATCTAGAAAAGCATTCGCCGACTGATCCGGGTAGGTCAGCCGCCGAATACTTTGCTATGGTTCCCAATGCGCGGGAAAGAGGCCGTAGACCGCACTGGTGTGCTGCTTTGCTCCC
2-1439937  TGCACTGACATCGAGATCTAGAAAAGCATTCGCCGACTGATCCGGGTAGGTCAGCCGCCGAATACTTTGCTATGGTTCCCAATGCGCGGGAAAGAGGCCGTAGACCGCACTGGTGTGCTGCTTTGCTCCC
7′-5224683 TGCACTGACATCGAGATCTAGAAAAGCATTCGCCGACTGATCCGGGTAGGTCAGCCGCCGAATACTTTGCTATGGTTCCCAATGCGCGGGAAAGAGGCCGTAGACCGCACTGGTGTGCTGCTTTGCTCCC
6′-4075799 TGCACTGACATCGAGATCTAGAAAAGCATTCGCCGACTGATCCGGGTAGGTCAGCCGCCGAATACTTTGCTATGGTTCCCAATGCGCGGGAAAGAGGCCGTAGACCGCACTGGTGTGCTGCTTTGCTCCC
5′-3828362 TGCACTGACATCGAGATCTAGAAAAGCATTCGCCGACTGATCCGGGTAGGTCAGCCGCCGAATACTTTGCTATGGTTCCCAATGCGCGGGAAAGAGGCCGTAGACCGCACTGGTGTGCTGCTTTGCTCCC
Consensus  TGCACTGACATCGAGATCTAGAAAAGCATTCGCCGACTGATCCGGGTAGGTCAGCCGCCGAATACTTTGCTATGGTTCCCAATGCGCGGGAAAGAGGCCGTAGACCGCACTGGTGTGCTGCTTTGCTCCC
```

```
          1951                                         Right End                                                       2080
1-1296422 GAATGAGAGAGTGGCCCAAATGGCATCGGCGTTAATCCCTGTCGGCACAGCTTGCTGTGACCACGTATAGAAGAGTGAACACTTCGCCGCCATTGACCCCGCGCGCCTGTTTTTTTCGCTAGAAAGAACA
2-1439937 GAATGAGAGAGTGGCCCAAATGGCATCGGCGTTAATCCCTGTCGGCACAGCTTGCTGTGACCACGTATAGAAGAGTGAACACTTCGCCGCCATTGACCCCGCGCGCCTGTTTTTTTCGCTAGAAAGAACA
7'-5224683 GAATGAGAGAGTGGCCCAAATGGCATCGGCGTTAATCCCTGTCGGCACAGCTTGCTGTGACCACGTATAGAAGAGTGAACACTTCGCCGCCATTGACCCCGCGCGCCTGTTTTTTTCGCTAGAAAGAACA
6'-4075799 GAATGAGAGAGTGGCCCAAATGGCATCGGCGTTAATCCCTGTCGGCACAGCTTGCTGTGACCACGTATAGAAGAGTGAACACTTCGCCGCCATTGACCCCGCGCGCCTGTTTTTTTCGCTAGAAAGAACA
5'-3828362 GAATGAGAGAGTGGCCCAAATGGCATCGGCGTTAATCCCTGTCGGCACAGCTTGCTGTGACCACGTATAGAAGAGTGAACACTTCGCCGCCATTGACCCCGCGCGCCTGTTTTTTTCGCTAGAAAGAACA
Consensus GAATGAGAGAGTGGCCCAAATGGCATCGGCGTTAATCCCTGTCGGCACAGCTTGCTGTGACCACGTATAGAAGAGTGAACACTTCGCCGCCATTGACCCCGCGCGCCTGTTTTTTTCGCTAGAAAGAACA

          2081    Right End       DR    REP fragments     .
1-1296422 GTGGGTTATCTTTTGTTTGATAGAAGCGGGTTTACCCGCGAAGAGGCCCTGTCAGTCGCTAAAGAGTCAGAGGCCGAGCATATCCCGCGCCACTGCCTCGGCGATGCGAATGCCATCCACACCG
2-1439937 GTGGGTTATCTTTTGTTTGATAGAAGCGGGTTTACCCGCGAAAGGGCCGGCAAAGGCACCATCAAACGGTCAGCCCTTCCACACCTGCGGGTTCACCAGGTCCCGCGGCCGCTCGCCCAGCAGC
7'-5224683 GTGGGTTATCTTTTGTTTGATAGAAGCGGGTTCACCCGCGAAGAGGCCGGCCCAGCCAGCAGAGAACCGACAGATGACCGACACCCCCCTGGCAACCCTCTACCAGTCCCTCGACCAGCACCGC
6'-4075799 GTGGGTTATCTTTTGTTTGATAGAAGCGGGTTCACCCGCGAAAGGGCCAGACCAGGCAACCGAACCTTCAGGCCCGCCACCGCTCCAAGCAACACCCTGTAACGGCCCGGAACACGTTCCGGGC
5'-3828362 GTGGGTTATCTTTTGTTTGATAGAAGCGGGTTTACCCGCGAACACGGGCGAAGCCTGTGCCATCCCTAATCCCGATCCCGTAGATCTAAATCTATTAATATCAATGGCCTATGCAATATTCTTA
Consensus GTGGGTTATCTTTTGTTTGATAGAAGCGGGTT.ACCCGCGAA.agGcc.g..cag.ca.c..a...t...c....g.cc.....cc......ca.cc...t.c..g.cc...........c.gc
```

The Right Ends of the two ISPpu9 copies aligned below are shorter but the rest of the IS is identical and the sites of insertion are similar to the insertion sites of the five longer ISPpu9 copies aligned above.

```
          1                                                REP frag. DR              Left End                                  130
3-5452952 CGTTGCTGGCAGGGCGTATTGCAAAAACCGTCTGATTGACGATACGCGACCACTGTAGGAGATCACCCAGCCCTTCCGGGCTGCGCTGTCGCGCAGCAAACATGAGGTGTACGGCTTCAGTTCCGCAGCG
4'-2939439 TTCGAGGCCTGTGTTGATCCTTTCGCGGGTGAACCCGTTCCTACAGCAGTACCTGTAGGAGATCACCCAGCCCTTCCGGGCTGCGCTGTCGCGCAGCAAACATGAGGTGTACGGCTTCAGTTCCGCAGCG
Consensus ............G..G.......................C.....GC.....CTGTAGGAGATCACCCAGCCCTTCCGGGCTGCGCTGTCGCGCAGCAAACATGAGGTGTACGGCTTCAGTTCCGCAGCG

          131                                                 Left End                                                        . .
3-5452952 ACGCTGACATTTTGAAAAAGAAAAGGCGGTTGGCCCGGTAGGGCCAGCCGCCTTTTGCTTTGTTAGGGTTCGTGTGCGCGGGACAGAGATCGTAGCCAGCACTGGTGTGTGTCTGCACACCCGCGTAGGA
4'-2939439 ACGCTGACATTTTGAAAAAGAAAAGGCGGTTGGCCCGGTAGGGCCAGCCGCCTTTTGCTTTGTTAGGGTTCGTGTGCGCGGGACAGAGATCGTAGCCAGCACTGGTGTGTGTCTGCACACCCGCGTAGGA
Consensus ACGCTGACATTTTGAAAAAGAAAAGGCGGTTGGCCCGGTAGGGCCAGCCGCCTTTTGCTTTGTTAGGGTTCGTGTGCGCGGGACAGAGATCGTAGCCAGCACTGGTGTGTGTCTGCACACCCGCGTAGGA

          261                                                     ORF                                                          390
3-5452952 GAGTGATCCGCACGGTATCGGCGTGAAGTCCCTGTAGGCATCAGCTGATTCAGCTGTGACCACGTATGAGAGAGTGAACCCTTTCGCCACCGTGTGCCCCGCGCGCCCTTTATCCTGCGAGGATTCATCA
4'-2939439 GAGTGATCCGCACGGTATCGGCGTGAAGTCCCTGTAGGCATCAGCTGATTCAGCTGTGACCACGTATGAGAGAGTGAACCCTTTCGCCACCGTGTGCCCCGCGCGCCCTTTATCCTGCGAGGATTCATCA
Consensus GAGTGATCCGCACGGTATCGGCGTGAAGTCCCTGTAGGCATCAGCTGATTCAGCTGTGACCACGTATGAGAGAGTGAACCCTTTCGCCACCGTGTGCCCCGCGCGCCCTTTATCCTGCGAGGATTCATCA

          391                                                     ORF                                                          520
3-5452952 TGGCGCGCAAGCCTTCCAAGCAACGCTTTACCGTCGTCCATCCCGATTGCGCGGCGATCGATGTCGGTGGTCGAGAGCATTTCGTGGCGGTCGATCCCCGGCACGAAAATCCCGTCCAGTCGTTCACGTC
4'-2939439 TGGCGCGCAAGCCTTCCAAGCAACGCTTTACCGTCGTCCATCCCGATTGCGCGGCGATCGATGTCGGTGGTCGAGAGCATTTCGTGGCGGTCGATCCCCGGCACGAAAATCCCGTCCAGTCGTTCACGTC
Consensus TGGCGCGCAAGCCTTCCAAGCAACGCTTTACCGTCGTCCATCCCGATTGCGCGGCGATCGATGTCGGTGGTCGAGAGCATTTCGTGGCGGTCGATCCCCGGCACGAAAATCCCGTCCAGTCGTTCACGTC
```

```
521                                                    ORF                                                    650
3-5452952   CTTTACTGACGACCTGCTCAAGATGGCTAACTGGCTTGAAAGCCTGGGGATCAAGGTCGTTGCCATGGAATCCACGGGGGTTTATTGGATTCCAATTTACGAGATTCTCAGCGAGCGCGGTTTTGACGTT
4'-2939439  CTTTACTGACGACCTGCTCAAGATGGCTAACTGGCTTGAAAGCCTGGGGATCAAGGTCGTTGCCATGGAATCCACGGGGGTTTATTGGATTCCAATTTACGAGATTCTCAGCGAGCGCGGTTTTGACGTT
Consensus   CTTTACTGACGACCTGCTCAAGATGGCTAACTGGCTTGAAAGCCTGGGGATCAAGGTCGTTGCCATGGAATCCACGGGGGTTTATTGGATTCCAATTTACGAGATTCTCAGCGAGCGCGGTTTTGACGTT

651                                                    ORF                                                    780
3-5452952   TATCTCGTCAATGCCAGAGCAACTCGGCAAATCACGGGCCGTAAATCAGATGTGCTGGATTGCCAGTGGATCTGGCAGCTGATGACTCATGGACTGCTCAGAGGCGCATTCCGCCCCGATGATCTGACCT
4'-2939439  TATCTCGTCAATGCCAGAGCAACTCGGCAAATCACGGGCCGTAAATCAGATGTGCTGGATTGCCAGTGGATCTGGCAGCTGATGACTCATGGACTGCTCAGAGGCGCATTCCGCCCCGATGATCTGACCT
Consensus   TATCTCGTCAATGCCAGAGCAACTCGGCAAATCACGGGCCGTAAATCAGATGTGCTGGATTGCCAGTGGATCTGGCAGCTGATGACTCATGGACTGCTCAGAGGCGCATTCCGCCCCGATGATCTGACCT

781                                                    ORF                                                    910
3-5452952   GCTGCGTCCGGTCATTGGTCAGGCAGCGTGCTTCCAAAGTGAAAGACCAGGCGCAGACGCTGAACCGGATGCAAAAGGCCATGAGCCAAATGAACATCCAGCTGGCCAATGTCATCAGTGATATTTCCGG
4'-2939439  GCTGCGTCCGGTCATTGGTCAGGCAGCGTGCTTCCAAAGTGAAAGACCAGGCGCAGACGCTGAACCGGATGCAAAAGGCCATGAGCCAAATGAACATCCAGCTGGCCAATGTCATCAGTGATATTTCCGG
Consensus   GCTGCGTCCGGTCATTGGTCAGGCAGCGTGCTTCCAAAGTGAAAGACCAGGCGCAGACGCTGAACCGGATGCAAAAGGCCATGAGCCAAATGAACATCCAGCTGGCCAATGTCATCAGTGATATTTCCGG

911                                                    ORF                                                    1040
3-5452952   TGTAACTGGCATGAAGATTTTGCGGGCCATCTGCGCAGGTGAACGGGACCCAGTGCAACTGGCTGAACTAACCGACCGCCGCATCAAGGCAGGCAAGGAGGCTGTCGCTCGGAGTCTTCATGGCAATTGG
4'-2939439  TGTAACTGGCATGAAGATTTTGCGGGCCATCTGCGCAGGTGAACGGGACCCAGTGCAACTGGCTGAACTAACCGACCGCCGCATCAAGGCAGGCAAGGAGGCTGTCGCTCGGAGTCTTCATGGCAATTGG
Consensus   TGTAACTGGCATGAAGATTTTGCGGGCCATCTGCGCAGGTGAACGGGACCCAGTGCAACTGGCTGAACTAACCGACCGCCGCATCAAGGCAGGCAAGGAGGCTGTCGCTCGGAGTCTTCATGGCAATTGG

1041                                                   ORF                                                    1170
3-5452952   CGGCGCGAGCATTTGCATGCGCTAACTCAGGAATTGGCTGCCTATGACTTCCTGGAGCAGCAAATTGCAGATTGTGACGACGCCATAAAAGCCGCGTTAGAGCAGTTGCCGGTGCTGCAAAACAAGCCAG
4'-2939439  CGGCGCGAGCATTTGCATGCGCTAACTCAGGAATTGGCTGCCTATGACTTCCTGGAGCAGCAAATTGCAGATTGTGACGACGCCATAAAAGCCGCGTTAGAGCAGTTGCCGGTGCTGCAAAACAAGCCAG
Consensus   CGGCGCGAGCATTTGCATGCGCTAACTCAGGAATTGGCTGCCTATGACTTCCTGGAGCAGCAAATTGCAGATTGTGACGACGCCATAAAAGCCGCGTTAGAGCAGTTGCCGGTGCTGCAAAACAAGCCAG

1171                                                   ORF                                                    1300
3-5452952   AGCCATCCAAGAAGCCTTTACGGAGCCCACACCGAAACGGCGCCCAACAGACTGTATTGCATCAGACTTTGTGGAAAGTTCTTGGCGTGGACCTAACCGCAATTCCAACCATTGGGGTGGACACTGCATT
4'-2939439  AGCCATCCAAGAAGCCTTTACGGAGCCCACACCGAAACGGCGCCCAACAGACTGTATTGCATCAGACTTTGTGGAAAGTTCTTGGCGTGGACCTAACCGCAATTCCAACCATTGGGGTGGACACTGCATT
Consensus   AGCCATCCAAGAAGCCTTTACGGAGCCCACACCGAAACGGCGCCCAACAGACTGTATTGCATCAGACTTTGTGGAAAGTTCTTGGCGTGGACCTAACCGCAATTCCAACCATTGGGGTGGACACTGCATT

1301                                                   ORF                                                    1430
3-5452952   AGTGCTGGCAGGGGAGATCGGTACAGATCTATCACGCTTCCCGTCCTCACAGCACTTCTGCTCTTGGTTGGGACTGGCTCCCCCTACCCGAATTTCCGGCGGTCATCGACTGGCAGGTGGTGGGCCCAAA
4'-2939439  AGTGCTGGCAGGGGAGATCGGTACAGATCTATCACGCTTCCCGTCCTCACAGCACTTCTGCTCTTGGTTGGGACTGGCTCCCCCTACCCGAATTTCCGGCGGTCATCGACTGGCAGGTGGTGGGCCCAAA
Consensus   AGTGCTGGCAGGGGAGATCGGTACAGATCTATCACGCTTCCCGTCCTCACAGCACTTCTGCTCTTGGTTGGGACTGGCTCCCCCTACCCGAATTTCCGGCGGTCATCGACTGGCAGGTGGTGGGCCCAAA

1431                                                   ORF                                                    1560
3-5452952   ATAGTCAATCGAGCAGCGCAAGCACTCAAGCAGGCTGCATCCAATGCCCGTAACGACAAGGGTTTCATTGGCGCATCGCACCGAGCCAGACTGACTCGAATGGATACCAGCTGCGCCATCAAGGCCACTG
4'-2939439  ATAGTCAATCGAGCAGCGCAAGCACTCAAGCAGGCTGCATCCAATGCCCGTAACGACAAGGGTTTCATTGGCGCATCGCACCGAGCCAGACTGACTCGAATGGATACCAGCTGCGCCATCAAAGCCACTG
Consensus   ATAGTCAATCGAGCAGCGCAAGCACTCAAGCAGGCTGCATCCAATGCCCGTAACGACAAGGGTTTCATTGGCGCATCGCACCGAGCCAGACTGACTCGAATGGATACCAGCTGCGCCATCAA.GCCACTG

1561                                                   ORF                                                    1690
3-5452952   CGCATCAGTTGGCACGTCTGGTGTACAACCTGTTAACCAAGAAGCAGGCTTATGTTGAACAAGGTCTTGAGGAGTTCGAAACCAGAAGCCAAGACCGGCAGGTCCGGGCTTTGCTTCGCAAAGCCCGGAA
4'-2939439  CGCATCAGTTGGCACGTCTGGTGTACAACCTGTTAACCAAGAAGCAGGCTTATGTTGAACAAGGTCTTGAGGAGTTCGAAGCCAGAAGCCAAGACCGGCAGGTCCGGGCTTTGCTTCGCAAAGCCCGGAA
Consensus   CGCATCAGTTGGCACGTCTGGTGTACAACCTGTTAACCAAGAAGCAGGCTTATGTTGAACAAGGTCTTGAGGAGTTCGAA.CCAGAAGCCAAGACCGGCAGGTCCGGGCTTTGCTTCGCAAAGCCCGGAA
```

```
           1691    ORF                          Right End                                          DR  REP fragments                .
3-5452952  ACTGGGGTATCAACTGGTGGCCGCTTGAGTGCTTAGAAAACAATGGGTTGCATTTTGTTTGATGAGAGCGGGTTTACCCGCGAATACGGTAGTCGCGGCAACGGTGAACGGCGGGTGGAGATTGGCCAGC
4’-2939439 ACTGGGGTATCAACTGGTGGCCGCTTGAGTGCTTAGAAAACAATGGGTTGCATTTTGTTTGATGAGAGCGGGTTCACCCGCGAAGGGGCCGGCACAGGCAAACTCGACCTTCAGGTGTGAGTGATATCCC
Consensus  ACTGGGGTATCAACTGGTGGCCGCTTGAGTGCTTAGAAAACAATGGGTTGCATTTTGTTTGATGAGAGCGGGTT.ACCCGCGAA...G...G....GGCAA....GA.C..C.GGTG....T.......C
```
