# Supplementary material for: Bacterial repetitive extragenic palindromic sequences are DNA targets for Insertion Sequence elements
Source: BMC Genomics. 2006 Mar 24;7:62. doi: 10.1186/1471-2164-7-62 (PMC1525189; doi:10.1186/1471-2164-7-62)
Supplement: Additional File 4 — Alignment of DNA sequences from all copies of ISPpu10 in Pseudomonas putida KT2440 and their flanking regions. [file 1471-2164-7-62-S4.pdf]

```
             1                                                   REP fragment            DR  Left End                                            130

1-610781-6   AAAGGGCCGGGCCTGCTGGCCATTTCTCACCAGTCGTTCACCGTTGCCGCCGCTACCGTATTCGCGGGTAAACCCGCTTGTATGGTAGGACTTGAAGGGAGGAGGAGGGCCATAACTCGCTTCTGACTGT
6-5756681-   AAGAGGCCGGGCCTGCTGGCCAATCTCCACCTGCCGTTCAGCGTTGCCGCCACCACCGTATTCGCGGGTAAACCCGCTTGTATGGTAGGACTTGAAGGGAGGAGGAGGGCCATAACTCGCTTCTGACTGT
2-1846955-   GCAGAATGGCAAACGCGGGTTGCGCTTGTGGATGAGGTTGCCGTTGCATGTGCCGGCCTCTTCGCGGGTAAACCCGCTTGTATGGTAGGACTTGAAGGGAGGAGGAGGGCCATAACTCGCTTCTGACTGT
5-5218778-   GCCTTGTTGCCGGTGGGGTATGTGTTGAGCTGACGGTGGGTGGTTGCCTGTTCTGGCCTCTTCGCGGGTAAACCCGCTTGTATGGTAGGACTTGAAGGGAGGAGGAGGGCCATAACTCGCTTCTGACTGT
4-3971673-   AATACGGTAGCCGCGACAATGGTGAACGGCAGGTGGACATTGGCTAGTAGGCCCGGCTCTTTCGCGGGTAAACCCGCTTGTATGGTAGGACTTGAAGGGAGGAGGAGGGCCATAACTCGCTTCTGACTGT
7'-6039251    ATGAAAAAGGCGACCTTCTTCAGAGGGTCGCCTTTTTTTGTGCCGCCTGATCTGGCCCTTTCGCGGGTAAACCCGCTTGTATGGTAGGACTTGAAGGGAGGAGGAGGGCCATAACTCGCTTCTGACTGT
3-2435133-   GTAGGCGCGGATTCATCTGCGAACACTGGCAGAGCCGGTGCCAAACTCCGCGGTGCCTGCTTCGCGGGTAAACCCGCTTGTATGGTAGGACTTGAAGGGAGGAGGAGGGCCATAACTCGCTTCTGACTGT
 Consensus   .......gg....g.............c.....g......g.tgcc.g..c.g.C...TTCGCGGGTAAACCCGCTTGTATGGTAGGACTTGAAGGGAGGAGGAGGGCCATAACTCGCTTCTGACTGT

             131                                               Left End                                                                260

1-610781-6   TCGCGCAGTACAGACCGTAGGAGAAATCACCCCTCCTCCTTTCAACACCCAAGCGCCGATAAGGAATGCCTCGCGAGCAAGCGACAATAGAAGCAAGCCTGCGCCAGGGTGTGCCCTTCAAGTCACACTA
6-5756681-   TCGCGCAGTACAGACCGTAGGAGAAATCACCCCTCCTCCTTTCAACACCCAAGCGCCGATAAGGAATGCCTCGCGAGCAAGCGACAATAGAAGCAAGCCTGCGCCAGGGTGTGCCCTTCAAGTCACACTA
2-1846955-   TCGCGCAGTACAGACCGTAGGAGAAATCACCCCTCCTCCTTTCAACACCCAAGCGCCGATAAGGAATGCCTCGCGAGCAAGCGACAATAGAAGCAAGCCTGCGCCAGGGTGTGCCCTTCAAGTCACACTA
5-5218778-   TCGCGCAGTACAGACCGTAGGAGAAATCACCCCTCCTCCTTTCAACACCCAAGCGCCGATAAGGAATGCCTCGCGAGCAAGCGACAATAGAAGCAAGCCTGCGCCAGGGTGTGCCCTTCAAGTCACACTA
4-3971673-   TCGCGCAGTACAGACCGTAGGAGAAATCACCCCTCCTCCTTTCAACACCCAAGCGCCGATAAGGAATGCCTCGCGAGCAAGCGACAATAGAAGCAAGCCTGCGCCAGGGTGTGCCCTTCAAGTCACACTA
7'-6039251   TCGCGCAGTACAGACCGTAGGAGAAATCACCCCTCCTCCTTTCAACACCCAAGCGCCGATAAGGAATGCCTCGCGAGCAAGCGACAATAGAAGCAAGCCTGCGCCAGGGTGTGCCCTTCAAGTCACACTA
3-2435133-   TCGCGCAGTACAGACCGTAGGAGAAATCACCCCTCCTCCTTTCAACACCCAAGCGCCGATAAGGAATGCCTCGCGAGCAAGCGACAATAGAAGCAAGCCTGCGCCAGGGTGTGCCCTTCAAGTCACACTA
 Consensus   TCGCGCAGTACAGACCGTAGGAGAAATCACCCCTCCTCCTTTCAACACCCAAGCGCCGATAAGGAATGCCTCGCGAGCAAGCGACAATAGAAGCAAGCCTGCGCCAGGGTGTGCCCTTCAAGTCACACTA

             261   Left End            orf                                                                                            390

1-610781-6   CGCTACAAGGAGCAGCTATTCATGGCAATGTCCGCAATCCCAATCGAAGCTGGCGTGGACACCTCCAAAGATGAACTGGTGATTCAAGCCGCTCCGAATACCAAATCCTTCGCCATCCCTAACACTCCGA
6-5756681-   CGCTACAAGGAGCAGCTATTCATGGCAATGTCCGCAATCCCAATCGAAGCTGGCGTGGACACCTCCAAAGATGAACTGGTGATTCAAGCCGCTCCGAATACCAAATCCTTCGCCATCCCTAACACTCCGA
2-1846955-   CGCTACAAGGAGCAGCTATTCATGGCAATGTCCGCAATCCCAATCGAAGCTGGCGTGGACACCTCCAAAGATGAACTGGTGATTCAAGCCGCTCCGAATACCAAATCCTTCGCCATCCCTAACACTCCGA
5-5218778-   CGCTACAAGGAGCAGCTATTCATGGCAATGTCCGCAATCCCAATCGAAGCTGGCGTGGACACCTCCAAAGATGAACTGGTGATTCAAGCCGCTCCGAATACCAAATCCTTCGCCATCCCTAACACTCCGA
4-3971673-   CGCTACAAGGAGCAGCTATTCATGGCAATGTCCGCAATCCCAATCGAAGCTGGCGTGGACACCTCCAAAGATGAACTGGTGATTCAAGCCGCTCCGAATACCAAATCCTTCGCCATCCCTAACACTCCGA
7'-6039251   CGCTACAAGGAGCAGCTATTCATGGCAATGTCCGCAATCCCAATCGAAGCTGGCGTGGACACCTCCAAAGATGAACTGGTGATTCAAGCCGCTCCGAATACCAAATCCTTCGCCATCCCTAACACTCCGA
3-2435133-   CGCTACAAGGAGCAGCTATTCATGGCAATGTCCGCAATCCCAATCGAAGCTGGCGTGGACACCTCCAAAGATGAACTGGTGATTCAAGCCGCTCCGAATACCAAATCCTTCGCCATCCCTAACACTCCGA
 Consensus   CGCTACAAGGAGCAGCTATTCATGGCAATGTCCGCAATCCCAATCGAAGCTGGCGTGGACACCTCCAAAGATGAACTGGTGATTCAAGCCGCTCCGAATACCAAATCCTTCGCCATCCCTAACACTCCGA

             392                                                  orf                                                                 520
1-610781-6   AAGCAATCAAAGCCTGGCTCAAGACGCTGCCCAAAGGCTCAGCGCTGGCCATCGAAGCGACCAGTACCTATCACATGGAAATGGCGGAGCAAGCCCATGCTGCGGGCTTCGCGGTATACGTTATCGATGG
6-5756681-   AAGCAATCAAAGCCTGGCTCAAGACGCTGCCCAAAGGCTCAGCGCTGGCCATCGAAGCGACCAGTACCTATCACATGGAAATGGCGGAGCAAGCCCATGCTGCGGGCTTCGCGGTATACGTTATCGATGG
2-1846955-   AAGCAATCAAAGCCTGGCTCAAGACGCTGCCCAAAGGCTCAGCGCTGGCCATCGAAGCGACCAGTACCTATCACATGGAAATGGCGGAGCAAGCCCATGCTGCGGGCTTCGCGGTATACGTTATCGATGG
5-5218778-   AAGCAATCAAAGCCTGGCTCAAGACGCTGCCCAAAGGCTCAGCGCTGGCCATCGAAGCGACCAGTACCTATCACATGGAAATGGCGGAGCAAGCCCATGCTGCGGGCTTCGCGGTATACGTTATCGATGG
4-3971673-   AAGCAATCAAAGCCTGGCTCAAGACGCTGCCCAAAGGCTCAGCGCTGGCCATCGAAGCGACCAGTACCTATCACATGGAAATGGCGGAGCAAGCCCATGCTGCGGGCTTCGCGGTATACGTTATCGATGG
```

```
7’-6039251  AAGCAATCAAAGCCTGGCTCAAGACGCTGCCCAAAGGCTCAGCGCTGGCCATCGAAGCGACCAGTACCTATCACATGGAAATGGCGGAGCAAGCCCATGCTGCGGGCTTCGCGGTATACGTTATCGATGG
3-2435133-  AAGCAATCAAAGCCTGGCTCAAGACGCTGCCCAAAGGCTCAGCGCTGGCCATCGAAGCGACCAGTACCTATCACATGGAAATGGCGGAGCAAGCCCATGCTGCGGGCTTCGCGGTATACGTTATCGATGG
 Consensus  AAGCAATCAAAGCCTGGCTCAAGACGCTGCCCAAAGGCTCAGCGCTGGCCATCGAAGCGACCAGTACCTATCACATGGAAATGGCGGAGCAAGCCCATGCTGCGGGCTTCGCGGTATACGTTATCGATGG

            521                                                            orf                                                            650
1-610781-6  GCTGAGGCTGAGTAAGTACCGGGAAAGTGTTGCTATACGGGCAAAAACAGATGCCCATGATGCCGCTTTGCTCGCCCGCTTCCTGAGCAACGAGCGGGGCTCTTTGAAGGCTTGGACTCCGCCACCAGCC
6-5756681-  GCTGAGGCTGAGTAAGTACCGGGAAAGTGTTGCTATACGGGCAAAAACAGATGCCCATGATGCCGCTTTGCTCGCCCGCTTCCTGAGCAACGAGCGGGGCTCTTTGAAGGCTTGGACTCCGCCACCAGCC
2-1846955-  GCTGAGGCTGAGTAAGTACCGGGAAAGTGTTGCTATACGGGCAAAAACAGATGCCCATGATGCCGCTTTGCTCGCCCGCTTCCTGAGCAACGAGCGGGGCTCTTTGAAGGCTTGGACTCCGCCACCAGCC
5-5218778-  GCTGAGGCTGAGTAAGTACCGGGAAAGTGTTGCTATACGGGCAAAAACAGATGCCCATGATGCCGCTTTGCTCGCCCGCTTCCTGAGCAACGAGCGGGGCTCTTTGAAGGCTTGGACTCCGCCACCAGCC
4-3971673-  GCTGAGGCTGAGTAAGTACCGGGAAAGTGTTGCTATACGGGCAAAAACAGATGCCCATGATGCCGCTTTGCTCGCCCGCTTCCTGAGCAACGAGCGGGGCTCTTTGAAGGCTTGGACTCCGCCACCAGCC
7’-6039251  GCTGAGGCTGAGTAAGTACCGGGAAAGTGTTGCTATACGGGCAAAAACAGATGCCCATGATGCCGCTTTGCTCGCCCGCTTCCTGAGCAACGAGCGGGGCTCTTTGAAGGCTTGGACTCCGCCACCAGCC
3-2435133-  GCTGAGGCTGAGTAAGTACCGGGAAAGTGTTGCTATACGGGCAAAAACAGATGCCCATGATGCCGCTTTGCTCGCCCGCTTCCTGAGCAACGAGCGGGGCTCTTTGAAGGCTTGGACTCCGCCACCAGCC
 Consensus  GCTGAGGCTGAGTAAGTACCGGGAAAGTGTTGCTATACGGGCAAAAACAGATGCCCATGATGCCGCTTTGCTCGCCCGCTTCCTGAGCAACGAGCGGGGCTCTTTGAAGGCTTGGACTCCGCCACCAGCC

            651                                                            orf                                                            780
1-610781-6  GGGCATCGTGAGATCCAGGTGCTGCTGCGCCGCCGCGCCAAACTTGTAGCAGTGCGCGGCATGCTGCGGATGAGCTTGTCGGGCGACAAATTGTTTGCCTCAGAACTCAAACGTGTTGAGGAAGTGCTCG
6-5756681-  GGGCATCGTGAGATCCAGGTGCTGCTGCGCCGCCGCGCCAAACTTGTAGCAGTGCGCGGCATGCTGCGGATGAGCTTGTCGGGCGACAAATTGTTTGCCTCAGAACTCAAACGTGTTGAGGAAGTGCTCG
2-1846955-  GGGCATCGTGAGATCCAGGTGCTGCTGCGCCGCCGCGCCAAACTTGTAGCAGTGCGCGGCATGCTGCGGATGAGCTTGTCGGGCGACAAATTGTTTGCCTCAGAACTCAAACGTGTTGAGGAAGTGCTCG
5-5218778-  GGGCATCGTGAGATCCAGGTGCTGCTGCGCCGCCGCGCCAAACTTGTAGCAGTGCGCGGCATGCTGCGGATGAGCTTGTCGGGCGACAAATTGTTTGCCTCAGAACTCAAACGTGTTGAGGAAGTGCTCG
4-3971673-  GGGCATCGTGAGATCCAGGTGCTGCTGCGCCGCCGCGCCAAACTTGTAGCAGTGCGCGGCATGCTGCGGATGAGCTTGTCGGGCGACAAATTGTTTGCCTCAGAACTCAAACGTGTTGAGGAAGTGCTCG
7’-6039251  GGGCATCGTGAGATCCAGGTGCTGCTGCGCCGCCGCGCCAAACTTGTAGCAGTGCGCGGCATGCTGCGGATGAGCTTGTCGGGCGACAAATTGTTTGCCTCAGAACTCAAACGTGTTGAGGAAGTGCTCG
3-2435133-  GGGCATCGTGAGATCCAGGTGCTGCTGCGCCGCCGCGCCAAACTTGTAGCAGTGCGCGGCATGCTGCGGATGAGCTTGTCGGGCGACAAATTGTTTGCCTCAGAACTCAAACGTGTTGAGGAAGTGCTCG
 Consensus  GGGCATCGTGAGATCCAGGTGCTGCTGCGCCGCCGCGCCAAACTTGTAGCAGTGCGCGGCATGCTGCGGATGAGCTTGTCGGGCGACAAATTGTTTGCCTCAGAACTCAAACGTGTTGAGGAAGTGCTCG

            781                                                            orf                                                            910
1-610781-6  AACGCATAGAGCTTTCGCTGGAAAAACAGCTGCGTGCGGTGATCAAGAAGGCGGGGCTTTCCGATCAGATGCGCCGTGTTCAGAGGCTTCCGGGTGTTGGTTTCTTAACGGCTGCCGGCCTGGTGATGTC
6-5756681-  AACGCATAGAGCTTTCGCTGGAAAAACAGCTGCGTGCGGTGATCAAGAAGGCGGGGCTTTCCGATCAGATGCGCCGTGTTCAGAGGCTTCCGGGTGTTGGTTTCTTAACGGCTGCCGGCCTGGTGATGTC
2-1846955-  AACGCATAGAGCTTTCGCTGGAAAAACAGCTGCGTGCGGTGATCAAGAAGGCGGGGCTTTCCGATCAGATGCGCCGTGTTCAGAGGCTTCCGGGTGTTGGTTTCTTAACGGCTGCCGGCCTGGTGATGTC
5-5218778-  AACGCATAGAGCTTTCGCTGGAAAAACAGCTGCGTGCGGTGATCAAGAAGGCGGGGCTTTCCGATCAGATGCGCCGTGTTCAGAGGCTTCCGGGTGTTGGTTTCTTAACGGCTGCCGGCCTGGTGATGTC
4-3971673-  AACGCATAGAGCTTTCGCTGGAAAAACAGCTGCGTGCGGTGATCAAGAAGGCGGGGCTTTCCGATCAGATGCGCCGTGTTCAGAGGCTTCCGGGTGTTGGTTTCTTAACGGCTGCCGGCCTGGTGATGTC
7’-6039251  AACGCATAGAGCTTTCGCTGGAAAAACAGCTGCGTGCGGTGATCAAGAAGGCGGGGCTTTCCGATCAGATGCGCCGTGTTCAGAGGCTTCCGGGTGTTGGTTTCTTAACGGCTGCCGGCCTGGTGATGTC
3-2435133-  AACGCATAGAGCTTTCGCTGGAAAAACAGCTGCGTGCGGTGATCAAGAAGGCGGGGCTTTCCGATCAGATGCGCCGTGTTCAGAGGCTTCCGGGTGTTGGTTTCTTAACGGCTGCCGGCCTGGTGATGTC
 Consensus  AACGCATAGAGCTTTCGCTGGAAAAACAGCTGCGTGCGGTGATCAAGAAGGCGGGGCTTTCCGATCAGATGCGCCGTGTTCAGAGGCTTCCGGGTGTTGGTTTCTTAACGGCTGCCGGCCTGGTGATGTC

            911                                                            orf                                                           1040
1-610781-6  CTTCATGCGCGGCGAGTTCAAGAACAGCGACGCCTTTGTCGCTTATCTAGGCATGGATGTAACGGTTTCCCAATCCGGTAAATGGGCAGGCAGAGGGAAGTTGAGCAAGCGCGGAGACTCGGAGGTCCGT
6-5756681-  CTTCATGCGCGGCGAGTTCAAGAACAGCGACGCCTTTGTCGCTTATCTAGGCATGGATGTAACGGTTTCCCAATCCGGTAAATGGGCAGGCAGAGGGAAGTTGAGCAAGCGCGGAGACTCGGAGGTCCGT
2-1846955-  CTTCATGCGCGGCGAGTTCAAGAACAGCGACGCCTTTGTCGCTTATCTAGGCATGGATGTAACGGTTTCCCAATCCGGTAAATGGGCAGGCAGAGGGAAGTTGAGCAAGCGCGGAGACTCGGAGGTCCGT
5-5218778-  CTTCATGCGCGGCGAGTTCAAGAACAGCGACGCCTTTGTCGCTTATCTAGGCATGGATGTAACGGTTTCCCAATCCGGTAAATGGGCAGGCAGAGGGAAGTTGAGCAAGCGCGGAGACTCGGAGGTCCGT
4-3971673-  CTTCATGCGCGGCGAGTTCAAGAACAGCGACGCCTTTGTCGCTTATCTAGGCATGGATGTAACGGTTTCCCAATCCGGTAAATGGGCAGGCAGAGGGAAGTTGAGCAAGCGCGGAGACTCGGAGGTCCGT
7’-6039251  CTTCATGCGCGGCGAGTTCAAGAACAGCGACGCCTTTGTCGCTTATCTAGGCATGGATGTAACGGTTTCCCAATCCGGTAAATGGGCAGGCAGAGGGAAGTTGAGCAAGCGCGGAGACTCGGAGGTCCGT
3-2435133-  CTTCATGCGCGGCGAGTTCAAGAACAGCGACGCCTTTGTCGCTTATCTAGGCATGGATGTAACGGTTTCCCAATCCGGTAAATGGGCAGGCAGAGGGAAGTTGAGCAAGCGCGGAGACTCGGAGGTCCGT
 Consensus  CTTCATGCGCGGCGAGTTCAAGAACAGCGACGCCTTTGTCGCTTATCTAGGCATGGATGTAACGGTTTCCCAATCCGGTAAATGGGCAGGCAGAGGGAAGTTGAGCAAGCGCGGAGACTCGGAGGTCCGT
```

```
           1041                                  orf                                                                        1170
1-610781-6 AGACTCCTGTACAACGCCTCGATGAGCGGCAGTCGAACCGCGACCTGGAAGCAGTATTACGCCCATCACCAAGCCCGGGGGAAGAAAACCACTCAAGCCTTGGTGATCCTTGCACGTCGCTTGGCTCGGC
6-5756681- AGACTCCTGTACAACGCCTCGATGAGCGGCAGTCGAACCGCGACCTGGAAGCAGTATTACGCCCATCACCAAGCCCGGGGGAAGAAAACCACTCAAGCCTTGGTGATCCTTGCACGTCGCTTGGCTCGGC
2-1846955- AGACTCCTGTACAACGCCTCGATGAGCGGCAGTCGAACCGCGACCTGGAAGCAGTATTACGCCCATCACCAAGCCCGGGGGAAGAAAACCACTCAAGCCTTGGTGATCCTTGCACGTCGCTTGGCTCGGC
5-5218778- AGACTCCTGTACAACGCCTCGATGAGCGGCAGTCGAACCGCGACCTGGAAGCAGTATTACGCCCATCACCAAGCCCGGGGGAAGAAAACCACTCAAGCCTTGGTGATCCTTGCACGTCGCTTGGCTCGGC
4-3971673- AGACTCCTGTACAACGCCTCGATGAGCGGCAGTCGAACCGCGACCTGGAAGCAGTATTACGCCCATCACCAAGCCCGGGGGAAGAAAACCACTCAAGCCTTGGTGATCCTTGCACGTCGCTTGGCTCGGC
7'-6039251 AGACTCCTGTACAACGCCTCGATGAGCGGCAGTCGAACCGCGACCTGGAAGCAGTATTACGCCCATCACCAAGCCCGGGGGAAGAAAACCACTCAAGCCTTGGTGATCCTTGCACGTCGCTTGGCTCGGC
3-2435133- AGACTCCTGTACAACGCCTCGATGAGCGGCAGTCGAACCGCGACCTGGAAGCAGTATTACGCCCATCACCAAGCCCGGGGGAAGAAAACCACTCAAGCCTTGGTGATCCTTGCACGTCGCTTGGCTCGGC
 Consensus AGACTCCTGTACAACGCCTCGATGAGCGGCAGTCGAACCGCGACCTGGAAGCAGTATTACGCCCATCACCAAGCCCGGGGGAAGAAAACCACTCAAGCCTTGGTGATCCTTGCACGTCGCTTGGCTCGGC

           1171                          orf                                         Right End                                      1300
1-610781-6 TGGCCTTCGGCCTGATGAGGCATCAGGCCGACTGGAAGCCCGAGATATATACCGGAGGTGCCAAGCCGGCCAGCTGAGGCTGGGGCTGCAACGCAGCCCGCCCAAACTGACGGGCAGTATCCAATGCTGG
6-5756681- TGGCCTTCGGCCTGATGAGGCATCAGGCCGACTGGAAGCCCGAGATATATACCGGAGGTGCCAAGCCGGCCAGCTGAGGCTGGGGCTGCAACGCAGCCCGCCCAAACTGACGGGCAGTATCCAATGCTGG
2-1846955- TGGCCTTCGGCCTGATGAGGCATCAGGCCGACTGGAAGCCCGAGATATATACCGGAGGTGCCAAGCCGGCCAGCTGAGGCTGGGGCTGCAACGCAGCCCGCCCAAACTGACGGGCAGTATCCAATGCTGG
5-5218778- TGGCCTTCGGCCTGATGAGGCATCAGGCCGACTGGAAGCCCGAGATATATACCGGAGGTGCCAAGCCGGCCAGCTGAGGCTGGGGCTGCAACGCAGCCCGCCCAAACTGACGGGCAGTATCCAATGCTGG
4-3971673- TGGCCTTCGGCCTGATGAGGCATCAGGCCGACTGGAAGCCCGAGATATATACCGGAGGTGCCAAGCCGGCCAGCTGAGGCTGGGGCTGCAACGCAGCCCGCCCAAACTGACGGGCAGTATCCAATGCTGG
7'-6039251 TGGCCTTCGGCCTGATGAGGCATCAGGCCGACTGGAAGCCCGAGATATATACCGGAGGTGCCAAGCCGGCCAGCTGAGGCTGGGGCTGCAACGCAGCCCGCCCAAACTGACGGGCAGTATCCAATGCTGG
3-2435133- TGGCCTTCGGCCTGATGAGGCATCAGGCCGACTGGAAGCCCGAGATATATACCGGAGGTGCCAAGCCGGCCAGCTGAGGCTGGGGCTGCAACGCAGCCCGCCCAAACTGACGGGCAGTATCCAATGCTGG
 Consensus TGGCCTTCGGCCTGATGAGGCATCAGGCCGACTGGAAGCCCGAGATATATACCGGAGGTGCCAAGCCGGCCAGCTGAGGCTGGGGCTGCAACGCAGCCCGCCCAAACTGACGGGCAGTATCCAATGCTGG

           1301                                  Right End                                      DR REP frag.                        1430
1-610781-6 GGAGAAATTCTCTGGCACGCTGACTCGTACCTCGAGGTTGCTGAGAGAGGCCGATGGCGTTTTTACTGAAAATTTGCCTCCGGCCATAGAATCTCCTACAGTGGGCACACGTCGGTGATCGGGCGCAGGC
6-5756681- GGAGAAATTCTCTGGCACGCTGACTCGTACCTCGAGGTTGCTGAGAGAGGCCGATGGCGTTTTTACTGAAAATTTGCCTCCGGCCATAGAATCTCCTACCCCAATCCTGTGGGAGCGGCCTTGTGTCGCG
2-1846955- GGAGAAATTCTCTGGCACGCTGACTCGTACCTCGAGGTTGCTGAGAGAGGCCGATGGCGTTTTTACTGAAAATTTGCCTCCGGCCATAGAATCTCCTACAGGCTTGCGAATCCCCTGTAGGAGCGGGTTC
5-5218778- GGAGAAATTCTCTGGCACGCTGACTCGTACCTCGAGGTTGCTGAGAGAGGCCGATGGCGTTTTTACTGAAAATTTGCCTCCGGCCATAGAATCTCCTACATCGGCAACAGGTGTTTGACGAAGTTGCATG
4-3971673- GGAGAAATTCTCTGGCACGCTGACTCGTACCTCGAGGTTGCTGAGAGAGGCCGATGGCGTTTTTACTGAAAATTTGCCTCCGGCCATAGAATCTCCTACAGGGGCTTGTGGTTTCCCTTTGAGAGCGCCG
7'-6039251 GGAGAAATTCTCTGGCACGCTGACTCGTACCTCGAGGTTGCTGAGAGAGGCCGATGGCGTTTTTACTGAAAATTTGCCTCCGGCCATAGAATCTCCTACATGGGGATTTCCTTTGTGGGAGCGGGTTTAC
3-2435133- GGAGAAATTCTCTGGCACGCTGACTCGTACCTCGAGGTTGCTGAGAGAGGCCGATGGCGTTTTTACTGAAAATTTGCCTCCGGCCATAGAATCTCCTACGGGGGCATCCAGGCTGGTCAATCTGTTCTGT
 Consensus GGAGAAATTCTCTGGCACGCTGACTCGTACCTCGAGGTTGCTGAGAGAGGCCGATGGCGTTTTTACTGAAAATTTGCCTCCGGCCATAGAATCTCCTACa..gg.....g.....g.....g.g.....
```
