# Supplementary material for: Bacterial repetitive extragenic palindromic sequences are DNA targets for Insertion Sequence elements
Source: BMC Genomics. 2006 Mar 24;7:62. doi: 10.1186/1471-2164-7-62 (PMC1525189; doi:10.1186/1471-2164-7-62)
Supplement: Additional File 5 — Alignment of DNA sequences from all copies of ISRm22 in Sinorhizobium meliloti and their flanking regions. [file 1471-2164-7-62-S5.pdf]

```
            261                                                    REP frag.  DR        Left End                                          390

1-32440-34  CACCTTCTCCCCGCAGGCGGGGCGAAGGAGACTCGCGGCAAGCGCCAGGGCAATCCCCTCTCCCCGCTCGCGCAGGGCTGTCCGGGGAAAGTTCCGGGTGAATCGAAATGTCGCAGGCGCATGCCCCGTC
5-2292773-  CGCTAACATTGGCATTGGACTAGCGGTCACCCGCCCCTCATCCGCCTGCCGGCACCTTCTCCCCGCAGGCGGCAGGGCTGTCCGGGGAAAGTTCCGGGTGAATCGAAATGTCGCAGGCGCATGCCCCGTC
4’-764961-  TCGGATAGGATTAAAGGAAACATGCAGTAGATTGCCCCTCATCCGCCTGCCGGCACCTTCTCCCCGCAAGCGCAGGGCTGTCCGGGGAAAGTTCCGGGTGAATCGAAATGTCGCAGGCGCATGCCCCGTC
3-169636-1  CCGCCTCTTGCCGGCTGTGCTGGCCCCTCTCCTCGGGTTTAACCCGAGGACTAGCCCTCTCCCCGCAGGCGGCAGGGCTGTCCGGGGAAAGTTCCGGGTGAATCGAAATGTCGCAGGCGCATGCCCCGTC
7-2583816-  TCATGCGGCCATGCTCCTCAGCGTTCTGCCCCTCACCTTTTGCCCCTCACCCTAACCCTCTCCCCGCACGCGCAGGGCTGTCCGGGGAAAGTTCCGGGTGAATCGAAATGTCGCAGGCGCATGCCCCGTC
8-2860199-  CAACTGAACTCTGCATTCACCGCCCGTTCGAGGGAGCGAGCGGGCGCGGCATATCCCTTCTCCCCGCTTGCGCAGGGCTGTCCGGGGAAAGTTCCGGGTGAATCGAAATGTCGCAGGCGCATGCCCCGTC
9’-1557191                     ATACGCTTGCGGCGGCGGATTTGCCCCTCACCCTAGCCCTCTCCCCGCCTGCGCAGGGCTGTCCGGGGAAAGCTCCGGGTGAATCGAAATGTCGCAGGCGCATGCCCCGTC
2’-158601-                        AGACCGGGAAATTCGCCCTGCCCCTCACCCTAACCCTCTCCCCGCTCGCGCAGGGCTGTCCGGGGAAAGTTCCGGGTGAATCGAAATGTCGCAGGCGCATGCCCCGTC
6’-2567032                GTCCGGGCGGCAAGGGGCGGCCGCTCATCCGCCTGCCTGCACCTTCTCCCCGCAAGCGCAGGGCTGTCCGGGGAAAGTTCCGGGTGAATCGAAATGTCGCAGGCGCATGCCCCGTC
 Consensus  .............................g.c...c......Cccc...cc....CC.tctCCCcgc..gcGCAGGGCTGTCCGGGGAAAGTTCCGGGTGAATCGAAATGTCGCAGGCGCATGCCCCGTC

            391                     Left End                           orf                                                            520

1-32440-34  AGACGGCGTTTTTCCGTCTATTTTCTGGTTGTCGAGACTCAGAAAAGGAGACGGAGCATGCGGTTCACCCCTAGCATTTTCGCCCAGCTGCTGAAAGCGATTGATCGCCGCAGCTTTCAGGCGATTGTGG
5-2292773-  AGACGGCGTTTTTCCGTCTATTTTCTGGTTGTCGAGACTCAGAAAAGGAGACGGAGCATGCGGTTCACCCCTAGCATTTTCGCCCAGCTGCTGAAAGCGATTGATCGCCGCAGCTTTCAGGCGATTGTGG
4’-764961-  AGACGGCGTTTTTCCGTCTATTTTCTGGTTGTCGAGACTCAGAAAAGGAGACGGAGCATGCGGTTCACCCCTAGCATTTTCGCCCAGCTGCTGAAAGCGATTGATCGCCGCAGCTTTCAGGCGATTGTGG
3-169636-1  AGACGGCGTTTTTCCGTCTATTTTCTGGTTGTCGAGACTCAGAAAAGGAGACGGAGCATGCGGTTCACCCCTAGCATTTTCGCCCAGCTGCTGAAAGCGATTGATCGCCGCAGCTTTCAGGCGATTGTGG
7-2583816-  AGACGGCGTTTTTCCGTCTATTTTCTGGTTGTCGAGACTCAGAAAAGGAGACGGAGCATGCGGTTCACCCCTAGCATTTTCGCCCAGCTGCTGAAAGCGATTGATCGCCGCAGCTTTCAGGCGATTGTGG
8-2860199-  AGACGGCGTTTTTCCGTCTATTTTCTGGTTGTCGAGACTCAGAAAAGGAGACGGAGCATGCGGTTCACCCCTAGCATTTTCGCCCAGCTGCTGAAAGCGATTGATCGCCGCAGCTTTCAGGCGATTGTGG
9’-1557191  AGACGGCGTTTTTCCGTCTATTTTCTGGTTGTCGAGACTCAGAAAAGGAGACGGAGCATGCGGTTCACCCCTAGCATTTTCGCCCAGCTGCTGAAAGCGATTGATCGCCGCAGCTTTCAGGCGATTGTGG
2’-158601-  AGACGGCGTTTTTCCGTCTATTTTCTGGTTGTCGAGACTCAGAAAAGGAGACGGAGCATGCGGTTCACCCCTAGCATTTTCGCCCAGCTGCTGAAAGCGATTGATCGCCGCAGCTTTCAGGCGATTGTGG
6’-2567032  AGACGGCGTTTTTCCGTCTATTTTCTGGTTGTCGAGACTCAGAAAAGGAGACGGAGCATGCGGTTCACCCCTAGCATTTTCGCCCAGCTGCTGAAAGCGATTGATCGCCGCAGCTTTCAGGCGATTGTGG
 Consensus  AGACGGCGTTTTTCCGTCTATTTTCTGGTTGTCGAGACTCAGAAAAGGAGACGGAGCATGCGGTTCACCCCTAGCATTTTCGCCCAGCTGCTGAAAGCGATTGATCGCCGCAGCTTTCAGGCGATTGTGG

            521                                                    orf                                                            650
1-32440-34  ATCGTCATGCCGGGGATGCCTACGACAAGTGCTTTACCAGCTGGGATCATCTGGTGGCACTGATCTATGCCCAGTTGAGCGCCACGACCAGCCTGCGCGGGTTGGAGGCGAGCTTCAACGCCAACAGTCA
5-2292773-  ATCGTCATGCCGGGGATGCCTACGACAAGTGCTTTACCAGCTGGGATCATCTGGTGGCACTGATCTATGCCCAGTTGAGCGCCACGACCAGCCTGCGCGGGTTGGAGGCGAGCTTCAACGCCAACAGTCA
4’-764961-  ATCGTCATGCCGGGGATGCCTACGACAAGTGCTTTACCAGCTGGGATCATCTGGTGGCACTGATCTATGCCCAGTTGAGCGCCACGACCAGCCTGCGCGGGTTGGAGGCGAGCTTCAACGCCAACAGTCA
3-169636-1  ATCGTCATGCCGGGGATGCCTACGACAAGTGCTTTACCAGCTGGGATCATCTGGTGGCACTGATCTATGCCCAGTTGAGCGCCACGACCAGCCTGCGCGGGTTGGAGGCGAGCTTCAACGCCAACAGTCA
7-2583816-  ATCGTCATGCCGGGGATGCCTACGACAAGTGCTTTACCAGCTGGGATCATCTGGTGGCACTGATCTATGCCCAGTTGAGCGCCACGACCAGCCTGCGCGGGTTGGAGGCGAGCTTCAACGCCAACAGTCA
8-2860199-  ATCGTCATGCCGGGGATGCCTACGACAAGTGCTTTACCAGCTGGGATCATCTGGTGGCACTGATCTATGCCCAGTTGAGCGCCACGACCAGCCTGCGCGGGTTGGAGGCGAGCTTCAACGCCAACAGTCA
9’-1557191  ATCGTCATGCCGGGGATGCCTACGACAAGTGCTTTACCAGCTGGGATCATCTGGTGGCACTGATCTATGCCCAGTTGAGCGCCACGACCAGCCTGCGCGGGTTGGAGGCGAGCTTCAACGCCAACAGTCA
2’-158601-  ATCGTCATGCCGGGGATGCCTACGACAAGTGCTTTACCAGCTGGGATCATCTGGTGGCACTGATCTATGCCCAGTTGAGCGCCACGACCAGCCTGCGCGGGTTGGAGGCGAGCTTCAACGCCAACAGTCA
6’-2567032  ATCGTCATGCCGGGGATGCCTACGACAAGTGCTTTACCAGCTGGGATCATCTGGTGGCACTGATCTATGCCCAGTTGAGCGCCACGACCAGCCTGCGCGGGTTGGAGGCGAGCTTCAACGCCAACAGTCA
 Consensus  ATCGTCATGCCGGGGATGCCTACGACAAGTGCTTTACCAGCTGGGATCATCTGGTGGCACTGATCTATGCCCAGTTGAGCGCCACGACCAGCCTGCGCGGGTTGGAGGCGAGCTTCAACGCCAACAGTCA
```

```
            651                                                           orf                                                          780
1-32440-34  GCATCATTACCATCTCGGCAGTGGCCGGCTGATGCGCTCGACGCTGTCGGACGCCAACCGCCGCCGCCCGGTCGCCGTCTTCGCCGAGACCTTCGCGCTTCTGGCCGGTCAGCTCGACCGGCAGACGCGC
5-2292773-  GCATCATTACCATCTCGGCAGTGGCCGGCTGATGCGCTCGACGCTGTCGGACGCCAACCGCCGCCGCCCGGTCGCCGTCTTCGCCGAGACCTTCGCGCTTCTGGCCGGTCAGCTCGACCGGCAGACGCGC
4'-764961-  GCATCATTACCATCTCGGCAGTGGCCGGCTGATGCGCTCGACGCTGTCGGACGCCAACCGCCGCCGCCCGGTCGCCGTCTTCGCCGAGACCTTCGCGCTTCTGGCCGGTCAGCTCGACCGGCAGACGCGC
3-169636-1  GCATCATTACCATCTCGGCAGTGGCCGGCTGATGCGCTCGACGCTGTCGGACGCCAACCGCCGCCGCCCGGTCGCCGTCTTCGCCGAGACCTTCGCGCTTCTGGCCGGTCAGCTCGACCGGCAGACGCGC
7-2583816-  GCATCATTACCATCTCGGCAGTGGCCGGCTGATGCGCTCGACGCTGTCGGACGCCAACCGCCGCCGCCCGGTCGCCGTCTTCGCCGAGACCTTCGCGCTTCTGGCCGGTCAGCTCGACCGGCAGACGCGC
8-2860199-  GCATCATTACCATCTCGGCAGTGGCCGGCTGATGCGCTCGACGCTGTCGGACGCCAACCGCCGCCGCCCGGTCGCCGTCTTCGCCGAGACCTTCGCGCTTCTGGCCGGTCAGCTCGACCGGCAGACGCGC
9'-1557191  GCATCATTACCATCTCGGCAGTGGCCGGCTGATGCGCTCGACGCTGTCGGACGCCAACCGCCGCCGCCCGGTCGCCGTCTTCGCCGAGACCTTCGCGCTTCTGGCCGGTCAGCTCGACCGGCAGACGCGC
2'-158601-  GCATCATTACCATCTCGGCAGTGGCCGGCTGATGCGCTCGACGCTGTCGGACGCCAACCGCCGCCGCCCGGTCGCCGTCTTCGCCGAGACCTTCGCGCTTCTGGCCGGTCAGCTCGACCGGCAGACGCGC
6'-2567032  GCATCATTACCATCTCGGCAGTGGC------------TCGACGCTGTCGGACGCCAACCGCCGCCGCCCGGTCGCCGTCTTCGCCGAGACCTTCGCGCTTCTGGCCGGTCAGCTCGACCGGCAGACGCGC
 Consensus  GCATCATTACCATCTCGGCAGTGGCCGGCTGATGCGCTCGACGCTGTCGGACGCCAACCGCCGCCGCCCGGTCGCCGTCTTCGCCGAGACCTTCGCGCTTCTGGCCGGTCAGCTCGACCGGCAGACGCGC

            781                                                           orf                                                          910
1-32440-34  CGGGAGGGCACCAAGATGCTGCGGCTGATCGATTCGACCCCGATACCGCTGGGTAAGCTGTACGATTGGGCCAAGTCGAACGGCCGCATCCGCGGCATGAAGCTGCATGTCGTCTATGATCCCAAGGCCG
5-2292773-  CGGGAGGGCACCAAGATGCTGCGGCTGATCGATTCGACCCCGATACCGCTGGGTAAGCTGTACGATTGGGCCAAGTCGAACGGCCGCATCCGCGGCATGAAGCTGCATGTCGTCTATGATCCCAAGGCCG
4'-764961-  CGGGAGGGCACCAAGATGCTGCGGCTGATCGATTCGACCCCGATACCGCTGGGTAAGCTGTACGATTGGGCCAAGTCGAACGGCCGCATCCGCGGCATGAAGCTGCATGTCGTCTATGATCCCAAGGCCG
3-169636-1  CGGGAGGGCACCAAGATGCTGCGGCTGATCGATTCGACCCCGATACCGCTGGGTAAGCTGTACGATTGGGCCAAGTCGAACGGCCGCATCCGCGGCATGAAGCTGCATGTCGTCTATGATCCCAAGGCCG
7-2583816-  CGGGAGGGCACCAAGATGCTGCGGCTGATCGATTCGACCCCGATACCGCTGGGTAAGCTGTACGATTGGGCCAAGTCGAACGGCCGCATCCGCGGCATGAAGCTGCATGTCGTCTATGATCCCAAGGCCG
8-2860199-  CGGGAGGGCACCAAGATGCTGCGGCTGATCGATTCGACCCCGATACCGCTGGGTAAGCTGTACGATTGGGCCAAGTCGAACGGCCGCATCCGCGGCATGAAGCTGCATGTCGTCTATGATCCCAAGGCCG
9'-1557191  CGGGAGGGCACCAAGATGCTGCGGCTGATCGATTCGACCCCGATCCCGCTGGGTAAGCTGTACGATTGGGCCAAGTCGAACGGCCGCATTCGCGGCATGAAGCTGCATGTCGTCTATGATCCCAAGGCCG
2'-158601-  CGGGAGGGCACCAAGATGCTGCGGCTGATCGATTCGACCCCGATACCGCTGGGTAAGCTGTACGATTGGGCCAAGTCGAACGGCCGCATCCGCGGCATGAAGCTGCATGTCGTCTATGATCCCAAGGCCG
6'-2567032  CGGGAGGGCACCAAGATGCTGCGGCTGATCGATTCGACCCCGATACCGCTGGGTAAGCTGTACGATTGGGCCAAGTCGAACGGCCGCATCCGCGGCATGAAGCTGCATGTCGTCTATGATCCCAAGGCCG
 Consensus  CGGGAGGGCACCAAGATGCTGCGGCTGATCGATTCGACCCCGATACCGCTGGGTAAGCTGTACGATTGGGCCAAGTCGAACGGCCGCATCCGCGGCATGAAGCTGCATGTCGTCTATGATCCCAAGGCCG

            911                                                           orf                                                         1040
1-32440-34  ATTGTCCAGGCATCCTCGACATCACCGACGCCAACGTCAACGACGCCCAGATCGGCCGCACGATCACCATCGAAAAGGGCGCAACCTATGTCTTCGACAAGGGCTACTGCCATTACGGCTGGTGGACGGC
5-2292773-  ATTGTCCAGGCATCCTCGACATCACCGACGCCAACGTCAACGACGCCCAGATCGGCCGCACGATCACCATCGAAAAGGGCGCAACCTATGTCTTCGACAAGGGCTACTGCCATTACGGCTGGTGGACGGC
4'-764961-  ATTGTCCAGGCATCCTCGACATCACCGACGCCAACGTCAACGACGCCCAGATCGGCCGCACGATCACCATCGAAAAGGGCGCAACCTATGTCTTCGACAAGGGCTACTGCCATTACGGCTGGTGGACGGC
3-169636-1  ATTGTCCAGGCATCCTCGACATCACCGACGCCAACGTCAACGACGCCCAGATCGGCCGCACGATCACCATCGAAAAGGGCGCAACCTATGTCTTCGACAAGGGCTACTGCCATTACGGCTGGTGGACGGC
7-2583816-  ATTGTCCAGGCATCCTCGACATCACCGACGCCAACGTCAACGACGCCCAGATCGGCCGCACGATCACCATCGAAAAGGGCGCAACCTATGTCTTCGACAAGGGCTACTGCCATTACGGCTGGTGGACGGC
8-2860199-  ATTGTCCAGGCATCCTCGACATCACCGACGCCAACGTCAACGACGCCCAGATCGGCCGCACGATCACCATCGAAAAGGGCGCAACCTATGTCTTCGACAAGGGCTACTGCCATTACGGCTGGTGGACGGC
9'-1557191  ATTGTCCAGGCATCCTCGACATCACCGACGCCAACGTCAACGACGCCCAGATCGGCCGCACGATCACCATCGAAAAGGGCGCAACCTATGTCTTCGACAAGGGCTACTGCCATTACGGCTGGTGGACGGC
2'-158601-  ATTGTCCAGGCATCCTCGACATCACCGACGCCAACGTCAACGACGCCCAGATCGGCCGCACGATCACCATCGAAAAGGGCGCAACCTATGTCTTCGACAAGGGCTACTGCCATTACGGCTGGTGGACGGC
6'-2567032  ATTGTCCAGGCATCCTCGACATCACCGACGCCAACGTCAACGACGCCCAGATCGGCCGCACGATCACCATCGAAAAGGGCGCAACCTATGTCTTCGACAAGGGCTACTGCCATTACGGCTGGTGGACGGC
 Consensus  ATTGTCCAGGCATCCTCGACATCACCGACGCCAACGTCAACGACGCCCAGATCGGCCGCACGATCACCATCGAAAAGGGCGCAACCTATGTCTTCGACAAGGGCTACTGCCATTACGGCTGGTGGACGGC
```

```
            1041                                                             orf                                                             1170
1-32440-34  GATCGCCGAAGCCGGCGCCAGTTTCGTCACCCGGCCGAAGACCAACATGGGACTGGCTTTGGTCGCTGAACGCCCCGTTGAGCAACCTCAAGGCGATGGCTTCCTGGTCCTCGAAGACAGCCAGGTCAGC
5-2292773-  GATCGCCGAAGCCGGCGCCAGTTTCGTCACCCGGCCGAAGACCAACATGGGACTGGCTTTGGTCGCTGAACGCCCCGTTGAGCAACCTCAAGGCGATGGCTTCCTGGTCCTCGAAGACAGCCAGGTCAGC
4’-764961-  GATCGCCGAAGCCGGCGCCAGTTTCGTCACCCGGCCGAAGACCAACATGGGACTGGCTTTGGTCGCTGAACGCCCCGTTGAGCAACCTCAAGGCGATGGCTTCCTGGTCCTCGAAGACAGCCAGGTCAGC
3-169636-1  GATCGCCGAAGCCGGCGCCAGTTTCGTCACCCGGCCGAAGACCAACATGGGACTGGCTTTGGTCGCTGAACGCCCCGTTGAGCAACCTCAAGGCGATGGCTTCCTGGTCCTCGAAGACAGCCAGGTCAGC
7-2583816-  GATCGCCGAAGCCGGCGCCAGTTTCGTCACCCGGCCGAAGACCAACATGGGACTGGCTTTGGTCGCTGAACGCCCCGTTGAGCAACCTCAAGGCGATGGCTTCCTGGTCCTCGAAGACAGCCAGGTCAGC
8-2860199-  GATCGCCGAAGCCGGCGCCAGTTTCGTCACCCGGCCGAAGACCAACATGGGACTGGCTTTGGTCGCTGAACGCCCCGTTGAGCAACCTCAAGGCGATGGCTTCCTGGTCCTCGAAGACAGCCAGGTCAGC
9’-1557191  GATCGCCGAAGCCGGCGCCAGTTTCGTCACTCGGCCGAAGACCAACATGGGACTGGCTTTGGTCGCTGAACGCCCCATCGAGCAACCTCAAGGCGATGGCTTCCTGGTCCTCGAAGACAGCCAGGTCAGC
2’-158601-  GATCGCCGAAGCCGGCGCCAGTTTCGTCACCCGGCCGAAGACCAACATGGGACTGGCTTTGGTCGCTGAACGCCCCGTTGAGCAACCTCAAGGCGATGGCTTCCTGGTCCTCGAAGACAGCCAGGTCAGC
6’-2567032  GATCGCCGAAGCCGGCGCCAGTTTCGTCACCCGGCCGAAGACCAACATGGGACTGGCTTTGGTCGCTGAACGCCCCGTTGAGCAACCTCAAGGCGATGGCTTCCTGGTCCTCGAAGACAGCCAGGTCAGC
 Consensus  GATCGCCGAAGCCGGCGCCAGTTTCGTCACCCGGCCGAAGACCAACATGGGACTGGCTTTGGTCGCTGAACGCCCCGTTGAGCAACCTCAAGGCGATGGCTTCCTGGTCCTCGAAGACAGCCAGGTCAGC
```

```
            1071                                                             orf                                                             1300
1-32440-34  CTCGCCAGCAAGGGCGATTCCAAGCTGCCGATCGGCTTGCGCCGGGTAATCGTCAAGCGCCAGGACGGCGATACGATCACGCTTCTGACCAACGACCTCGAGCGCTCCGCCGTCGAGATCGGCCAGCTCT
5-2292773-  CTCGCCAGCAAGGGCGATTCCAAGCTGCCGATCGGCTTGCGCCGGGTAATCGTCAAGCGCCAGGACGGCGATACGATCACGCTTCTGACCAACGACCTCGAGCGCTCCGCCGTCGAGATCGGCCAGCTCT
4’-764961-  CTCGCCAGCAAGGGCGATTCCAAGCTGCCGATCGGCTTGCGCCGGGTAATCGTCAAGCGCCAGGACGGCGATACGATCACGCTTCTGACCAACGACCTCGAGCGCTCCGCCGTCGAGATCGGCCAGCTCT
3-169636-1  CTCGCCAGCAAGGGCGATTCCAAGCTGCCGATCGGCTTGCGCCGGGTAATCGTCAAGCGCCAGGACGGCGATACGATCACGCTTCTGACCAACGACCTCGAGCGCTCCGCCGTCGAGATCGGCCAGCTCT
7-2583816-  CTCGCCAGCAAGGGCGATTCCAAGCTGCCGATCGGCTTGCGCCGGGTAATCGTCAAGCGCCAGGACGGCGATACGATCACGCTTCTGACCAACGACCTCGAGCGCTCCGCCGTCGAGATCGGCCAGCTCT
8-2860199-  CTCGCCAGCAAGGGCGATTCCAAGCTGCCGATCGGCTTGCGCCGGGTAATCGTCAAGCGCCAGGACGGCGATACGATCACGCTTCTGACCAACGACCTCGAGCGCTCCGCCGTCGAGATCGGCCAGCTCT
9’-1557191  CTCGCCAGCAAGGGCGATTCCAAGCTGCCGATCGGCTTGCGCCGGGTAATCGTCAAGCGCCAGGACGGCGACACGATCACGCTTCTGACCAACGACCTCGAGCGCTCCGCCGTCGAGATCGGCCAGCTCT
2’-158601-  CTCGCCAGCAAGGGCGATTCCAAGCTGCCGATCGGCTTGCGCCGGGTAATCGTCAAGCGCCAGGACGGCGATACGATCACGCTTCTGACCAACGACCTCGAGCGCTCCGCCGTCGAGATCGGCCAGCTCT
6’-2567032  CTCGCCAGCAAGGGCGATTCCAAGCTGCCGATCGGCTTGCGCCGGGTAATCGTCAAGCGCCAGGACGGCGATACGATCACGCTTCTGACCAACGACCTCGAGCGCTCCGCCGTCGAGATCGGCCAGCTCT
 Consensus  CTCGCCAGCAAGGGCGATTCCAAGCTGCCGATCGGCTTGCGCCGGGTAATCGTCAAGCGCCAGGACGGCGATACGATCACGCTTCTGACCAACGACCTCGAGCGCTCCGCCGTCGAGATCGGCCAGCTCT
```

```
            1301                                                             orf                                                             1430
1-32440-34  ATAAGGATCGCTGGCAGATCGAGCTTCTGTTCCGCTGGATCAAACAGCACCTCAAGATCCGCAAGTTCCTCGGCAACAACGACAACGCCATCCGCCTGCAGATCTTCGCCGCGATGATCGCCTATGCACT
5-2292773-  ATAAGGATCGCTGGCAGATCGAGCTTCTGTTCCGCTGGATCAAACAGCACCTCAAGATCCGCAAGTTCCTCGGCAACAACGACAACGCCATCCGCCTGCAGATCTTCGCCGCGATGATCGCCTATGCACT
4’-764961-  ATAAGGATCGCTGGCAGATCGAGCTTCTGTTCCGCTGGATCAAACAGCACCTCAAGATCCGCAAGTTCCTCGGCAACAACGACAACGCCATCCGCCTGCAGATCTTCGCGGCGATGATCGCCTATGCACT
3-169636-1  ATAAGGATCGCTGGCAGATCGAGCTTCTGTTCCGCTGGATCAAACAGCACCTCAAGATCCGCAAGTTCCTCGGCAACAACGACAACGCCATCCGCCTGCAGATCTTCGCCGCGATGATCGCCTATGCACT
7-2583816-  ATAAGGATCGCTGGCAGATCGAGCTTCTGTTCCGCTGGATCAAACAGCACCTCAAGATCCGCAAGTTCCTCGGCAACAACGACAACGCCATCCGCCTGCAGATCTTCGCCGCGATGATCGCCTATGCACT
8-2860199-  ATAAGGATCGCTGGCAGATCGAGCTTCTGTTCCGCTGGATCAAACAGCACCTCAAGATCCGCAAGTTCCTCGGCAACAACGACAACGCCATCCGCCTGCAGATCTTCGCCGCGATGATCGCCTATGCACT
9’-1557191  ATAAGGATCGCTGGCAGATCGAGCTTCTGTTCCGCTGGATCAAACAGCACCTCAAGATCCGCAAGTTCCTCGGCAACAACGACAACGCCATCCGCCTGCAGATTTTCGCGGCGATGATCGCCTATGCACT
2’-158601-  ATAAGGATCGCTGGCAGATCGAGCTTCTGTTCCGCTGGATCAAACAGCACCTCAAGATCCGCAAGTTCCTCGGCAACAACGACAACGCCATCCGCCTGCAGATCTTCGCCGCGATGATCGCCTATGCACT
6’-2567032  ATAAGGATCGCTGGCAGATCGAGCTTCTGTTCCGCTGGATCAAACAGCACCTCAAGATCCGCAAGTTCCTCGGCAACAACGACAACGCCATCCGCCTGCAGATCTTCGCCGCGATGATCGCCTATGCACT
 Consensus  ATAAGGATCGCTGGCAGATCGAGCTTCTGTTCCGCTGGATCAAACAGCACCTCAAGATCCGCAAGTTCCTCGGCAACAACGACAACGCCATCCGCCTGCAGATCTTCGCcGCGATGATCGCCTATGCACT
```

```
             1431                                    orf                                                                                  1560
1-32440-34   GTTGCGCATCGCCGCCCGCCTCGCCCGCGTCCCTCTACCGATCCTGCGCTTCACCGACCTCGTCACCCAGTGCCTGTTCCAGCGCAAAAGCATCGCCGAAATCCACAAGCCGCCGCAGGTCAATCCAAGC
5-2292773-   GTTGCGCATCGCCGCCCGCCTCGCCCGCGTCCCTCTACCGATCCTGCGCTTCACCGACCTCGTCACCCAGTGCCTGTTCCAGCGCAAAAGCATCGCCGAAATCCACAAGCCGCCGCAGGTCAATCCAAGC
4’-764961-   GTTGCGCATCGCCGCCCGCCTCGCCCGCGTCCCTCTACCGATCCTGCGCTTCACCGACCTCGTCACCCAGTGCCTGTTCCAGCGCAAAAGCATCGCCGAAATCCACAAGCCGCCGCAGGTCAATCCAAGC
3-169636-1   GTTGCGCATCGCCGCCCGCCTCGCCCGCGTCCCTCTACCGATCCTGCGCTTCACCGACCTCGTCACCCAGTGCCTGTTCCAGCGCAAAAGCATCGCCGAAATCCACAAGCCGCCGCAGGTCAATCCAAGC
7-2583816-   GTTGCGCATCGCCGCCCGCCTCGCCCGCGTCCCTCTACCGATCCTGCGCTTCACCGACCTCGTCACCCAGTGCCTGTTCCAGCGCAAAAGCATCGCCGAAATCCACAAGCCGCCGCAGGTCAATCCAAGC
8-2860199-   GTTGCGCATCGCCGCCCGCCTCGCCCGCGTCCCTCTACCGATCCTGCGCTTCACCGACCTCGTCACCCAGTGCCTGTTCCAGCGCAAAAGCATCGCCGAAATCCACAAGCCGCCGCAGGTCAATCCAAGC
9’-1557191   GTTGCGCATCGCCGCCCGCCTCGCCCGCGTCCCTCTACCGATCCTGCGCTTCACCGACCTCGTCACCCAGTGCCTGTTCCAGCGCAAAAGCATCGCCGAAATCCACAAGCCGCCGCCGGTCAATCCAAGC
2’-158601-   GTTGCGCATCGCCGCCCGCCTCGCCCGCGTCCCTCTACCGATCCTGCGCTTCACCGACCTCGTCACCCAGTGCCTGTTCCAGCGCAAAAGCATCGCCGAAATCCACAAGCCGCCGCAGGTCAATCCAAGC
6’-2567032   GTTGCGCATCGCCGCCCGCCTCGCCCGCGTCCCTCTACCGATCCTGCGCTTCACCGACCTCGTCACCCAGTGCCTGTTCCAGCGCAAAAGCATCGCCGAAATCCACAAGCCGCCGCAGGTCAATCCAAGC
 Consensus   GTTGCGCATCGCCGCCCGCCTCGCCCGCGTCCCTCTACCGATCCTGCGCTTCACCGACCTCGTCACCCAGTGCCTGTTCCAGCGCAAAAGCATCGCCGAAATCCACAAGCCGCCGCAGGTCAATCCAAGC

             1561                    orf                          Right End            DR        REP frag.                                1690
1-32440-34   CGACCAAAACCCCGGACCATCCCAAACCAAATGGTCTTCCGCTATGCATGAATTTTCCCCGGACAGCCCTGCCGCTCGCGGGGAGAGGGTTAGGGTGAGGGGCAGGTAGCCGGCGACGCACATGGTTCAG
5-2292773-   CGACCAAAACCCCGGACCATCCCAAACCAAATGGTCTTCCGCTATGCATGAATTTTCCCCGGACAGCCCTGCCGCTCGCGGGGCGAAGGGACAAGCGGCGCCGACTGAACCCCGCATTCACCGTCCGCCC
4’-764961-   CGACCAAAACCCCGGACCATCCCAAACCAAATGGTCTTCCGCTATGCATGAATTTTCCCCGGACAGCCCTGCCGCAAGCGGGGCGAAGGAACAAGCGGCGCCGACTGAACCTCGCATTCACCGCCCGCTC
3-169636-1   CGACCAAAACCCCGGACCATCCCAAACCAAATGGTCTTCCGCTATGCATGAATTTTCCCCGGACAGCCCTGCCGCTTGCGGGGAGAGGGGACCTACCGCATGTTTCCTTTAATCCTATCCGATTAAAGGA
7-2583816-   CGACCAAAACCCCGGACCATCCCAAACCAAATGGTCTTCCGCTATGCATGAATTTTCCCCGGACAGCCCTGCCGCAGGCGGGGAGAGGGGACGAGATCCCATGGGCGCGTTGTCGGGTGCGATCGAACAC
8-2860199-   CGACCAAAACCCCGGACCATCCCAAACCAAATGGTCTTCCGCTATGCATGAATTTTCCCCGGACAGCCCTGCCGCTTGCGGGGAGAAGGTGGCGGCAGCCGGATGAGGGGCCGCGCGGACACATCAACCG
9’-1557191   CGACCAAAACCCCGGACCATCCCAAACCAAATGGTCTTCCGCTATACATGAATTTTCCCCGGACAGCCCTGCCGCCTGCGGGGAGAGGGGACTTAAGGGTCTGCGGCTTGTCCCTTCTCCCCGTCGAAAC
2’-158601-   CGACCAAAACCCCGGACCATCCCAAACCAAATGGTCTTCCGCTATGCATGAATTTTCCCCGGACAGCCCTGC-GCAAGCGGGGAGAGGGGACTTAAGCCGCAAAGCGAGCCGCTTATCCCTTCTCCCCGCT
6’-2567032   CGACCAAAACCCCGGACCATCCCAAACCAAATGGTCTTCCGCTATGCATGAATTTTCCCCGGACAGCCCTGCCGCAGGCGGGGAGAGGGGACGAGATCCCATGGGCGCGTTGTCGGGTGCGATCGAACAC
 Consensus   CGACCAAAACCCCGGACCATCCCAAACCAAATGGTCTTCCGCTATGCATGAATTTTCCCCGGACAGCCCTGCCGC..GCGGGGaGAgGG.ac..g...c.............c...tc........c..
```
