# Supplementary material for: Bacterial repetitive extragenic palindromic sequences are DNA targets for Insertion Sequence elements
Source: BMC Genomics. 2006 Mar 24;7:62. doi: 10.1186/1471-2164-7-62 (PMC1525189; doi:10.1186/1471-2164-7-62)
Supplement: Additional File 6 — Alignment of DNA sequences from all copies of ISRm19 in Sinorhizobium meliloti and their flanking regions. [file 1471-2164-7-62-S6.pdf]

```
             1                    REP  DR                            Left End                                                                   130
1-13586-14   AGAAGTCGTCCGCTTTCTCTGCCCCTCATCCGCCTGCCGG--CACCTTGCATAAGAAATGTTGTAAAGGATTCGGTATGCCTTTGGCCGGGCGGCCGCCCGGCCAAAGGCGCCGACCGTCGGATCGCCGG
3-1572828-   TCGGGATGAATAAGCAAATCGCCCCTCATCCGCCTGCCGG--CACCTTGCATAAGAAATGTTGTAAAGGATTCGGTATGCCTTTGGCCGGGCGGCCGCCCGGCCAAAGGCGCCGACCGTCGGATCGCCGG
4-1656415-    AAGCTTAAACGCCAGCATCCCCCCTCATCCGGCCTGCCGGCCACCTTGCATAAGAAATGTTGTAAAGGATTCGGTATGCCTTTGGCCGGGCGGCCGCCCGGCCAAAGGCGCCGACCGTCGGATCGCCGG
2-457899-4   ACGGCGGCGGATCTGCGGCAGCCCCTCATCCGCCTGCCGG--CACCTTGCATAAGAAATGTTGTAAAGGATTCGGTATGCCTTTGGCCGGGCGGCCGCCCGGCCAAAGGCGCCGACCGTCGGATCGCCGG
 Consensus   acaGcttgaacgCt..caccGCCCCTCATCCGCCTGCCGG  CACCTTGCATAAGAAATGTTGTAAAGGATTCGGTATGCCTTTGGCCGGGCGGCCGCCCGGCCAAAGGCGCCGACCGTCGGATCGCCGG

             131                                                     Left End                                                      orf      260
1-13586-14   CACCAGAACCTTTGGGGGTTACGGAGAAGCAGGATCGCGGTCGGTTCCTCATCGTGGCCCGAATGGTTGCTGGAACAACCTGGTTCGTATGCAAGGCAGGGACGACAAGGATGACCGAGATTAGCATGAA
3-1572828-   CACCAGAACCTTTGGGGGTTACGGAGAAGCAGGATCGCGGTCGGTTCCTCATCGTGGCCCGAATGGTTGCTGGAACAACCTGGTTCGTATGCAAGGCAGGGACGACAAGGATGACCGAGATTAGCATGAA
4-1656415-   CACCAGAACCTTTGGGGGTTACGGAGAAGCAGGATCGCGGTCGGTTCCTCATCGTGGCCCGAATGGTTGCTGGAACAACCTGGTTCGTATGCAAGGCAGGGACGACAAGGATGACCGAGATTAGCATGAA
2-457899-4   CACCAGAACCTTTGGGGGTTACGGAGAAGCAGGATCGCGGTCGGTTCCTCATCGTGGCCCGAATGGTTGCTGGAACAACCTGGTTCGTATGCAAGGCAGGGACGACAAGGATGACCGAGATTAGCATGAA
 Consensus   CACCAGAACCTTTGGGGGTTACGGAGAAGCAGGATCGCGGTCGGTTCCTCATCGTGGCCCGAATGGTTGCTGGAACAACCTGGTTCGTATGCAAGGCAGGGACGACAAGGATGACCGAGATTAGCATGAA

             261                                                          orf                                                           390
1-13586-14   AGCGGCCGGTATTGATACCGGGAAGATATGGCTTGATGTCGCCACCTACCCGGTGAGCGACAAACAGAAAGTGCCCAACAACGCCGACGGCTGGCAGACTCTGGCTGACTGGCTCGAGCGGCAGGGGATC
3-1572828-   AGCGGCCGGTATTGATACCGGGAAGATATGGCTTGATGTCGCCACCTACCCGGTGAGCGACAAACAGAAAGTGCCCAACAACGCCGACGGCTGGCAGACTCTGGCTGACTGGCTCGAGCGGCAGGGGATC
4-1656415-   AGCGGCCGGTATTGATACCGGGAAGACATGGCTTGATGTCGCCACCTACCCGGTGAGCGACAAACAGAAAGTGCCCAACAACGCCGACGGCTGGCAGACTCTGGCTGACTGGCTCGAGCGGCAGGGGATC
2-457899-4   AGCGGCCGGTATTGATACCGGGAAGACATGGCTTGATGTCGCCACCTACCCGGTGAGCGACAAACAGAAAGTGCCCAACAACGCCGACGGCTGGCAGACTCTGGCTGACTGGCTCGAGCGGCAGGGGATC
 Consensus   AGCGGCCGGTATTGATACCGGGAAGAcATGGCTTGATGTCGCCACCTACCCGGTGAGCGACAAACAGAAAGTGCCCAACAACGCCGACGGCTGGCAGACTCTGGCTGACTGGCTCGAGCGGCAGGGGATC

             391                                                          orf                                                           520
1-13586-14   GGACGGGTCGGGATCGAAGCGTCCGGCGGATATGAGCGCGACGTGATTGCGTATCTGCATCAGAGAGGCTTTGAGGTCGTCCTGCTGCAGCCGCGGCAAGTGCGCGCTTTCGGCCTCTATAAGCTGCGCC
3-1572828-   GGACGGGTCGGGATCGAAGCGTCCGGCGGATATGAGCGCGACGTGATTGCGTATCTGCATCAGAGAGGCTTTGAGGTCGTCCTGCTGCAGCCGCGGCAAGTGCGCGCTTTCGGCCTCTATAAGCTGCGCC
4-1656415-   GGACGGGTCGGGATCGAAGCCTCCGGCGGATATGAGCGCGACGTGATTGCGTATCTGCATCGGAGAGGCTTTGAGGTCGTCCTGCTGCAGCCGCGGCAAGTGCGCGCTTTCGGCCTCTATAAGCTGCGCC
2-457899-4   GGACGGGTCGGGATCGAAGCCTCCGGCGGATATGAGCGCGACGTGATTGCGTATCTGCATCAGAGAGGCTTTGAGGTCGTCCTGCTGCAGCCGCGGCAAGTGCGCGCTTTCGGCCTCTATAAGCTGCGCC
 Consensus   GGACGGGTCGGGATCGAAGCcTCCGGCGGATATGAGCGCGACGTGATTGCGTATCTGCATCAGAGAGGCTTTGAGGTCGTCCTGCTGCAGCCGCGGCAAGTGCGCGCTTTCGGCCTCTATAAGCTGCGCC

             521                                                          orf                                                           650
1-13586-14   GAGCCAAGAACGACCAGCTCGACGCCGCGCTGATCGCCGAGTGCGCTGCCCGTTCGGATGCGCGATGCCATGCGCCGGATAGCCGGCTGATCGCGTTTGGCGAATGGCTGCTGTTCATCGAGCAGATCGA
3-1572828-   GAGCCAAGAACGACCAGCTCGACGCCGCGCTGATCGCCGAGTGCGCTGCCCGTTCGGATGCGCGATGCCATGCGCCGGATAGCCGGCTGATCGCGTTTGGCGAATGGCTGCTGTTCATCGAGCAGATCGA
4-1656415-   GAGCCAAGAACGACCAGCTCGACGCCGCGCTGATCGCCGAGTGCGCTGCCCGTTCGGATGCGCGATGCCATGCGCCGGATAGCCGGCTGATCGCGTTTGGCGAATGGCTGCTGTTCATCGAGCAGATCGA
2-457899-4   GAGCCAAGAACGACCAGCTCGACGCCGCGCTGATCGCCGAGTGCGCTGCCCGTTCGGATGCG-----------------------GCTGATCGCGTTTGGCGAATGGCTGCTGTTCATCGAGCAGATCGA
 Consensus   GAGCCAAGAACGACCAGCTCGACGCCGCGCTGATCGCCGAGTGCGCTGCCCGTTCGGATGCGCGATGCCATGCGCCGGATAGCCGGCTGATCGCGTTTGGCGAATGGCTGCTGTTCATCGAGCAGATCGA

             651                                                          orf                                                           780
1-13586-14   AGCCGATATAGCCTGCCTCAAGACCCGCCGCGAGCGTTTCACCGACAGGTGGATCCTCGAGGAGATCGATCGTTCCATCGGTGAGCTCAAGAGCCGTTGCAAAGCCCAGCTCGCGCTGTTGCAGGCCGCT
3-1572828-   AGCCGATATAGCCTGCCTCAAGACCCGCCGCGAGCGTTTCACCGACAGGTGGATCCTCGAGGAGATCGATCGTTCCATCGGTGAGCTCAAGAGCCGTTGCAAAGCCCAGCTCGCGCTGTTGCAGGCCGCT
4-1656415-   AGCCGATATAGCCTGCCTCAAGACCCGCCGCGAGCGTTTCACCGACAGGCGGATCCTCGAGGAGATCGATCGTTCCATCGGTGAGCTCAAGAGCCGTTGCAAAGCCCAGCTCGCGCTGTTGCAGGCCGCT
2-457899-4   AGCCGATATAGCCTGCCTCAAGACCCGCCGCGAGCGTTTCACCGACAGGCGGATCCTCGAGGAGATCGATCGTTCCATCGGTGAGCTCAAGAGCCGTTGCAAAGCCCAGCTCGCGCTGTTGCAGGCCGCT
 Consensus   AGCCGATATAGCCTGCCTCAAGACCCGCCGCGAGCGTTTCACCGACAGGcGGATCCTCGAGGAGATCGATCGTTCCATCGGTGAGCTCAAGAGCCGTTGCAAAGCCCAGCTCGCGCTGTTGCAGGCCGCT

             781                                                            orf                                                         910
1-13586-14   GTCCGCGAGCATGACGATCTCGCCCGGAAGCTTGATCTGATCGAAAGCATCGACGGCATCGGCATCCGTACGGCACTCACCCTGGTCATTCTGATGCCCGAACTCGGCAGGGTCGACCGTGAGGAGATCG
3-1572828-   GTCCGCGAGCATGACGATCTCGCCCGGAAGCTTGATCTGATCGAAAGCATCGACGGCATCGGCATCCGTACGGCACTCACCCTGGTCATTCTGATGCCCGAACTCGGCAGGGTCGACCGTGAGGAGATCG
```

```
4-1656415-  GTCCGCGAGCATGACGATCTCGCCCGGAAGCTTGATCTGATCGAAAGCATCGACGGCATCGGCATCCGTACGGCACTCACCCTGGTCATTCTGATGCCCGAACTCGGCAGGGTCGACCGTGAGGAGATCG
2-457899-4  GTCCGCGAGCATGACGATCTCGCCCGGAAGCTTGATCTGATCGAAAGCATCGACGGCATCGGCATCCGTACGGCACTCACCCTGGTCATTCTGATGCCCGAACTCGGCAGGGTCGACCGTGAGGAGATCG
 Consensus  GTCCGCGAGCATGACGATCTCGCCCGGAAGCTTGATCTGATCGAAAGCATCGACGGCATCGGCATCCGTACGGCACTCACCCTGGTCATTCTGATGCCCGAACTCGGCAGGGTCGACCGTGAGGAGATCG

            911                                                         orf                                                         1040
1-13586-14  CCGCGCTGACCGGCGTGGCGCCTTATGACGACCAGAGCGGCAAGCGTGAAGGCGAGCGCCATATCGCCGGCGGACGGGCGCGCGTCCGCAGGGCCCTGTTCAATGCCGCCTTACCGGCGTCGCAGCGCTG
3-1572828-  CCGCGCTGACCGGCGTGGCGCCTTATGACGACCAGAGCGGCAAGCGTGAAGGCGAGCGCCATATCGCCGGCGGACGGGCGCGCGTCCGCAGGGCCCTGTTCAATGCCGCCTTACCGGCGTCGCAGCGCTG
4-1656415-  CCGCGCTGACCGGCGTGGCGCCTTATGACGACCAGAGCGGCAAGCGTGAAGGCGAGCGCCATATCGCCGGCGGACGGGCGCGCGTCCGCAGGGCCCTGTTCAATGCCGCCTTACCGGCGTCGCAGCGCTG
2-457899-4  CCGCGCTGACCGGCGTGGCGCCTTATGACGACCAGAGCGGCAAGCGTGAAGGCGAGCGCCATATCGCCGGCGGACGGGCGCGCGTCCGCAGGGCCCTGTTCAATGCCGCCTTACCGGCGTCGCAGCGCTG
 Consensus  CCGCGCTGACCGGCGTGGCGCCTTATGACGACCAGAGCGGCAAGCGTGAAGGCGAGCGCCATATCGCCGGCGGACGGGCGCGCGTCCGCAGGGCCCTGTTCAATGCCGCCTTACCGGCGTCGCAGCGCTG

            1041                                                        orf                                                         1170
1-13586-14  GAACGAGACGCTGGTTGAGCTCTACGATCGTCTCACCGCAAAAGGCAAATCCCACAAAGCGGCGCTCATCGCCTGCGTCCGCAAACTCATCATCTTCGCCAACACCGTCGTCAAGCGCCAAACCCCATGG
3-1572828-  GAACGAGACGCTGGTTGAGCTCTACGATCGTCTCACCGCAAAAGGCAAATCCCACAAAGCGGCGCTCATCGCCTGCGTCCGCAAACTCATCATCTTCGCCAACACCGTCGTCAAGCGCCAAACCCCATGG
4-1656415-  GAACGAGACGCTGGTTGAGCTCTACGACCGTCTCACCGCAAAAGGCAAATCCCACAAAGCGGCGCTCATCGCCTGCGTCCGCAAACTCATCATCTTCGCCAACACCGTCGTCAAGCGCCAAACCCCATGG
2-457899-4  GAACGAGACGCTGGTTGAGCTCTACGACCGTCTCACCGCAAAAGGCAAATCCCACAAAGCGGCGCTCATCGCCTGCGTCCGCAAACTCATCATCTTCGCCAACACCGTCGTCAAGCGCCAAACCCCATGG
 Consensus  GAACGAGACGCTGGTTGAGCTCTACGAcCGTCTCACCGCAAAAGGCAAATCCCACAAAGCGGCGCTCATCGCCTGCGTCCGCAAACTCATCATCTTCGCCAACACCGTCGTCAAGCGCCAAACCCCATGG

            1171             orf                  Right End    DR        REP                      1267
1-13586-14  ACGAAAAGCGCGCCACAACAAAATTCTTGCGCTTAGTCTAATGGTTGCGCTTCTCCCCGCCTGCGGGGAGAAGGGA-TATGCCGCACCGCTCCAGTC
3-1572828-  ACGAAAAGCGCGCCACAACAAAATTCTTGCGCTTAGTCTAATGGTTGCGCTTCTCCCCGCAAGCGGGGAGAAGGGAATTTGCCGTAACGCCTCTTC
4-1656415-  ACGAAAAGCGCGCCACAACAAAATTCTTGCGCTTAGTCTAATGGTTGCGCTTCTCCCCGCAGGCGGGGCGCAGGGGACTCGCGGCAGCGTCGTAC
2-457899-4  ACGAAAAGCGCGCCACAACAAAATTCTTGCGCTTAGTCTAATGGACGCGCTTCTCCCCGCAAGCGGGGAGAAGGGA-CAAGCGGCACGAATCGAGCG
 Consensus  ACGAAAAGCGCGCCACAACAAAATTCTTGCGCTTAGTCTAATGGTTGCGCTTCTCCCCGCAaGCGGGGAGAAGGGA.catGCcGCAcCGccccAgc.
```
