# Supplementary material for: Bacterial repetitive extragenic palindromic sequences are DNA targets for Insertion Sequence elements
Source: BMC Genomics. 2006 Mar 24;7:62. doi: 10.1186/1471-2164-7-62 (PMC1525189; doi:10.1186/1471-2164-7-62)
Supplement: Additional File 7 — Positions of all ISPsy8 copies with adjacent genes and REP sequences. [file 1471-2164-7-62-S7.pdf]

Copy 1

145643..146884 - 413 28850595 - PSPTO0121 - - membrane protein, putative

REP 83 Position: 146973 - 147023 /E=1e-11/ +
GGACGCAGAGCGTCCAGAACGGCATGCGACGCAGAGCGTCGCACGATCGTT

REP 133 Position: 147134 - 147184 /E=8e-10/ +
GGACGCAGAGCGTCCGGAACGGCATGCGACGCGGAGCGTCGCACGATCGTT

REP 341 Position: 147213 - 147263 /E=0.11/ -
TGACGCAGAGCGTCACCCACGGCATTCCTACGCTGGAGCGTGAGGAACGAT

REP 132 Position: 147295 - 147345 /E=8e-10/ +
GGACGCAGAGCGTCCGGAACGGCATGCGACGCGGAGCGTCGCACGATCGTT

147445..147960 + 171 28850597 - PSPTO0123 - - ISPsy8, transposase OrfA

148017..148796 + 259 28850598 - PSPTO0124 - - ISPsy8, transposase OrfB

REP 340 Position: 148809 - 148859 /E=0.11/ -
TGACGCAGAGCGTCACCCACGGCATTCCTACGCTGTGGACTGCCCCCCGGA

REP 154 Position: 148891 - 148941 /E=7e-09/ +
GGACGCAGAGCGTCCGGAACAGCATGCGACGCGGAGCGTCGCACGATCGTT

148983..150377 - 464 28850599 argH PSPTO0125 - - argininosuccinate lyase

---

Copy 2

492163..492777 + 204 28850913 - PSPTO0448 - - glutathione S-transferase, putative

REP 36 Position: 492861 - 492911 /E=1e-13/ +
GGACGCGGAGCGTCCAGAACGGCATGCGACGCAGAGCGTCGCACGATAGTT

493002..493517 + 171 28850914 - PSPTO0449 - - ISPsy8, transposase OrfA
493574..494353 + 259 28850915 - PSPTO0450 - - ISPsy8, transposase OrfB

REP 280 Position: 494366 - 494416 /E=0.001/ -
TGACGCGGAGCGTCACGCAAGGCATTCCCACGCAGTGGACTGCCCCCCGGA

494461..494964 - 167 28850916 - PSPTO0451 - - conserved hypothetical protein

---

Copy 3

561106..563445	-	779	28850978	pqqF	PSPTO0514	-	-	coenzyme PQQ synthesis protein F
563660..564175	+	171	28850979	-	PSPTO0515	-	-	ISPsy8, transposase OrfA
564232..565011	+	259	28850980	-	PSPTO0516	-	-	ISPsy8, transposase OrfB
565108..565914	-	268	28850981	-	PSPTO0517	-	-	hydrolase, carbon-nitrogen family

---

Copy 4

5768201..5768986	+	261	28855431	-	PSPTO5064	-	-	hydroxypyruvate isomerase, putative
5769093..5769608	+	171	28855432	-	PSPTO5065	-	-	ISPsy8, transposase OrfA
5769665..5770444	+	259	28855433	-	PSPTO5066	-	-	ISPsy8, transposase OrfB

REP 39 Position: 5770624 - 5770674 /E=1e-12/ -
CGGACGCGGAGCGTCCAGAACGGCATGCGACGCGGAGCGTCGCACGATAGT

5770690..5771085	-	131	28855434	-	PSPTO5067	-	-	4-hydroxybenzoyl-CoA thioesterase domain protein

---

Copy 5

6272897..6273565	+	222	28855868	-	PSPTO5507	-	-	acetyltransferase, GNAT family

REP 323 Position: 6273655 - 6273705 /E=0.11/ +
CGACGCAGAGCGTCTATAGCTGCATTCCCACGCGGGAGCGTAGGGAACGAT

REP 4 Position: 6273752 - 6273802 /E=2e-15/ -
GGACGCGGAGCGTCCGGAACGGCATGACGACGCAGAGCGTCGCACGATAGT

REP 322 Position: 6273809 - 6273859 /E=0.11/ +
CGACTCAGAGCGTCGAGAGCTGCATTCCCACGCGGTGGACTGCCCCCAAGA

6273896..6274411	+	171	28855869	-	PSPTO5508	-	-	ISPsy8, transposase OrfA
6274468..6275247	+	259	28855870	-	PSPTO5509	-	-	ISPsy8, transposase OrfB

REP 3 Position: 6275342 - 6275392 /E=2e-15/ -

GGACGCGGAGCGTCCGGAACGGCATGACGACGCAGAGCGTCGCACGATAGT

REP 281   Position: 6275399 - 6275449 /E=0.004/ +
CGACGCAGAGCGTCGAGAGCTGCATTCCCACGCGGGGCCGTAGGAACGATA

6275563..6277371	-	602	28855871	oadA	PSPTO5510	-	-	oxaloacetate decarboxylase, alpha subunit

---
