# Supplementary material for: Bacterial repetitive extragenic palindromic sequences are DNA targets for Insertion Sequence elements
Source: BMC Genomics. 2006 Mar 24;7:62. doi: 10.1186/1471-2164-7-62 (PMC1525189; doi:10.1186/1471-2164-7-62)
Supplement: Additional File 8 — Alignment of each reconstructed REP sequence with REP sequences with its same orientation and cluster. [file 1471-2164-7-62-S8.pdf]

Copy 1

```
  REP_340 (reconstructed) TGACGCAGAGCGTCACCCACGGCATTCCTACGCTGGAGCGTGAGGAACGA
  REP_341                 TGACGCAGAGCGTCACCCACGGCATTCCTACGCTGGAGCGTGAGGAACGA
```

Copy 5

```
  REP-322 (reconstructed) CGACTCAGAGCGTCGAGAGCTGCATTCCCACGCGGGGGGCGTAGGGAACG
  REP-323                 CGACGCAGAGCGTCTATAGCTGCATTCCCACGCGGGAG-CGTAGGGAACG
  REP-281                 CGACGCAGAGCGTCGAGAGCTGCATTCCCACGCGGGGC-CGTAGGAACGA
```
