# Supplementary material for: Bacterial repetitive extragenic palindromic sequences are DNA targets for Insertion Sequence elements
Source: BMC Genomics. 2006 Mar 24;7:62. doi: 10.1186/1471-2164-7-62 (PMC1525189; doi:10.1186/1471-2164-7-62)
Supplement: Additional File 9 — Alignment of DNA sequences from all copies of ISPsy7 in Pseudomonas syringae DC3000 and their flanking regions. [file 1471-2164-7-62-S9.pdf]

```
             1                                                                                                                            130
is7c-2.txt          GACTGAAGGTTTGCGTCCCCTTCGTCTAGTGGCCTAGGACACCGCCCTTTCACGGCGGTAACAGGGGTTCGAGTCCCCTAGGGGACGCCACGATTTCCCGCTCTGCGGGACCAAGGGTCATTC
is7c-3.txt                TTCAAGCGTAGGGGTGCCAGACATGCGTTTGATCTCCATTGTTATTGTATTGAGACGTCGGTCGCTTGTGGATAAGATAAGACGTCGTACAACGGTCGTGGAGATTCAAATGTAACT
CI-is7c-5.           ACAGCAGCGAGTGCTGGTCATGACCGACGGCAGAATCAAACAACTGCCAACGCTGCCTGCCTTCAACTGCGCAAGCCTGCTGATCGACATCGAAAAAGGCCCGATCCGCTTGGGAAGGGCTC
CI-is7c-6.   TGGAATTCGGTAAAACCATGCAACACCTGCGCGAAGTGCCCTTGGA-ACAAACCACCCTGAAACGCACCATCGGCGTCACCCTGCGCAAGGGCGGCTACCTGTCGCCCGCCGCAGAAGGCATGTTGCAAA
CI-is7c-8.      AACCAGCATCACGCTTACCTGAGCGAAAACATCAGCACATTCGACCCCACCACCCGCCACGCCCTCGACAGTCT----------TGGCGGCGCCAGCCCACAGGCCTACGCGCAACTGGAGCGCACC
CI-is7c-9.        TTCATCGCCTCGATCATCGGCGTCATGCTGCTGGCCGTGGGTGCCGTGCT--GTGCGGCGTGGTCACGGCTGCGCTGCTGTCGGAAACCTTCCCGACCCGCACCCGCTACACCGCCTCGGCGATC
 Consensus      ..........a.c.at....g..ct....c...tGc.c.tg....Cc...c.....g....g...C..CgG...c.......t..gg..cg.C..c....c.cgC.C......a..........a.c

             131                                                                                                                          260
is7c-2.txt   GATTATTGAATGGCCCTTTTGTTTTTCCGGGGTTTGAAAACCCTTTCTTTCTGATTCTTATTCT------TCATACATAACCCTGACTAAGCCTCGTGACTAAGTCTCGTGCCAGTGCGTTTGCACGCAT
is7c-3.txt   GTGTGTAAAAGCGTTTTACGGATCGGGTGAGGTGTTGGCGTCCGTTGTTATTGCACAATGATTACCCGACTGATTTTCGTCCCAATGCACGCGTAGGCACGCGTTGGGTGGCGCTCTGCGTCACACACGT
CI-is7c-5.   GGGAGCTGGCTGC-------AAGCCTGGGTGCTGATTACCGGCATATTGATGAGCTGAAGCTGGTTTGAAGGGGGTTTTTCACTGTCGTGGCCATGTGTTGGCATTCACCCGTGACACTATGCGCGCACG
CI-is7c-6.   TGCTGCGCGACAACGGCGGGGACTTTCTGGGCTGGGGGTGAGCATGCCTTGCCAGCATCCGTCTGGCGATGCCTGTGACTCTCATATCAATGCTTGCGTTGCATCCAGTTCGTGACGCT-----------
CI-is7c-8.   CTCAACGCCCAGGCCTACATGATGTCCACGGTGGACTACTTCTACCTGCTGGGCTGGATATTCGCGGGCCTGATCCTGCTGGTCTGGCTGGCCAAACCCCCATTCGGCGCGAAGGCCGGGCCGGCGGCGG
CI-is7c-9.   ACCTACAACATGGCCTACACCCTGTTCGGCGGCACCGCACCGCTGATGGCAACCTGGCTGATCAGCGTCACCGGCAGCAACCTGTCGCCAGCGTTCTACCTGATTGCCGTAGCCGTGCTAGCAATGGCAG
 Consensus   ...t.c...a.ggc......gat.ttc.G.G.tg........C.t.............t..Tc....g.....t.....tC.c.....c..GCct.g...c...tt..........c.c.........c..

             261                                                                                                                          390
is7c-2.txt   CAGGTGGCACCCCTCATCGCATCTACGCCGCAGCACGACGTCATGCCTTGCCGGTAATCACTGCACATAAACGAAAGCGCTTTTTCATCGCGATATCACTGACGACGCAGAGTGCGACGTAAGTGACGGC
is7c-3.txt   GCGCCGCACACTCAAGATCGGACGCAGAGCGTCCAGAACGGCATGCGACGCGGAGCGTCGCACGATAGTTGAAAAAGCGCTTTTTGATCACGATGGCACTGACGACGCCGATTGCGACGTAAGTGACGGC
CI-is7c-5.   GCGCACGATAGTTGTATCTTTGCCGCAGCGCAGCACAACGTCATGCCTGGCCCGCAGTCACTGCCTATAAACAAAAGCGCTTTTTTACCGCGATGGCACTGACGACGCAGAGTGCGACTTAAGTGACGGC
CI-is7c-6.   ---CCGCGTCAACGCGTCAGATTCTTGCTACAGCGCAACGTCATGCCTTATCCGGAATCACTGCACATAAACAAAAGCGCTTTTTTATCGCAATGGCACTGACGACGCAGAGTGCGACGTAAGTGACGGC
CI-is7c-8.   GGGGCGGGCATTGAGAAGGGTTTCTCGTGCCAATGGGGTCGCATGC--AGTTGTGACTGCTTTAACTGCTTTAGATCTGCTTTTCGATCACGATGGCACTGACGACGCAGATTGCGACGTAAGTGACGGC
CI-is7c-9.   GCGGCCTGGCATTGCCCGAGACCTCGAAGATCTCGCTGCATGACGTGCCGGTTGCGGGCAAAGGGGCAGTGACGGGTTCGATGGCGTGAGGGTCTCGGGGTCACGCTTTGCGTCAAATATGTGCTGCAGC
 Consensus   g.G.cg.....t.......g..c...g...ca.C.c.aCgtCATGC...G...g.a.TCactg.a.a.....aaAagcGCTTTTt.AtCgCGATggCACTGACGACGCaGAgTGCGACgTAAGTGACGGC

             391                                                                                                                          520
is7c-2.txt   TGAGCCCTGACACTGATCCGGGGGTAGCTCGGCA-GGACGCCGAGCTAGCCGCACCGTGCCAGGGATGGCACGTTGCGGCGACCCCCGGATCAGTGTCAGGGCGAAGGAACCCGACGAAGTCGGGCCGGA
is7c-3.txt   TGAGCCCTGACACTGATCCGGGGGATGCGAGGCA-GGACGCCGAGCAAGCTGCACCGGGCCAAGGATGGCCCGTTGCAGCGACCGCCGGATCAGTGTCAGGGCGAAGGAACCCGACGAAGTCGGGCCGGA
CI-is7c-5.   TGAGTCCTGGCGCTGATCCGGGGGATGCGAGGCA-GGACGCCGAGCAAGCCGCACCGGGCCAAGGATGGCCCGTTGCGGCGACCCCCGGATCAGCGTCAGGGCGAAGGAACCCGACGAAGTCGGGCCGGA
CI-is7c-6.   TGAGCCCTGGCGCTGATCCGGGGGATGCGAGGCA-GGACGCCGAGCAAGCTGCACCGGGCCAAGGATGGCCCGTTGCAGCGACCCCCGGATCAGTGTCAGGGCGAAGGAACCCGACGAAGTCGGGCCGGA
CI-is7c-8.   TGAGCCCTGACACTGATCCGGGGGGAACTCGGCAAGGATGCCGAGTTAGCTGCACCGGGCCAAGGATGGCCCGTTGCAGCGACCCCCGGATCAGTGTCAGGGCGAAGGAACCCGACGAAGTCGGGCCGGA
CI-is7c-9.   ACAACGCCATACCTTGCCCGTAGCCACTGCACATAAACGAAAGCTTTTTCATCACGGTGGCACTGACGACGCA-------GAGTGCGACGTCAGTGTCAGGGCAAAGGAACCCGACGAAGTCGGGCCGGA
 Consensus   TGAGCCCTG.C.CTGATCCGGGGG..gCg.GGCA.GGAcGCCGAGc.AGC.GCACCGgGCCAaGGATGGCcCGTTGC.GCGACCcCCGGATCAGTGTCAGGGCGAAGGAACCCGACGAAGTCGGGCCGGA

             521                                                                                                                          650
is7c-2.txt   AACGGAGCTGGGA--TAATGAGATGGTCAGCCCGATCCGAGAAACCCGGAGTGTCTACACTTTCCGGGTTTCTCTCTAACGTCGAGGAGATCACTCATGAAAAACGGCCTCAAGCTCTTAGGCATCGACA
is7c-3.txt   AACGGAGCTGGGA--TAATGAGATGGTCAGCCCGATCCGAGAAACCCGGAGTGTCTACACTTTCCGGGTTTCTCTCTAACGTCGAGGAGATCACTCATGAAAAACGGCCTCAAGCTCTTAGGCATCGACA
CI-is7c-5.   AACGGAGCTGCGGGCTAATGAGATGGTCAGCCCGATCCGAGAAACCCGGAGTGTCTACACTTTCCGGGTTTCTCTCTAACGTCGAGGAGATCACTCATGAAAAACGGCCTCAAGCTCTTAGGCATCGACA
CI-is7c-6.   AACGGAGCTGCGGGCTAATGAGATGGTCAGCCCGATCCGAGAAACCCGGAGTGTCTACACTTTCCGGGTTTCTCTCTAACGTCGAGGAGATCACTCATGAAAAACGGCCTCAAGCTCTTAGGCATCGACA
CI-is7c-8.   AACGGAGCCAGGGG-TAATGAGATGGTCAGCCCGATCCGAGAAACCCGGAGTGTCTACACTTTCCGGGTTTCTCTCTAACGTCGAGGAGATCACTCATGAAAAACGGCCTCAAGCTCTTAGGCATCGACA
CI-is7c-9.   AACGGAGCGGTGA--TAATGAGATGGTCAGCCCGATCCGAGAAACCCGGAGTGTCTACACTTTCCGGGTTTCTCTCTAACGTCGAGGAGATCACTCATGAAAAACGGCCTCAAGCTCTTAGGCATCGACA
 Consensus   AACGGAGCtG.G...TAATGAGATGGTCAGCCCGATCCGAGAAACCCGGAGTGTCTACACTTTCCGGGTTTCTCTCTAACGTCGAGGAGATCACTCATGAAAAACGGCCTCAAGCTCTTAGGCATCGACA
```

```
             651                                                                                                                            780
is7c-2.txt   TTGGTAAAAACAGTTTTCACCTCATAGGCCATGATGCAGGCGGCCACCAAGTATTACGCAAGTGCTTCAATCGCAATCGCCTTCTGCAGTTTGCGGCTCAGATTGAACCCTGCACCATTGCAATGGAGTC
is7c-3.txt   TTGGTAAAAACAGTTTTCACCTCATAGGCCATGATGCAGGCGGCCACCAAGTATTACGCAAGTGCTTCAATCGCAATCGCCTTCTGCAGTTTGCGGCTCAGATTGAACCCTGCACCATTGCAATGGAGTC
CI-is7c-5.   TTGGTAAAAACAGTTTTCACCTCATAGGCCATGATGCAGGCGGCCACCAAGTATTACGCAAGTGCTTCAATCGCAATCGCCTTCTGCAGTTTGCGGCTCAGATTGAACCCTGCACCATTGCAATGGAGTC
CI-is7c-6.   TTGGTAAAAACAGTTTTCACCTCATAGGCCATGATGCAGGCGGCCACCAAGTATTACGCAAGTGCTTCAATCGCAATCGCCTTCTGCAGTTTGCGGCTCAGATTGAACCCTGCACCATTGCAATGGAGTC
CI-is7c-8.   TTGGTAAAAACAGTTTTCACCTCATAGGCCATGATGCAGGCGGCCACCAAGTATTACGCAAGTGCTTCAATCGCAATCGCCTTCTGCAGTTTGCGGCTCAGATTGAACCCTGCACCATTGCAATGGAGTC
CI-is7c-9.   TTGGTAAAAACAGTTTTCACCTCATAGGCCATGATGCAGGCGGCCACCAAGTATTACGCAAGTGCTTCAATCGCAATCGCCTTCTGCAGTTTGCGGCTCAGATTGAACCCTGCACCATTGCAATGGAGTC
 Consensus   TTGGTAAAAACAGTTTTCACCTCATAGGCCATGATGCAGGCGGCCACCAAGTATTACGCAAGTGCTTCAATCGCAATCGCCTTCTGCAGTTTGCGGCTCAGATTGAACCCTGCACCATTGCAATGGAGTC

             781                                                                                                                            910
is7c-2.txt   GTGCGGTGGCTCTCACTGGCTTGCCAGGAAACTCGCCTCGCTGGGGCACCGGGTGAAGTTGATTGCTCCGCAACATGTGAAGCCCTACGTGACGGGCAACAAGAATGACTTCATCGATGCCGAAGCTATA
is7c-3.txt   GTGCGGTGGCTCTCACTGGCTTGCCAGGAAACTCGCCTCGCTGGGGCACCGGGTGAAGTTGATTGCTCCGCAACATGTGAAGCCCTACGTGACGGGCAACAAGAATGACTTCATCGATGCCGAAGCTATA
CI-is7c-5.   GTGCGGTGGCTCTCACTGGCTTGCCAGGAAACTCGCCTCGCTGGGGCACCGGGTGAAGTTGATTGCTCCGCAACATGTGAAGCCCTACGTGACGGGCAACAAGAATGACTTCATCGATGCCGAAGCTATA
CI-is7c-6.   GTGCGGTGGCTCTCACTGGCTTGCCAGGAAACTCGCCTCGCTGGGGCACCGGGTGAAGTTGATTGCTCCGCAACATGTGAAGCCCTACGTGACGGGCAACAAGAATGACTTCATCGATGCCGAAGCTATA
CI-is7c-8.   GTGCGGTGGCTCTCACTGGCTTGCCAGGAAACTCGCCTCGCTGGGGCACCGGGTGAAGTTGATTGCTCCGCAACATGTGAAGCCCTACGTGACGGGCAACAAGAATGACTTCATCGATGCCGAAGCTATA
CI-is7c-9.   GTGCGGTGGCTCTCACTGGCTTGCCAGGAAACTCGCCTCGCTGGGGCACCGGGTGAAGTTGATTGCTCCGCAACATGTGAAGCCCTACGTGACGGGCAACAAGAATGACTTCATCGATGCCGAAGCTATA
 Consensus   GTGCGGTGGCTCTCACTGGCTTGCCAGGAAACTCGCCTCGCTGGGGCACCGGGTGAAGTTGATTGCTCCGCAACATGTGAAGCCCTACGTGACGGGCAACAAGAATGACTTCATCGATGCCGAAGCTATA

             911                                                                                                                           1040
is7c-2.txt   TGCGAGGCGGCCTCCAGACCACGTACTCATTACGTTGCCGTCAAGACTGTGGAGCAGCAGGTACTTTCTGTACAACATCGTCTTCGCGAGTCACTGGTTTGCCACCGTACTGAAGCCATCAACAGGGTGC
is7c-3.txt   TGCGAGGCGGCCTCCAGACCACGTACTCATTACGTTGCCGTCAAGACTGTGGAGCAGCAGGTACTTTCTGTACAACATCGTCTTCGCGAGTCACTGGTTTGCCACCGTACTGAAGCCATCAACAGGGTGC
CI-is7c-5.   TGCGAGGCGGCCTCCAGACCACGTACTCATTACGTTGCCGTCAAGACTGTGGAGCAGCAGGTACTTTCTGTACAACATCGTCTTCGCGAGTCACTGGTTTGCCACCGTACTGAAGCCATCAACAGGGTGC
CI-is7c-6.   TGCGAGGCGGCCTCCAGACCACGTACTCATTACGTTGCCGTCAAGACTGTGGAGCAGCAGGTACTTTCTGTACAACATCGTCTTCGCGAGTCACTGGTTTGCCACCGTACTGAAGCCATCAACAGGGTGC
CI-is7c-8.   TGCGAGGCGGCCTCCAGACCACGTACTCATTACGTTGCCGTCAAGACTGTGGAGCAGCAGGTACTTTCTGTACAACATCGTCTTCGCGAGTCACTGGTTTGCCACCGTACTGAAGCCATCAACAGGGTGC
CI-is7c-9.   TGCGAGGCGGCCTCCAGACCACGTACTCATTACGTTGCCGTCAAGACTGTGGAGCAGCAGGTACTATCTGTACAACATCGTCTTCGCGAGTCACTGGTTTGCCACCGTACTGAAGCCATCAACAGGGTGC
 Consensus   TGCGAGGCGGCCTCCAGACCACGTACTCATTACGTTGCCGTCAAGACTGTGGAGCAGCAGGTACTTTCTGTACAACATCGTCTTCGCGAGTCACTGGTTTGCCACCGTACTGAAGCCATCAACAGGGTGC

             1041                                                                                                                          1170
is7c-2.txt   ATGGTTTTTTGCTTGAGTTCGGAATTGCCTTGCCAGCCACCAAGGCAGCGATCGCCAAAGTGCCTGAGCTGATTGACGATTCACGTAGCGACCTGCCGCTGCAGTTCAAAAGGGTCATGGAGCATTTGCT
is7c-3.txt   ATGGTTTTTTGCTTGAGTTCGGAATTGCCTTGCCAGCCACCAAGGCAGCGATCACCAAAGTGCCTGAGCTGATTGACGATTCACGTAGCGACCTGCCGCTGCAGTTCAAAAGGGTCATGGAGCATTTGCT
CI-is7c-5.   ATGGTTTTTTGCTTGAGTTCGGAATTGCCTTGCCAGCCACCAAGGCAGCGATCGCCAAAGTGCCTGAGCTGATTGACGATTCACGTAGCGACCTGCCGCTGCAGTTCAAAAGGGTCATGGAGCATTTGCT
CI-is7c-6.   ATGGTTTTTTGCTTGAGTTCGGAATTGCCTTGCCAGCCACCAAGGCAGCGATCGCCAAAGTGCCTGAGCTGATTGACGATTCACGTAGCGACCTGCCGCTGCAGTTCAAAAGGGTCATGGAGCATTTGCT
CI-is7c-8.   ATGGTTTTTTGCTTGAGTTCGGAATTGCCTTGCCAGCCACCAAGGCAGCGATCGCCAAAGTGCCTGAGCTGATTGACGATTCACGTAGCGACCTGCCGCTGCAGTTCAAAAGGGTCATGGAGCATTTGCT
CI-is7c-9.   ATGGTTTTTTGCTTGAGTTCGGAATTGCCTTGCCAGCCACCAAGGCAGCGATCGCCAAAGTGCCTGAGCTGATTGACGATTCACGTAGCGACCTGCCGCTGCAGTTCAAAAGGGTCATGGAGCATTTGCT
 Consensus   ATGGTTTTTTGCTTGAGTTCGGAATTGCCTTGCCAGCCACCAAGGCAGCGATCGCCAAAGTGCCTGAGCTGATTGACGATTCACGTAGCGACCTGCCGCTGCAGTTCAAAAGGGTCATGGAGCATTTGCT

             1171                                                                                                                          1300
is7c-2.txt   GGAGGATATTCATCGCCTTGATACCGAAATAAAGGCCTGTGATGCGCAAATCAAGCAGCAACTGGCGCAAGACGATGCTGGTACACGCTTGATGACGATTCCTGGCATTGGTCCTATTACCGCCAGCGCA
is7c-3.txt   GGAGGATATTCATCGCCTTGATACCGAAATAAAGGCATGTGATGCGCAAATCAAGCAGCAACTGGCGCAAGACGATGCTGGTACACGCTTGATGACGATTCCTGGCATTGGTCCTATTACCGCCAGCGCA
CI-is7c-5.   GGAGGATATTCATCGCCTTGATACCGAAATAAAGGCCTGTGATGCGCAAATCAAGCAGCAACTGGCGCAAGACGATGCTGGTACACGCTTGATGACGATTCCTGGCATTGGTCCTATTACCGCCAGCGCA
CI-is7c-6.   GGAGGATATTCATCGCCTTGATACCGAAATAAAGGCCTGTGATGCGCAAATCAAGCAGCAACTGGCGCAAGACGATGCTGGTACACGCTTGATGACGATTCCTGGCATTGGTCCTATTACCGCCAGCGCA
CI-is7c-8.   GGAGGATATTCATCGCCTTGATACCGAAATAAAGGCCTGTGATGCGCAAATCAAGCAGCAACTGGCGCAAGACGATGCTGGTACACGCTTGATGACGATTCCTGGCATTGGTCCTATTACCGCCAGCGCA
CI-is7c-9.   GGAGGATATTCATCGCCTTGATACCGAAATAAAGGCCTGTGATGCGCAAATCAAGCAGCAACTGGCGCAAGACGATGCTGGTACACGCTTGATGACGATTCCTGGCATTGGTCCTATTACCGCCAGCGCA
 Consensus   GGAGGATATTCATCGCCTTGATACCGAAATAAAGGCCTGTGATGCGCAAATCAAGCAGCAACTGGCGCAAGACGATGCTGGTACACGCTTGATGACGATTCCTGGCATTGGTCCTATTACCGCCAGCGCA
```

```
              1301                                                                                                                             1430
is7c-2.txt    TTTGTAGCGGATCTGGGTGACGCTTCCAATTTTCGAGCCTCACGAGACCTTTCCGCTTTTCTGGGGCTGGTTCCCAGACAGTTCTCTACAGGCGGGAGACCCGTATTGCTGGGTATCAGCAAGCGAGGTG
is7c-3.txt    TTTGTAGCGGATCTGGGTGACGCTTCCAATTTTCGAGCCTCACGAGACCTTTCTGCTTTTCTGGGGCTGGTTCCCAGACAGTTCTCTACAGGCGGGAGACCCGTATTGCTGGGTATCAGCAAGCGAGGTG
CI-is7c-5.    TTTGTAGCGGATCTGGGTGACGCTTCCAATTTTCGAGCCTCACGAGACCTTTCCGCTTTTCTGGGGCTGGTTCCCAGACAGTTCTCTACAGGCGGGAGACCCGTATTGCTGGGTATCAGCAAGCGAGGTG
CI-is7c-6.    TTTGTAGCGGATCTGGGTGACGCTTCCAATTTTCGAGCCTCACGAGACCTTTCCGCTTTTCTGGGGCTGGTTCCCAGACAGTTCTCTACAGGCGGGAGACCCGTATTGCTGGGTATCAGCAAGCGAGGTG
CI-is7c-8.    TTTGTAGCGGATCTGGGTGACGCTTCCAATTTTCGAGCCTCACGAGACCTTTCCGCTTTTCTGGGGCTGGTTCCCAGACAGTTCTCTACAGGCGGGAGACCCGTATTGCTGGGTATCAGCAAGCGAGGTG
CI-is7c-9.    TTTGTAGCGGATCTGGGTGACGCTTCCAATTTTCGAGCCTCACGAGACCTTTCCGCTTTTCTGGGGCTGGTTCCCAGACAGTTCTCTACAGGCGGGAGACCCGTATTGCTGGGTATCAGCAAGCGAGGTG
 Consensus    TTTGTAGCGGATCTGGGTGACGCTTCCAATTTTCGAGCCTCACGAGACCTTTCCGCTTTTCTGGGGCTGGTTCCCAGACAGTTCTCTACAGGCGGGAGACCCGTATTGCTGGGTATCAGCAAGCGAGGTG

              1431                                                                                                                             1560
is7c-2.txt    ACAGGCACCTGCGAACCCTGTTGGTGCAAGGTTCTCGAGCGCTCATGATTCGAATAGAACGCCGCGATGATGCCCTTGGGCAGTGGGTAAGAGACTTGTTGGCACGCAAGCATCCGAACAAGGTGGCATG
is7c-3.txt    ACAGGCACCTGCGAACCCTGCTGGTGCAAGGTTCTCGAGCGCTCATGATTCGAATAGAACGCCGCGATGATGCCCTTGGGCAGTGGGTAAGAGACTTGTTGGCACGCAAGCATCCGAACAAGGTGGCATG
CI-is7c-5.    ACAGGCACCTGCGAACCCTGTTGGTGCAAGGTTCTCGAGCGCTCATGATTCGAATAGAACGCCGCGATGATGCCCTTGGGCAGTGGGTAAGAGACTTGTTGGCACGCAAGCATCCGAACAAGGTGGCATG
CI-is7c-6.    ACAGGCACCTGCGAACCTTGTTGGTGCAAGGTTCTCGAGCGCTCATGATTCGAATAGAACGCCGCGATAATGCCCTTGGGCAGTGGGTAAGAGACTTGTTGGCACGCAAGCATCCGAACAAGGTGGCATG
CI-is7c-8.    ACAGGCACCTGCGAACCCTGTTGGTGCAAGGTTCTCGAGCGCTCATGATTCGAATAGAACGCCGCGATGATGCCCTTGGGCAGTGGGTAAGAGACTTGTTGGCACGCAAGCATCCGAACAAGGTGGCATG
CI-is7c-9.    ACAGGCACCTGCGAACCCTGTTGGTGCAAGGTTCTCGAGCGCTCATGATTCGAATAGAACGCCGCGATGATGCCCTTGGGCAGTGGGTAAGAGACTTGTTGGCACGCAAGCATCCGAACAAGGTGGCATG
 Consensus    ACAGGCACCTGCGAACCCTGTTGGTGCAAGGTTCTCGAGCGCTCATGATTCGAATAGAACGCCGCGATGATGCCCTTGGGCAGTGGGTAAGAGACTTGTTGGCACGCAAGCATCCGAACAAGGTGGCATG

              1561                                                                                                                             1690
is7c-2.txt    TGCTTTAGCGAACAAGCTGGCAAGAATCGTATGGGCGGTGCTCAGACGAGGTAGCACTTACAACGCATCAATGATCTGAGTAGGACATAGGCGTACCGGCTTTTGCGACGCAGTGATGATGACGATAAAC
is7c-3.txt    TGCTTTAGCGAACAAGCTGGCAAGAATCGTATGGGCGGTGCTCAGACGAGGTAGCACTTACAACGCATCAATGATCTGAGTAGGACATAGGCGTACCGGCTTTTGCGACGCAGTGATGATGACGATAAAC
CI-is7c-5.    TGCTTTAGCGAACAAGCTGGCAAGAATCGTATGGGCGGTGCTCAGACGAGGTAGCACTTACAACGCATCAATGATCTGAGTAGGACATAGGCGTACCGGCTTTTGCGACGCAGTGATGATGACGATAAAC
CI-is7c-6.    TGCTTTAGCGAACAAGCTGGCAAGAATCGTATGGGCGGTGCTCAGACGAGGTAGCACTTACAACGCATCAATGATCTGAGTAGGACATAGGCGTACCGGCTTTTGCGACGCAGTGATGATGACGATAAAC
CI-is7c-8.    TGCTTTAGCGAACAAGCTGGCAAGAATCGTATGGGCGGTGCTCAGACGAGGTAGCACTTACAACGCATCAATGATCTGAGTAGGACATAGGCGTACCGGCTTTTGCGACGCAGTGATGATGACGATAAAC
CI-is7c-9.    TGCTTTAGCGAACAAGCTGGCAAGAATCGTATGGGCGGTGCTCAGACGAGGTAGCACTTACAACGCATCAATGATCTGAGTAGGACATAGGCGTACCGGCTTTTGCGACGCAGTGATGATGACGATAAAC
 Consensus    TGCTTTAGCGAACAAGCTGGCAAGAATCGTATGGGCGGTGCTCAGACGAGGTAGCACTTACAACGCATCAATGATCTGAGTAGGACATAGGCGTACCGGCTTTTGCGACGCAGTGATGATGACGATAAAC

              1691                                                                                                                             1820
is7c-2.txt    AGCTCATGGCCAGACTGAAAACCTGATAG-CAATGCAGCCTTCGAGGCTTTAGGGATTTTTGAGGACAGTAAGGCGGCGAATACTCATCAAGGGCCGGGCAGCGAAATTGCCCAGAATGAGCCCGGATAC
is7c-3.txt    AGCTCATGGCCAGACTGAAAACCTGATAGGCAAAGCAGCCTTCGAGGCTTTAGGGATTTTTGAGGACAGTAAGGCGGCGAATACTCATCAAGGGCCGGGCAGCGAAATTGCCCAGAATGAGCCCGGATAC
CI-is7c-5.    AGCTCATGGCCAGACTGAAAACCTGATAGGCAAAGCAGCCTTCGAGGCTTTAGGGATTTTTGAGGACAGTAAGGCGGCGAATACTCATCAAGGGCCGGGCAGCGAAATTGCCCAGAATGAGCCCGGATAC
CI-is7c-6.    AGCTCATGGCCAGACTGAAAACCTGATAGGCAAAGCAGCCTTCGAGGCTTTAGGGATTTTTGAGGACAGTAAGGCGGCGAATACTCATCAAGGGCCGGGCAGCGAAATTGCCCAGAATGAGCCCGGATAC
CI-is7c-8.    AGCTCATGGCCAGACTGAAAACCTGATAGGCAAAGCAGCCTTCGAGGCTTTAGGGATTTTTGAGGACAGTAAGGCGGCGAATACTCATCAAGGGCCGGGCAGCGAAATTGCCCAGAATGAGCCCGGATAC
CI-is7c-9.    AGCTCATGGCCAGACTGAAAACCTGATAAGCAATGCAGCCTTCGAGGCTTTAGGGATTTTTGAGGACAGTAAGGCGGCGAATACTCATCAAGGGCCGGGCAGCGAAATTGCCCAGAATGAGCCCGGATAC
 Consensus    AGCTCATGGCCAGACTGAAAACCTGATAGGCAAaGCAGCCTTCGAGGCTTTAGGGATTTTTGAGGACAGTAAGGCGGCGAATACTCATCAAGGGCCGGGCAGCGAAATTGCCCAGAATGAGCCCGGATAC

              1821                                                                                                                             1950
is7c-2.txt    ATTAGCGCAAGCATGCTTTACCGCTCATCGAACTGCGATTGCAGGAGGTGGGCTGACCATACATTTGCTTACTTTGGCCCCTTCAAAGTAAGTCGCCGAGGGGCGAAAAGGTGACCTGAGCCGAAAACAG
is7c-3.txt    ATTAGCGCAAGCATGCTTTACCGCTCATCGAACTGCGATTGCAGGAGGTGGGCTGACCATACATTTGCCTACTTTGGTCCCT-CAAAGTAGGGCGCCGAGGGGCGAAAAGGTGCCTTGAGCCTTAAACAC
CI-is7c-5.    ATTAGCGCAAGCATGCTTTACCGCTCATCGAACTGCGATTGCAGGAGGTGGGCTGACCATACATTTGCTTACTTTGATCC-TTCAAAGTCAATCGCCGAGAGGCGAAAAGGTAACTTGAGTCGTAAACAA
CI-is7c-6.    ATTAGCGCAAGCATGCTTTACCGCTCATCGAACTGCGATTGCAGGAGGTGGGCTGACCATACATTTGCCTACTTTGGCCCCATCAAAGTAGGTCGCCGAGGGGCGAAAAGGTGTCTTGAGTCGAGCCCGA
CI-is7c-8.    ATTAGCGCAAGCATGCTTTACCGCTCATCGAACTGCGATTGCAGGAGGTGGGCTGACCATACATTTGCCTACTTTGGCCCCATCAAAGTAGGTCGCCGAGGGGCGAAAAGGTGCCTCGAGCCGTAAACAA
CI-is7c-9.    ATTAGCGCAAGCATGCTTTACCGCTCATCGAACTGCGATTGCAGGAGGTGGGCTGACCATACATTTGCTTACTTTGGTCCCT-CAAAGTAGGGCGCCGAGAGGCGAAAAGGTACCTTGAGTCGTAAACAA
 Consensus    ATTAGCGCAAGCATGCTTTACCGCTCATCGAACTGCGATTGCAGGAGGTGGGCTGACCATACATTTGC.TACTTTGG.CCCttCAAAGTAgGtCGCCGAGgGGCGAAAAGGTg.CTTGAG.CGtAAACAa
```

```
            1951                                                                                                                              2080
is7c-2.txt  ACATCACTGACATTGCAGAACCGCTTTGATGCAGAGCGTTACGAACACAAAGAGGACGCAGAGCGTCCAGAACTGCATGCCGACG------CGGAGCATCGGCACGATAGTCATGCACAAGCCTGCTTCT
is7c-3.txt  ATCCAACTGACATAGCTGAACCGCAGTGTAACGTTCCACGTCACATATCTGCGTCGCACGATAGTTGAGATTATCGCTCCCTGCGTTTCAGCGTTGGAATGCCTCGGGTGACGCTCTGCTTCAAGTCTGT
CI-is7c-5.  ACATCACTGATATCACAGAACCGCAGTGTGATGCTCCGCGCCACAGACCTGCGCGGCATACTCAGGATAGGACGCAGAGCGTCCA-------GAACGGCGTGCGACGCAGAGCGTCGCACGATAATTGAA
CI-is7c-6.  ATCTCACTGAC-TCGCAGAACCAACATGTGGCGCGAAGCGCCACAAA----GAGGACGCAGAGCGTCCAGAACAGCATGCGACGCGGAGCGTCGCACGATAGTTGAAAAAACGCTTTTTTATCGCAATGG
CI-is7c-8.  ACATCACTGACCTCGCCGAACCGCCGTGCGACGCAGAGCGTCCAGAACGGCATGCCGACGCGGAGCATCGGCACGATAGTCAGTTAGATTTGTGTCTAACGCTGAGCGCGGGGAGATAATCATCAAAGC
CI-is7c-9.  ACATCACTGACCTTGCAGAACCGCAGTGTGACGCGGAGCGCCACAAA----AAGGACGCAGAGCGTCCAGAACGGCATGCGACGCGGAGCGTCGCACGATAGTTGAAAGAAAGTGCTTTTTTATCGCGGT
 Consensus  AcaTCACTGAC.T.GCaGAACCGC.gTGtgaCGc...GCG.CacA.A......gg.C.Ca..g.Gt..aG.ac.g..tGC.............g....a..g.......ga.g..c............g.

            2081                                                                                                                              2210
is7c-2.txt  GCCCGA--AGGGCTGGGGGCGACCGGGGCGGCGATCCGCATTGTTCGCGAAGACTGTGTTTCAGGCGACCCGATTTCTGCGACTGTACGGGCCATTTCGCGAACAAGTTCGCTCCCACGGATCCTGACTT
is7c-3.txt  GCCACACCACAACGTCATGCCTTGCCTCTAGTCACTGCCTATAAACGAAAGCGTTTTTTTACCGCCATGGCACTGACGACGCAGAATGAGGCGTAAGTGACGGCTGAGCCAGAGCACAGGAGCGAAAACT
CI-is7c-5.  GCGGCATCACGACGGAGCGCCGCACGATGGTTGAAAATGCAGCGCCTGGGCCATCGTCTTCTTGAGCAAGCTCACCCAACAGGTGCATATCCAGCCTGATACTGCAATTCACCCACACCCTTCCAGGGCT
CI-is7c-6.  CACTGACGACGCAGAGTGCGACGTAAGTGACGGCTGAGCCCTGGCGCTGATCCGGGGGATGCGAGGCAGGACGCCGAGCAAGCTGCACCGGGCCAAGGATGGCCCGTTGCAGCGACCCCCGGATCAGTGT
CI-is7c-8.  CCCACTTTTCGTGCCGTCAGTTCAGCAACGCAACCGTCTCCGGCACAAAGTCG---------AACGAGCCGAGATTCATCCCCTGTTCATCGACCTGCAGCACCCAGCCCTGACGGTCCCAGTCACCCAG
CI-is7c-9.  GGCACCGACGACGCAGAGTGCGACGTAAAGAACCGAGAGGTGTCCGCCAGAGCACCTCTCGATCCATCAGCAAAAACGCAACAAAACGTGGCACAGTCGCCCCGAAAAATGGCACATTGGCGCCCCCAGG
 Consensus  gcC.ca..acg....g............g..............c.....c....t.tt.........gc.....C..c...tg....ggc..........c.a...C.g.....c...gcc.....t

            2211                                2248
is7c-2.txt  CGCTACACTCCCGCCAATCACATCCACTCAAGCAGGAG
is7c-3.txt  TGTTACTGTCGTGCCCATTCTCAACACATT
CI-is7c-5.  CTTCAAGATGTGTGTATAGCCGAGCGCGAAGCGACTTT
CI-is7c-6.  CAGGGCGAAGGAACCCGACGAAGTCGGGCCGGAA
CI-is7c-8.  CACGATACGTTTTGCCGACTGATCGCCAATCTGCAATT
CI-is7c-9.  CGCATGTGACACATTTTGTAATCATCCGTCCGGG
 Consensus  C...a.........c.........c.C...........
```

REP sequences are in green background

Alignment of REP sequences adjacent to ISPsy7

```
            1                                                    54
        2    TGATGCAGAGCGTTACGAACACAAAGAGGACGCAGAGCGTC--CAGAACTGCA
        3    GGACGCAGAGCGTCCAGAACGGCATGC-GACGCGGAGCGTCG-CACGATAGTT
        6   AGGACGCAGAGCGTCCAGAACAGCATGC-GACGCGGAGCGTCG-CACGATAGT
        9   AGGACGCAGAGCGTCCAGAACGGCATGC-GACGCGGAGCGTCG-CACGATAGT
        5   AGGACGCAGAGCGTCCAGAACGGCGTGC-GACGCAGAGCGTCG-CACGATAAT
        8   GCGACGCAGAGCGTCCAGAACGGCATGCCGACGCGGAGCATCGGCACGATA
 Consensus  .gGACGCAGAGCGTCCAGAACgGCATGC.GACGCgGAGCGTCG CACGATAgt.
```

Pseudomonas syringae canonical REP sequence:

GGACGCGGAGCGTCCAGAACGGCATGCCGACGCAGAGCGTCGCACGATAGT
