# Supplementary material for: Bacterial repetitive extragenic palindromic sequences are DNA targets for Insertion Sequence elements
Source: BMC Genomics. 2006 Mar 24;7:62. doi: 10.1186/1471-2164-7-62 (PMC1525189; doi:10.1186/1471-2164-7-62)
Supplement: Additional File 10 — Alignment of DNA sequences from all copies of ISRm5 in Sinorhizobium meliloti and their flanking regions. [file 1471-2164-7-62-S10.pdf]

```
           391                                                                                                                              520
1'-188355-
8'-3222325
2'-190008- CCCGCCTGCGGGGAGAAGGTGGCGGCAGCCGGTTGAGGGGCAATCCCTTCCCTCGAACCCACCAGGTCTGCAAACGGCAAATCTCGAGGATGACGACCGAGGGAGGTTGAGGCGGTTGGGTTCCCGAGGG
3'-1677017 GTGCGAGCAACAACACGATCTTGCGCATAGGCCCCCGTACCTTTTCTAACAGTTTGACGGCCATTGATATTCTGCCATTCGCGCAGGATCAATTCCCAACCGCGCAATCCACACGGTCCTGCGGCAGCGC
4-1853481- AAGATTGTCCCTTCTCCGCAGCTCTGAGGCTCCACCAACTACAGCGCATTGGGGAATTGATTGATGCAAGACAGAGACGACCACGCGTTGCCGGAGCAAAGTGCATCATCCGAAGAGAATACGGGACTAG
10-70201-7                          TCGAGAACATAGAGTTGCCATCCCGCCGCCCGATCGCGGCGGCCCAGCGCAGGAATGCCGATCTCAGAAACAGGCTGAGGACGTTCTTCTACCGCAGGTTTTTCC
5'-2402373 CCCGCCTGCGGGGAGAAGGTGGCGGCAGCCGGTTGAGGGGCAATCCCTTCCCTCGAACCCACCAGGTCTGCAAACGGCAAATCTCGAGGATGACGACCGAGGGAGGTTGAGGCGGTTGGGTTCCCGAGGG
7'-2978252 CGGCACCTTCTCCCCGCAGGCGGGGAGAAGGGACAAGCGGCGCCGACTGAACCCCGCATTCACCGCCCGCCCGAGGGCGCGAGCGGGGCGCGGCATATCCCTTCTCCCCGCCTGCGGGGAGAAGGTGGCG
6-2602149- CGGGATCCGGGTGGGTATCAGCAATATCCCCTCTGCCCTGCCGCGCATCTCCCCCGCAGGGCAGATTGTCCCGAAGTAAGGCCCTGCCCCTCGGTCTGCCCCTCACCCTAACCCTCTCCCCGCAAGCGGG
9'-3224074 CCGGCACCTTCTCCCCGCAGGCGGGGAGAAGGGACAAGCGGCGCCGACTGAACCCCGCATTCACCGCCCGCCCGAGGGAGCGAGCGGGCGCGGCATATCCCTTCTCCCCGCCTGCGGGGAGAAGGTGGCG
 Consensus .....................................................c.......c...g.........g.........g...........................................g

           521                                                                                                                              650
1'-188355-
8'-3222325
2'-190008- ATCCCGGGATCCATTCATCAGGGTAGTGTGCTGAAGGTTCTGCAAATTCACTGAGAGAGGCGGTCATGGCAGGCTGGTGATGTTGACGTTACCTGATTCCCTGGAAGGAACGCCACCATGACCAAGACTG
3'-1677017 TTCTTACACCATCCGGGCTCCGGTAGTGTGCTGAAGGTTCTGCAAATTCACTGAGAGAGGCGGTCATGGCAGGCTGGTGATGTTGACGTCACCTGATTCCCTGGAAGGAACGCCACCATGACCAAGACTG
4-1853481- AAACTGGAAAGAAAACGATTGGGTAGTGTGCTGAAGGTTCTGCAAATTCACTGAGAGAGGCGGTCATGGCAGGCTGGTGATGTTGACGTCACCTGATTCCCTGGAAGGAACGCCACCATGACCAAGACTG
10-70201-7 TCGTCTGCTCGCGGCCTTCGGGGTAGTGTGCTGAAGGTTCTGCAAATTCACTGAGAGAGGCGGTCATGGCAGGCTGGTGATGTTGACGTCACCTGATTCCCTGGAAGGAACGCCACCATGACCAAGACTG
5'-2402373 ATCCCGGGATCCATTCATCAGGGTAGTGTGCTGAAGGTTCTGCAAATTCACTGAGAGAGGCGGTCATGGCAGGCTGGTGATGTTGACGTTACCTGATTCCCTGGAAGGAACGCCACCATGACCAAGACTG
7'-2978252 GCAGCCGGTTGAGGGGCAAGCGGTAGTGTGCTGAAGGTTCTGCAAATTCACTGAGAGAGGCGGTCATGGCAGGCTGGTGATGTTGACGTTACCTGATTCCCTGGAAGGAACGCCACCATGACCAAGACTG
6-2602149- GCGAGGGGACGAGTTCGCTTTGGTAGTGTGCTGAAGGTTCTGCAAATTCACTGAGAGAGGCGGTCATGGCAGGCTGGTGATGTTGACGTCACCTGATTCCCTGGAAGGAACGCCACCATGACCAAGACTG
9'-3224074 GCAGCCGGTTGAGGGGCAAGCGGTAGTGTGCTGAAGGTTCTGCAAATTCACTGAGAGAGGCGGTCATGGCAGGCTGGTGATGTTGACGTTACCTGATTCCCTGGAAGGAACGCCACCATGACCAAGACTG
 Consensus ......g..............GGTAGTGTGCTGAAGGTTCTGCAAATTCACTGAGAGAGGCGGTCATGGCAGGCTGGTGATGTTGACGT.ACCTGATTCCCTGGAAGGAACGCCACCATGACCAAGACTG

           651                                                                                                                              780
1'-188355-
8'-3222325
2'-190008- AAGGTAAGACAGCCAGCGCCGCCGTCAAAGACATTTTGCTTTCGAACCCCGATGGGCTGCGCGAGGTGATCCGCACGGTGATGCAGGAGGTGCTGGAAGCGGAGATGGACGAGGCGCTGGGAGCTGCGAA
3'-1677017 AAGGTAGGACAGCCAGCGCCGCCGTCAAAGACATTTTGCTTTCGAACCCCGATGGCCTGCGCGAGGTGATCCGCACGGTGATGCAGGAGGTGCTGGAAGCGGAGATGGACGAGGCGCTGGGAGCTGCGAA
4-1853481- AAGGTAAGACAGCCAGCGCCGCCGTCAAAGACATTTTGCTTTCGAACCCCGATGGCCTGCGCGAGGTGATCCGCACGGTGATGCAGGAGGTGCTGGAAGCGGAGATGGACGAGGCGCTGGGAGCTGCGAA
10-70201-7 AAGGTAGGACAGCCAGCGCCGCCGTCAAAGACATTTTGCTTTCGAACCCCGATGGGCTGCGCGAGGTGATCCGCACGGTGATGCAGGAGGTGCTGGAAGCGGAGATGGACGAGGCGCTGGGAGCTGCGAA
5'-2402373 AAGGTAAGACAGCCAGCGCCGCCGTCAAAGACATTTTGCTTTCGAACCCCGATGGGCTGCGCGAGGTGATCCGCACGGTGATGCAGGAGGTGCTGGAAGCGGAGATGGACGAGGCGCTGGGAGCTGCGAA
7'-2978252 AAGGTAAGACAGCCAGCGCCGCCGTCAAAGACATTTTGCTTTCGAACCCCGATGGGCTGCGCGAGGTGATCCGCACGGTGATGCAGGAGGTGCTGGAAGCGGAGATGGACGAGGCGCTGGGAGCTGCGAA
6-2602149- AAGGTAAGACAGCCAGCGCCGCCGTCAAAGACATTTTGCTTTCGAACCCCGATGGCCTGCGCGAGGTGATCCGCACGGTGATGCAGGAGGTGCTGGAAGCGGAGATGGACGAGGCGCTGGGAGCTGCGAA
9'-3224074 AAGGTAAGACAGCCAGCGCCGCCGTCAAAGACATTTTGCTTTCGAACCCCGATGGGCTGCGCGAGGTGATCCGCACGGTGATGCAGGAGGTGCTGGAAGCGGAGATGGACGAGGCGCTGGGAGCTGCGAA
 Consensus AAGGTAaGACAGCCAGCGCCGCCGTCAAAGACATTTTGCTTTCGAACCCCGATGG.CTGCGCGAGGTGATCCGCACGGTGATGCAGGAGGTGCTGGAAGCGGAGATGGACGAGGCGCTGGGAGCTGCGAA
```

```
            781                                                                                                                         910
1'-188355-
8'-3222325
2'-190008-  AGGCGAGCGCACGCCGGAGCGGCTCGGCTACCGTTCGGGCCACTATGGCCGCACGCTGATCACGCGGGTGGGCAAGCTCGAGCTGCGGGTGCCGCAGGATCGCTCGGGGCACTTCTCCACCGAATTGTTC
3'-1677017  AGGCGAGCGCACGCCGGAGCGGCTCGGCTACCGTTCGGGCCACTATGGCCGCACGCTGATCACGCGGGTGGGCAAGCTCGAGCTGCGGGTGCCGCAGGATCGCTCGGGGCACTTCTCCACCGAATTGTTC
4-1853481-  AGGCGAGCGCACGCCGGAGCGGCTCGGCTACCGTTCGGGCCACTATGGCCGCACGCTGATCACGCGGGTGGGCAAGCTCGAGCTGCGGGTGCCGCAGGATCGCTCGGGGCACTTCTCCACCGAATTGTTC
10-70201-7  AGGCGAGCGCACGCCGGAGCGGCTCGGCTACCGTTCGGGCCACTATGGCCGCACGCTGATCACGCGGGTGGGCAAGCTCGAGCTGCGGGTGCCGCAGGATCGCTCCGGGCACTTCTCCACCGAATTGTTC
5'-2402373  AGGCGAGCGCACGCCGGAGCGGCTCGGCTACCGTTCGGGCCACTATGGCCGCACGCTGATCACGCGGGTGGGCAAGCTCGAGCTGCGGGTGCCGCAGGATCGCTCGGGGCACTTCTCCACCGAATTGTTC
7'-2978252  AGGCGAGCGCACGCCGGAGCGGCTCGGCTACCGTTCGGGCCACTATGGCCGCACGCTGATCACGCGGGTGGGCAAGCTCGAGCTGCGGGTGCCGCAGGATCGCTCGGGGCACTTCTCCACCGAATTGTTC
6-2602149-  AGGCGAGCGCACGCCGGAGCGGCTCGGCTACCGTTCGGGCCACTATGGCCGCACGCTGATCACGCGGGTGGGCAAGCTCGAGCTGCGGGTGCCGCAGGATCGCTCGGGGCACTTCTCCACCGAATTGTTC
9'-3224074  AGGCGAGCGCACGCCGGAGCGGCTCGGCTACCGTTCGGGCCACTATGGCCGCACGCTGATCACGCGGGTGGGCAAGCTCGAGCTGCGGGTGCCGCAGGATCGCTCGGGGCACTTCTCCACCGAATTGTTC
 Consensus  AGGCGAGCGCACGCCGGAGCGGCTCGGCTACCGTTCGGGCCACTATGGCCGCACGCTGATCACGCGGGTGGGCAAGCTCGAGCTGCGGGTGCCGCAGGATCGCTCgGGGCACTTCTCCACCGAATTGTTC

            911                                                                                                                        1040
1'-188355-
8'-3222325
2'-190008-  GAACGCTATCAGCGCTCCGAGCGGGCGCTGGTGGCAACCTTGGCGGAGATGTATGTGCAAGGGGTGTCGACGCGGAAGGTCAAGGCGATCACCGAGGAGCTGTGCGGCCATGCCTTCTCGGCGTCATCGA
3'-1677017  GAACGCTATCAGCGCTCCGAGCGGGCGCTGGTGGCAACCTTGGCGGAGATGTATGTGCAAGGGGTGTCGACGCGGAAGGTCAAGGCGATCACCGAGGAGCTGTGCGGCCATGCCTTCTCGGCGTCATCGA
4-1853481-  GAACGCTATCAGCGCTCCGAGCGGGCGCTGGTGGCAACCTTGGCGGAGATGTATGTGCAAGGGGTGTCGACGCGGAAGGTCAAGGCGATCACCGAGGAGCTGTGCGGCCATGCCTTCTCGGCGTCATCGA
10-70201-7  GAACGCTATCAGCGCTCCGAGCGGGCGCTGGTGGCAACCTTGGCGGAGATGTATGTGCAAGGGGTGTCGACGCGGAAGGTCAAGGCGATCACCGAGGAGCTGTGCGGCCATGCCTTCTCGGCGTCATCGA
5'-2402373  GAACGCTATCAGCGCTCCGAGCGGGCGCTGGTGGCAACCTTGGCGGAGATGTATGTGCAAGGGGTGTCGACGCGGAAGGTCAAGGCGATCACCGAGGAGCTGTGCGGCCATGCCTTCTCGGCGTCATCGA
7'-2978252  GAACGCTATCAGCGCTCCGAGCGGGCGCTGGTGGCAACCTTGGCGGAGATGTATGTGCAAGGGGTGTCGACGCGGAAGGTCAAGGCGATCACCGAGGAGCTGTGCGGCCATGCCTTCTCGGCGTCATCGA
6-2602149-  GAACGCTATCAGCGCTCCGAGCGGGCGCTGGTGGCAACCTTGGCGGAGATGTATGTGCAAGGGGTGTCGACGCGGAAGGTCAAGGCGATCACCGAGGAGCTGTGCGGCCATGCCTTCTCGGCGTCATCGA
9'-3224074  GAACGCTATCAGCGCTCCGAGCGGGCGCTGGTGGCAACCTTGGCGGAGATGTATGTGCAAGGGGTGTCGACGCGGAAGGTCAAGGCGATCACCGAGGAGCTGTGCGGCCATGCCTTCTCGGCGTCATCGA
 Consensus  GAACGCTATCAGCGCTCCGAGCGGGCGCTGGTGGCAACCTTGGCGGAGATGTATGTGCAAGGGGTGTCGACGCGGAAGGTCAAGGCGATCACCGAGGAGCTGTGCGGCCATGCCTTCTCGGCGTCATCGA

            1041                                                                                                                       1170
1'-188355-
8'-3222325
2'-190008-  TCTCGGCGATCAACAAGCGGCTGGACGAGAGCCTGAAGGCCTTTGCCGAGCGCTCGCTTGAAGAGCCGTTTGCCTACCTGATCCTCGATGCCCGTTATGAGAAGGTGCGCGAGGCCGGTGTGGTGATGAG
3'-1677017  TCTCGGCGATCAACAAGCGGCTGGACGAGAGCCTGAAGGCCTTTGCCGAGCGCTCGCTTGAAGAGCCGTTTGCCTACCTGATCCTCGATGCCCGTTATGAGAAGGTGCGCGAGGCCGGTGTGGTGATGAG
4-1853481-  TCTCGGCGATCAACAAGCGGCTGGACGAGAGCCTGAAGGCCTTTGCCGAGCGCTCGCTTGAAGAGCCGTTTGCCTACCTGATCCTCGATGCCCGTTATGAGAAGGTGCGCGAGGCCGGTGTGGTGATGAG
10-70201-7  TCTCGGCGATCAACAAGCGGCTGGACGAGAGCCTGAAGGCCTTTGCCGAGCGCTCGCTTCAAGAGCCGTTTGCCTACCTGATCCTCGATGCCCGTTATGAGAAGGTGCGCGAGGCCGGTGTGGTGATGAG
5'-2402373  TCTCGGCGATCAACAAGCGGCTGGACGAGAGCCTGAAGGCCTTTGCCGAGCGCTCGCTTGAAGAGCCGTTTGCCTACCTGATCCTCGATGCCCGTTATGAGAAGGTGCGCGAGGCCGGTGTGGTGATGAG
7'-2978252  TCTCGGCGATCAACAAGCGGCTGGACGAGAGCCTGAAGGCCTTTGCCGAGCGCTCGCTTGAAGAGCCGTTTGCCTACCTGATCCTCGATGCCCGTTATGAGAAGGTGCGCGAGGCCGGTGTGGTGATGAG
6-2602149-  TCTCGGCGATCAACAAGCGGCTGGACGAGAGCCTGAAGGCCTTTGCCGAGCGCTCGCTTGAAGAGCCGTTTGCCTACCTGATCCTCGATGCCCGTTATGAGAAGGTGCGCGAGGCCGGTGTGGTGATGAG
9'-3224074  TCTCGGCGATCAACAAGCGGCTGGACGAGAGCCTGAAGGCCTTTGCCGAGCGCTCGCTTGAAGAGCCGTTTGCCTACCTGATCCTCGATGCCCGTTATGAGAAGGTGCGCGAGGCCGGTGTGGTGATGAG
 Consensus  TCTCGGCGATCAACAAGCGGCTGGACGAGAGCCTGAAGGCCTTTGCCGAGCGCTCGCTTgAAGAGCCGTTTGCCTACCTGATCCTCGATGCCCGTTATGAGAAGGTGCGCGAGGCCGGTGTGGTGATGAG
```

```
            1171                                                                                                                          1300
1'-188355-
8'-3222325
2'-190008-  CCAGGCGGTGTTGATCGCTGTCGGCATCGACTGGGACGGACGGCGGCAGATCCTGTCGGTCGAGATGGCCGGCCGTGAAAGCCGTTCCGCCTGGAAGGACTTCCTCGTCAGGCTGAAAGGGCGCGGTTTG
3'-1677017  CCAGGCGGTGTTGATCGCTGTCGGCATCGACTGGGACGGACGGCGGCAGATCCTGTCGGTCGAGATGGCCGGCCGTGAAAGCCGTTCCGCCTGGAAGGACTTCCTCGTCAGGCTGAAAGGGCGCGGTTTG
4-1853481-  CCAGGCGGTGTTGATCGCTGTCGGCATCGACTGGGACGGACGGCGGCAGATCCTGTCGGTCGAGATGGCCGGCCGTGAAAGCCGTTCCGCCTGGAAGGACTTCCTCGTCAGGCTGAAAGGGCGCGGTTTG
10-70201-7  CCAGGCGGTGTTGATCGCTGTCGGCATCGACTGGGACGGACGGCGGCAGATCCTGTCGGTCGAGATGGCCGGCCGTGAAAGCCGTTCCGCCTGGAAGGACTTCCTCGTCAGGCTGAAAGGGCGCGGTTTG
5'-2402373  CCAGGCGGTGTTGATCGCTGTCGGCATCGACTGGGACGGACGGCGGCAGATCCTGTCGGTCGAGATGGCCGGCCGTGAAAGCCGTTCCGCCTGGAAGGACTTCCTCGTCAGGCTGAAAGGGCGCGGTTTG
7'-2978252  CCAGGCGGTGTTGATCGCTGTCGGCATCGACTGGGACGGACGGCGGCAGATCCTGTCGGTCGAGATGGCCGGCCGTGAAAGCCGTTCCGCCTGGAAGGACTTCCTCGTCAGGCTGAAAGGGCGCGGTTTG
6-2602149-  CCAGGCGGTGTTGATCGCTGTCGGCATCGACTGGGACGGACGGCGGCAGATCCTGTCGGTCGAGATGGCCGGCCGTGAAAGCCGTTCCGCCTGGAAGGACTTCCTCGTCAGGCTGAAAGGGCGCGGTTTG
9'-3224074  CCAGGCGGTGTTGATCGCTGTCGGCATCGACTGGGACGGACGGCGGCAGATCCTGTCGGTCGAGATGGCCGGCCGTGAAAGCCGTTCCGCCTGGAAGGACTTCCTCGTCAGGCTGAAAGGGCGCGGTTTG
 Consensus  CCAGGCGGTGTTGATCGCTGTCGGCATCGACTGGGACGGACGGCGGCAGATCCTGTCGGTCGAGATGGCCGGCCGTGAAAGCCGTTCCGCCTGGAAGGACTTCCTCGTCAGGCTGAAAGGGCGCGGTTTG

            1301                                                                                                                          1430
1'-188355-
8'-3222325
2'-190008-  AAGGGCGTCGAACTGGTGGTCTCCGACGACCATGCCGGTCTCGTCGCGGCAATTGGCGAGGTGATCCCGGAAGCTGCCTGGCAGCGCTGTTACGTGCATTTCCTCAGGAACGCCCTCGATCACCTGCCGC
3'-1677017  AAGGGCGTCGAACTGGTGGTCTCCGACGACCATGCCGGTCTCGTCGCGGCAATTGGCGAGGTGATCCCGGAAGCTGCCTGGCAGCGCTGTTACGTGCATTTCCTCAGGAACGCCCTCGATCACCTGCCGC
4-1853481-  AAGGGCGTCGAACTGGTGGTCTCCGACGACCATGCCGGTCTCGTCGCGGCAATTGGCGAGGTGATCCCGGAAGCTGTCTGGCAGCGCTGTTACGTGCATTTCCTCAGGAACGCCCTCGATCACCTGCCGC
10-70201-7  AAGGGCGTCGAACTGGTGGTCTCCGACGACCATGCCGGTCTCGTCGCGGCAATTGGCGAGGTGATCCCGGAAGCTGTCTGGCAGCGCTGTTACGTGCATTTCCTCAGGAACGCCCTCGATCACCTGCCGC
5'-2402373  AAGGGCGTCGAACTGGTGGTCTCCGACGACCATGCCGGTCTCGTCGCGGCAATTGGCGAGGTGATCCCGGAAGCTGCCTGGCAGCGCTGTTACGTGCATTTCCTCAGGAACGCCCTCGATCACCTGCCGC
7'-2978252  AAGGGCGTCGAACTGGTGGTCTCCGACGACCATGCCGGTCTCGTCGCGGCAATTGGCGAGGTGATCCCGGAAGCTGCCTGGCAGCGCTGTTACGTGCATTTCCTCAGGAACGCCCTCGATCACCTGCCGC
6-2602149-  AAGGGCGTCGAACTGGTGGTCTCCGACGACCATGCCGGTCTCGTCGCGGCAATTGGCGAGGTGATCCCGGAAGCTGCCTGGCAGCGCTGTTACGTGCATTTCCTCAGGAACGCCCTCGATCACCTGCCGC
9'-3224074  AAGGGCGTCGAACTGGTGGTCTCCGACGACCATGCCGGTCTCGTCGCGGCAATTGGCGAGGTGATCCCGGAAGCTGCCTGGCAGCGCTGTTACGTGCATTTCCTCAGGAACGCCCTCGATCACCTGCCGC
 Consensus  AAGGGCGTCGAACTGGTGGTCTCCGACGACCATGCCGGTCTCGTCGCGGCAATTGGCGAGGTGATCCCGGAAGCTGcCTGGCAGCGCTGTTACGTGCATTTCCTCAGGAACGCCCTCGATCACCTGCCGC

            1431                                                                                                                          1560
1'-188355-                                               TGTGATGATTCTCTCCTTTTCATCATCATGGCCCACCGCACAGAATTCCTGACAGTCCCGTCCGCTATCCGCGCCTGACCTCCTG
8'-3222325                                                                                          ATGAAACCGGCCCACCGCACAGAATTCCTGACAGTCCCCCTG
2'-190008-  GCAAACATGGCGACGATTGCCTGCAGGAGCTTCGATGGCTCTATGATCGCCGCGACCTTGATGAGGCGAAGGCCGATCTCGCCGCGTGGCTGGGAAAATGGTCGGTCCGCTATGGGACTGTCAGGAATTC
3'-1677017  GCAAACATGGCGACGATTGCCTGCAGGAGCTTCGATGGCTCTATGATCGCCGCGACCTTGATGAGGCGAAGGCCGATCTCGCCGCGTGGCTGGGAAAATGGTCGGTCCGCTATCCGCGCCTGACCTCCTG
4-1853481-  GCAAACATGGCGACGATTGCCTGCAGGAGCTTCGATGGCTCTATGATCGCCGCGACCTTGATGAGGCGAAGGCCGATCTCGCCGCGTGGCTGGGAAAATGGTCGGTCCGCTATCCGCGCCTGACCTCCTG
10-70201-7  GCAAACATGGCGACGATTGCCTGCAGGAGCTTCGATGGCTCTATGATCGCCGCGACCTTGATGAGGCGAAGGCCGATCTCGCCGCGTGGCTGGGAAAATGGTCGGTCCGCTATCCGCGCCTGACCTCCTG
5'-2402373  GCAAACATGGCGACGATTGCCTGCAGGAGCTTCGATGGCTCTATGATCGCCGCGACCTTGATGAGGCGAAGGCCGATCTCGCCGCGTGGCTGGGAAAATGGTCGGTCCGCTATCCGCGCCTGACCTCCTG
7'-2978252  GCAAACATGGCGACGATTGCCTGCAGGAGCTTCGATGGCTCTATGATCGCCGCGACCTTGATGAGGCGAAGGCCGATCTCGCCGCGTGGCTGGGAAAATGGTCGGTCCGCTATCCGCGCCTGACCTCCTG
6-2602149-  GCAAACATGGCGACGATTGCCTGCAGGAGCTTCGATGGCTCTATGATCGCCGCGACCTTGATGAGGCGAAGGCCGATCTCGCCGCGTGGCTGGGAAAATGGTCGGTCCGCTATCCGCGCCTGACCTCCTG
9'-3224074  GCAAACATGGCGACGATTGCCTGCAGGAGCTTCGATGGCTCTATGATCGCCGCGACCTTGATGAGGCGAAGGCCGATCTCGCCGCGTGGCTGGGAAAATGGTCGGTCCGCTATCCGCGCCTGACCTCCTG
 Consensus  GCAAACATGGCGACGATTGCCTGCAGGAGCTTCGATGGCTCTATGATCGCCGCGACCTTGATGAGGCGAAGGCCGATCTCGCCGCGTGGCTGGGAAAATGGTCGGTCCGCTATCCGCGCCTGACCTCCTG
```

```
            1561                                                                                                                              1690
1'-188355-  GGTTGAGGAAACCATCGAGCAGACGCTGACCTTCTTTCGTCTGCCGCGCCAGCATCACAAGCACCTCAAGAGCACCAACATGCTGGAACGCCTCAACGAGGAAATCCGCCGCAGAACCTACGTCGTGCGC
8'-3222325  GGTTGAGGAAACCATCGAGCAGACGCTGACCTTCTTTCGTCTGCCGCGCCAGCATCACAAGCACCTCAAGAGCACCAACATGCTGGAACGCCTCAACGAGGAAATCCGCCGCAGAACCTACGTCGTGCGC
2'-190008-  TGTGCGGTGGGCCGGTTTCATC
3'-1677017  GGTTGAGGAAACCATCGAGCAGACGCTGACCTTCTTTCGTCTGCCGCGCCAGCATCACAAGCACCTCAAGAGCACCAACATGCTGGAACGCCTCAACGAGGAAATCCGCCGCAGAACCTACGTCGTGCGC
4-1853481-  GGTTGAGGAAACCATCGAGCAGACGCTGACCTTCTTTCGTCTGCCGCGCCAGCATCACAAGCACCTCAAGAGCACCAACATGCTGGAACGCCTCAACGAGGAAATCCGCCGCAGAACCTACGTCGTGCGC
10-70201-7  GGTTGAGGAAACCATCGAGCAGACGCTGACCTTCTTTCGTCTGCCGCGCCAGCATCACAAGCACCTCAAGAGCACCAACATGCTGGAACGCCTCAACGAGGAAATCCGCCGCAGAACCTACGTCGTGCGC
5'-2402373  GGTTGAGGAAACCATCGAGCAGACGCTGACCTTCTTTCGTCTGCCGCGCCAGCATCACAAGCACCTCAAGAGCACCAACATGCTGGAACGCCTCAACGAGGAAATCCGCCGCAGAACCTACGTCGTGCGC
7'-2978252  GGTTGAGGAAACCATCGAGCAGACGCTGACCTTCTTTCGTCTGCCGCGCCAGCATCACAAGCACCTCAAGAGCACCAACATGCTGGAACGCCTCAACGAGGAAATCCGCCGCAGAACCTACGTCGTGCGC
6-2602149-  GGTTGAGGAAACCATCGAGCAGACGCTGACCTTCTTTCGTCTGCCGCGCCAGCATCACAAGCACCTCAAGAGCACCAACATGCTGGAACGCCTCAACGAGGAAATCCGCCGCAGAACCTACGTCGTGCGC
9'-3224074  GGTTGGGGACTGTCAGGAATTCTGTGCGGTGGGCCATGATGATGAAAAGGAGAGAATCATCACA
 Consensus  GGTTGAGGAAACCATCGAGCAGACGCTGACCTTCTTTCGTCTGCCGCGCCAGCATCACAAGCACCTCAAGAGCACCAACATGCTGGAACGCCTCAACGAGGAAATCCGCCGCAGAACCTACGTCGTGCGC

            1691                                                                                                                              1820
1'-188355-  ATCTTTCCCAACACCGAGAGCTGCCTACGACTCGTCCGCGCCCTCGCCGTCGAAACCCACGAAAACTGGATGGAGGCCAATCGCTACATCAACATGGACGACCTGCGAGAGCACAAGAAACTCGCACTCC
8'-3222325  ATCTTTCCCAACACCGAGAGCTGCCTACGCCTCGTCCGCGCGCTCGCCGTCGAAACCCACGAAAACTGGATGGAGGCCAATCGCTACATCAACATGGACGACCTGCGAGAGCACAAGAAACTCGCACTCC
2'-190008-
3'-1677017  ATCTTTCCCAACACCGAGAGCTGCCTACGACTCGTCCGCGCCCTCGCCGTCGAAACCCACGAAAACTGGATGGAGGCCAATCGCTACATCAACATGGACGACCTGCGAGAGCACAAGAAACTCGCACTCC
4-1853481-  ATCTTTCCCAACACCGAGAGCTGCCTACGCCTCGTCCGCGCGCTCGCCGTCGAAACCCACGAAAACTGGATGGAGGCCAATCGCTACATCAACATGGACGACCTGCGAGAGCACAAGAAACTCGCACTCC
10-70201-7  ATCTTTCCCAACACCGAGAGCTGCCTACGCCTCGTCCGCGCGCTCGCCGTCGAAACCCACGAAAACTGGATGGAGGCCAATCGCTACATCAACATGGACGACCTGCGAGAGCACAAGAAACTCGCACTCC
5'-2402373  ATCTTTCCCAACACCGAGAGCTGCCTACGACTCGTCCGCGCCCTCGCCGTCGAAACCCACGAAAACTGGATGGAGGCCAATCGCTACATCAACATGGACGACCTGCGAGAGCACAAGAAACTCGCACTCC
7'-2978252  ATCTTTCCCAACACCGAGAGCTGCCTACGCCTCGTCCGCGCGCTCGCCGTCGAAACCCACGAAAACTGGATGGAGGCCAATCGCTACATCAACATGGACGACCTGCGAGAGCACAAGAAACTCGCACTCC
6-2602149-  ATCTTTCCCAACACCGAGAGCTGCCTACGACTCGTCCGCGCCCTCGCCGTCGAAACCCACGAAAACTGGATGGAGGCCAATCGCTACATCAACATGGACGACCTGCGAGAGCACAAGAAACTCGCACTCC
9'-3224074
 Consensus  ATCTTTCCCAACACCGAGAGCTGCCTACG.CTCGTCCGCGC.CTCGCCGTCGAAACCCACGAAAACTGGATGGAGGCCAATCGCTACATCAACATGGACGACCTGCGAGAGCACAAGAAACTCGCACTCC

            1821                                                                                                                              1950
1'-188355-  GTCAAGCCGCATGACCAGCACGCATGACCGCCCAATTTTGCAGAACTTGACGCACACAACCTTCATCAGCAGTATCCGCCGCACCAGCCCGCAAGAGGTGTGTGCCT
8'-3222325  GTCAAGCCGCATGACCAGCACGCATGACCGCCCAATTTTGCAGAACTTGACGCACACAACCGGGCAAGCCCGACCGAGCGGGGGTTGAGGCGGTTGGGTTCCCGAGGGATCCCCTTCATGAGCAGTATCC
2'-190008-
3'-1677017  GTCAAGCCGCATGACCAGCACGCATGACCGCCCAATTTTGCAGAACTTGACGCACACAACCCGGGCTCCTACATCGGTCCCGAGAAATCGGAACCGATTGTCGGAGGCGCGATGCCGCATGCAACGTCTC
4-1853481-  GTCAAGCCGCATGACCAGCACGCATGACCGCCCAATTTTGCAGAACTTGACGCACACAACCAACGATTGCGAAGCGGCGGAAGTCGGTTGGCGTCATCCGCGATGCCGATCCCGGACGCCTGAAGGCGCT
10-70201-7  GTCAAGCCGCATGACCAGCACGCATGACCGCCCAATTTTGCAGAACTTGACGCACACAACCGCCTTCGGGAGATGCCGGGCGCAGGTAGATCACTTCCCAACGCACTAAGGCACGTTGAGATTTGGGATA
5'-2402373  GTCAAGCCGCATGACCAGCACGCATGACCGCCCAATTTTGCAGAACTTGACGCACACAACCCATTTTTGACGGGGAGAAGGACAAGCCGCGAGGCCGCGAGTCCCCTTCGCCCCGCTTGCGGGGAGAAG
7'-2978252  GTCAAGCCGCATGACCAGCACGCATGACCGCCCAATTTTGCAGAACTTGACGCACACAACCGGGGCAATCCCGACCGAGCGGGGGTTGAGGCGGTTGGGTTCCCGAGGGATCCCTTTCATGAGCAGTATC
6-2602149-  GTCAAGCCGCATGACCAGCACGCATGACCGCCCAATTTTGCAGAACTTGACGCACACAACCTTCGCTTTTGTTCGTCGAGGCTGACGCAGACGCGCAACCGTGTCCCCTCTCCCCGCGCGCGGGGAGAGG
9'-3224074
 Consensus  GTCAAGCCGCATGACCAGCACGCATGACCGCCCAATTTTGCAGAACTTGACGCACACAACC............................g.....................c..................
```

REP sequences are in green background
