# Supplementary material for: Bacterial repetitive extragenic palindromic sequences are DNA targets for Insertion Sequence elements
Source: BMC Genomics. 2006 Mar 24;7:62. doi: 10.1186/1471-2164-7-62 (PMC1525189; doi:10.1186/1471-2164-7-62)
Supplement: Additional File 11 — Alignment of DNA sequences from all copies of ISNm1106 in Neisseria meningitidis MC58 with type 2 association with REP sequences and their flanking regions. [file 1471-2164-7-62-S11.pdf]

```
              1                                                                                                                              130
1’-929603-    CGGGAAACTTATGAATCGTCATTCCCGCGCAGGCGGGAATCTGGAATTTCAATGCCTCAAGAATTTATCGGAAAAAACCAAAACCCTTCCGTCATCATTCCCGCAAAAGCGGGAATCTAGAAATGAAAAG
3’-1072218                                                                               AGCCTTTTTA---------------------------------------------
5-1591795-                                                                               AACCAAAAAACCGCAGGTTTCAA----------------------------ATAC
4’-1427636                                  GAAGGCGGG---------------------------------GGATGGGCGTGTAGGCTGCTTTTTTCGT----------------------------AACG
 Consensus    ...........................................................................a.Cc...a..c..c.....tc..............................a...

              131                                                                                                                            260
1’-929603-    CAACAGGAATTTATCGGAAATGACCGAAACTGAACGGACTGGATTCCCTCTTTTGCGGGAATGACGGCGACAGGGTTGCTGTTATAGTGGATGAACAAAAACCGGTACGGCGTTGTCTCGCCTTAGTTCG
3’-1072218    ----AAAATATTCCGCCAAGCAATCCAATG----CCGCCTGAAATC------------------------------------------------------------------------------------
5-1591795-    CTGCGGTTTTTTCTTACACAATAAACAACG----CTTCCACATATC------------------------------------------------------------------------------------
4’-1427636    TTCTGCGTTTTCAGACCGTCGTTTACTGGGTTTACTGACTGAAATG--------------------------------------AAACCAAAGTGCAGGCTGCCGGAAAGACTTTTTCTGTCTTTTTAGG
 Consensus    ........ttTt....ca....a..caa.g....C.g.CtgaaaTc....................................................................................

              261                                                                                                                            390
1’-929603-    AAGAGAACGATTCTCTAAGGTGCTGAAGCACCAAGTGAATCGGTTCCGTACTATCTGTACTTTCTGCGGCTTCGTCGTCTTGTCCTGATTTTTGTTAATTCACTATATCGACATCGCCAAACGAAACTTC
3’-1072218    -----------TCATAATGTTTC-----------------------------------------------------------------------------------------------------------
5-1591795-    -----------CCACACTCCT-------------------------------------------------------------------------------------------------------------
4’-1427636    CAGCCTGCACTTCTTTTTCACTC-----------------------------------------------------------------------------------------------------------
 Consensus    ...........tC....t..t.c...........................................................................................................

              391                                                                                                                            520
                                            TTCTAGATTCCCACTTTCGTGGGAATGACGG
1’-929603-    GTCATCGCCGTTTCGTCTTTGTCTAAAACCAAAACCGAAACCAACAACCCCAAAGGTATCGCCCATACTATCGAATACCTTAAAAAACACAAGGTCGAGACCTTTGCAAAATTCCCCAAAATCCCCTAAA
3’-1072218    -------------------------------------------------------------------------------------------AGGCGGAAACCTTTGCAAAAATCCCCAAAATCCCCTAAA
5-1591795-    -------------------------------------------------------------------------------------------ATCCCGAGACCTTTGCAAAATTCCCCAAAATCCCCTAAA
4’-1427636    -------------------------------------------------------------------------------------------ATCTAGAGACCTTTGCAAAATTCCCCAAAATCCCCTAAA
 Consensus    ...........................................................................................A....GAgACCTTTGCAAAAtTCCCCAAAATCCCCTAAA

              521                                                                                                                            650
1’-929603-    TTCCCACCAAGACATTTAGGGGATTTCTCATGAGCACCTTCTTTCAACAAACCGCCCAAGCCATGATTGCCAAACACATCGACCGCTTCCCGCTATTGAAGTTGGACCAGGTGATTGATTGGCAGCCGAT
3’-1072218    TTCCCACCAAGACATTTAGGGGATTTCTCATGAGCACCTTCTTCCGGCAAACCGCACAAGCCATAATTGCCAAACACATCGACCGTTTCCCGCTATTGAAGTTGGACCGGGTGATTGATTGGCAGCCGAT
5-1591795-    TTCCCACCAAGACATTTAGGGGATTTTCCATGAGCACCTTCTTTCAGCAAACCGCACAAGCCATGATTGCCAAACACATCGACCGTTTCCCACTATTGAAGTTGGATCAGGTAATTGATTGGCAACCGAT
4’-1427636    TTCCCACCAAGACATTTAGGGGATTTCTCAAGAGCACCTTCTTCCAGCAAACCGCCCAAGCCATGATTGCCAAACACATCGACCGCTTCCCGCTATTGAAGTTGGACCGGGTGATTGATTGGCAGCTGAT
 Consensus    TTCCCACCAAGACATTTAGGGGATTTctCAtGAGCACCTTCTT.CagCAAACCGC.CAAGCCATgATTGCCAAACACATCGACCG.TTCCCgCTATTGAAGTTGGAcC.GGTgATTGATTGGCAgCcGAT

              651                                                                                                                            780
1’-929603-    CGAACAATACCTGAACCGTCAAAAAACCCGTTACCTCCGAGACCACCGCGGTCGTCCCGCCTGTCCCCTGTTGTCCATGTTCAAAGCCGTCCTGCTCGGACAATGGCACAACCTCTCCGATCCCGAACTC
3’-1072218    CGAACCATACCTGAACCGTCAAA-AACCCGTTACCTCAGAGATTACCGCGGCCGTCCCGCCTATCCCCTGTTGTCCATGTTCAAAGCCGTCCTGCTCGGACAATGGCACAGCCTCTCCGATCCCGAACTC
5-1591795-    CGAACAGTACCTGAACCGTCAAAGAACCCGTTACCTTCGAGACCACCGCGGCCGTCCCGCCTATCCCCTGCTGTCCATGTTCAAAGCCGTCCTGCTCGGACAATGGCACAGCCTCTCCGATCCCGAACTC
```

```
4'-1427636  CGAACAATACCTGAACCGTCAAAAAACCCGTTACCTTAGAGACCACCGCGGCCGTCCTGCCTATCCCCTGCTGTCCATGTTCAAAGCCGTCCTGCTCGGACAATGGCACAGCCTCTCCGATCCCGAACTC
 Consensus  CGAACaaTACCTGAACCGTCAAA.AACCCGTTACCT..GAGAccACCGCGGcCGTCCcGCCTaTCCCCTG.TGTCCATGTTCAAAGCCGTCCTGCTCGGACAATGGCACAgCCTCTCCGATCCCGAACTC

            781                                                                                                                   910
1'-929603-  GAACACAGCCTCATCACCCGCATCGACTTCAACCTGTTTTGCCGTTTTGACGAACTGAGCATCCCCGATTACAGCACCTTATGCCGCTACCGCAACCGGCTGGCGCAAGACGACACCCTGTCCGAACTGT
3'-1072218  GAACACAGCCTCATCACCCGCATCGACTTCAACCTGTTTTGCCGTTTTGACGAACTGAGCATTCCCGATTACAGCACCTTATGCCGCTACCGCAACTGGTTGGCGCAAGACAACACCCTGTCCGAATTAT
5-1591795-  GAACACAGCCTCATCACCCGCATCGATTTCAACCTGTTTTGCCGTTTTGACGAACTGAGCATCCCCGATTACAGCACCTTATGCCGCTACCGCAACTGGCTGGCGCAAGACGACACCCTGTCCGAACTGT
4'-1427636  GAACACAGCCTCATTACCCGCATCGATTTCAACCTGTTTTGCCGTTTTGACGAACTGAGCATCCCCGATTACAGCACCTTATGCCGCTACCGCAACCGGCTGGCGCAAGACAATACCCTGTCTGAACTGT
 Consensus  GAACACAGCCTCATcACCCGCATCGA.TTCAACCTGTTTTGCCGTTTTGACGAACTGAGCATcCCCGATTACAGCACCTTATGCCGCTACCGCAAC.GGcTGGCGCAAGAC.AcACCCTGTCcGAAcTgT

            911                                                                                                                  1040
1'-929603-  TGGAACTGATTAACCGCCAACTGACCGAAAAAGGCTTAAAAGTAGAGAAAGCATCCGCCGCCGTCGTTGACGCCACCATTATTCAGACCGTCGGCAGCAAACAGCGCCAGGCCATAGAAGTCGATGAAGA
3'-1072218  TGGAACTGATCAACCGCCAACTGACCGAAAAAGGCTTAAAAGTAGAGAAAGCATCCGCCGCCGTCATTGATGCCACCATTATTCAGACCGCCGGCAGCACACAGCGTCAGGTCATAGAAGTCGATGAAGA
5-1591795-  TGGAACTGATTAACTGCCAACTGACCGAAAAAGGCTTAAAAGTAGAGAAAGCATCCGCCGCCGTCGTTGATGCCACCATTATTCAGACCGCTGGCAGCAAACAGCGTCAGGCCATAGAAGTCGATGAAGA
4'-1427636  TGGAACTGATTAACCGCCAACTGACCGAAAAAGGTTTAAAAATAGAGAAAGCATCCGCTGCCGTCGTTGACGCCACCATTATTCAGACCGCCGGCAGCAAACAGCGTCAGGCCATAGAAGTTGACGAAGA
 Consensus  TGGAACTGATtAACcGCCAACTGACCGAAAAAGGcTTAAAAgTAGAGAAAGCATCCGCcGCCGTCgTTGA.GCCACCATTATTCAGACCGccGGCAGCAaACAGCGtCAGGcCATAGAAGTcGAtGAAGA

            1041                                                                                                                 1170
1'-929603-  GGGACAAGTCAGCGGTCAAACCACACTGAGTAAGGACAAAAATGCCCGTTGGACAAAGAAAAACAGCCTCTACAAACTCGGTTACAAACAACATACACGTACCGATGCGGAAGGCTATATCGAGAAACTG
3'-1072218  AGGACAAATCAACGGCCAAACCACACCGAGTAAGGACAGCGATGCCCGTTGGATCAAGAAAAACGGCCTCTACAAACTCGGTTACAAACAACATACCCGTACCGATGCGGAAGGCTATATCGAGAAACTG
5-1591795-  AGGACAAGTCAGCGGCCAAACCACACCGAGTAAGGACAGCGATGCCCGTTGGATCAAGAAAAACGGCCTCTACAAACTCGGTTACAAACAACATACCCGTACCGATGCGGAAGGCTATATCGAGAAACTG
4'-1427636  AGGACAAATCAGCGGTCAAACCACACCGAGTAAGGACAGCGATGCCCGTTGGATAAAGAAAAACGGCCTCTACAAACTCGGTTACAAACAACATACCCGTACCGATGCAGAAGGCTATATCGAGAAACTG
 Consensus  aGGACAA.TCAgCGG.CAAACCACACcGAGTAAGGACAgcgATGCCCGTTGGAt.AAGAAAAACgGCCTCTACAAACTCGGTTACAAACAACATACcCGTACCGATGCgGAAGGCTATATCGAGAAACTG

            1171                                                                                                                 1300
1'-929603-  CACATTACTCCCGCCAATGCCCATGAGTGCAAACACCTGTCGCCGTTGTTGGAAGGGTTACCCGAAGGTACGACCATCTATGCCGACAAAGGCTACGACAGTGCGGAAAACCGGCAACATCTGGAAGAAC
3'-1072218  CACATTACCCCCGCCAATGCCCATGAGTGCAAACACCTGCCGCCTTTGTTGGAAGGACTGCCCAAAGGTACGACCGTCTATGCCGACAAAGGCTATGACAGTGCGGAAAACCGGCAACATCTGGAAGAAC
5-1591795-  CACATTACCCCCGCCAATGCCCATGAGTGCAAACACCTGTCGCCGTTGTTGGAAGGGTTACCCGAAGGTACGACCGTCTATGCCGACAAAGGCTATGACAGTGCGGAAAACCGGCAACATCTGGAAGAAC
4'-1427636  CACATTACCCCCGCCAATGCCCATGAGTGCAAACACCTGTCGCCGTTGTTGGAAGGACTGCCCAAAGGTACGACCGTCTATGCCGACAAAGGCTACGACAGTGCGGAAAACCGGCAACATCTGGAAGAAC
 Consensus  CACATTACcCCCGCCAATGCCCATGAGTGCAAACACCTGtCGCCgTTGTTGGAAGG..T.CCC.AAGGTACGACCgTCTATGCCGACAAAGGCTA.GACAGTGCGGAAAACCGGCAACATCTGGAAGAAC

            1301                                                                                                                 1430
1'-929603-  ATCAGTTGCAGGACGGCATTATGCGCAAAGCCTGCCGCAACCGTCCGCTGACGGAAACGCAAACCAAACGCAACCGGTATTTATCTAAGACCCGTTAT--------------------------------
3'-1072218  ATCAGTTGTTGGACGGCATTATGCGCAAAGCCTGCCGCAACCGTCCGCTGACGGAAACGCAAACCAAACGTAACCGATATTTATCGAAGACCCGTTATAGTGGATTAAATTTAAATCAGGACAAGGCGAC
5-1591795-  ATCAGTTGCAGGACGGCATTATGCGCAAAGCCTGCCGCAACCGCCCGCTGTCGGAAGTGCAAACCAAGCGTAACCGATATTTATCGAAGACCCGTTAT--------------------------------
4'-1427636  ATCAGTTGCTGGACGGCATTATGCGCAAAGCCCACCGCAACCGTCCGCTGACGGAAGTGCAAACCAAACGCAACCGATATTTGTCGAAGACCCGTTAT--------------------------------
 Consensus  ATCAGTTGc.GGACGGCATTATGCGCAAAGCCtgCCGCAACCGtCCGCTGaCGGAA..GCAAACCAAaCG.AACCGaTATTTaTCgAAGACCCGTTAT................................

            1431                                                                                                                 1560
1'-929603-  -------------------------------------------------------------------------GTGGTCGAACAAAGCTTCGGTACGCTGCACCGTAAATTCCGCTACGCCCGGGCGGCC
3'-1072218  GAAGCCGCAGACAGTACAAATAGTACGGCAAGGCGAGGCAACGCCGTACTGGTTTAAATTTAATCCACTATATGTGGTCGAACAGAGCTTCGGTACGCTGCACCGTAAATTCCGCTATGCGCGGGCAGCC
5-1591795-  -------------------------------------------------------------------------GTGGTCGAACAAAGCTTCGGTACGCTGCACCGTAAATTCCGCTACGCCCGGGCAGCC
4'-1427636  -------------------------------------------------------------------------GTGGTTGAACAGAGCTTCGGTACGCTGCACCGTAAATTCCGCTACGCCCGGGCAGCC
 Consensus  .........................................................................GTGGTcGAACA.AGCTTCGGTACGCTGCACCGTAAATTCCGCTAcGCcCGGGCaGCC
```

```
             1561                                                                                                                            1690
1’-929603-   TATTTCGGACTGATT--------------------------------------------------------------------TGCGCCCGCTGCCGCCTAAAAGGCAGCCCGGATGCCTGATTATCGGG
3’-1072218   TATTTCGGACTGATTAAAGTGAGTGCGCAAAGCCACCTGAAGGCGATGTGTTTGAACCTGTTGAAAGCGGCCAACAGGTTAAGTGCGCCCGCTGCCGCCTAAAAGGCAGCCCGGATGCCTGATTATCGGG
5-1591795-   TATTTCGGACTGATTAAAGTGAGTGTGCAAAGCCATCTGAAGGCGATGTGTTTGAACCTGTTGAAAGCCGCCAACAGGCTAAGTGCGCCTGTTGCCGCCTAAAAGGCAGCACGGATGCCTGATTATCGGG
4’-1427636   TATTTCGGACTGGTTAAAGTGAGTGCGCAAAGCCACCTGAAGGCGATGTGTTTGAACCTGTTGAAAGCCGCCAACAGGCTAAGTGCGCCCGCTGCCGCCTAAAAGGCAGCACGGATGCCTGATTATCGGG
 Consensus   TATTTCGGACTGaTTaaagtgagtg.gcaaagcca.ctgaaggcgatgtgtttgaacctgttgaaagc.gccaacagg.taagTGCGCCcGcTGCCGCCTAAAAGGCAGC.CGGATGCCTGATTATCGGG

             1691                                                                                                                            1820

1’-929603-   TATCCGGGGAGGATTAAGGGGGTATTTGGGTAAAATTAGGAGGTATTTGGGGCGAAAACAGCTGAAAACCTGTGTTTGGGTTTCGGCTGTCGGGAGGGAAAGGAATTTTGCAAAGGTCTCAAC-------
3’-1072218   TATCCGGGGAGGATTAAGGGGGTATTTGGGTAAAATTAGGCGGTATTTGGGGCGAAAACAGCCGAAAACCTGTGTTTGGGTTTCGGCTGTCGGGAGGGAAAGGAATTTTGCAAAGGTCTCAA--------
5-1591795-   TATCCGGGGAGGATTAAGGGGGCGTTTGGGTAGAATTAGGAGATATTTGGGGCGAAAACAGCCGAAAACCTGTGTTTGGGTTTCGGCTGTCGGGAGGGAAAGGAATTTTGCAAAGGTCTCATCCTGTTAT
4’-1427636   TATCCGGGGAGGATTAAGGGGATATTTGGGTAGAATTAGGCGGTATTTGGGGCGAAAACAGCCGAAAACCTGTGTTGGGGTTTCGGCTGTCGGG--GAAAAGGAATTTTGCAAAGGTCTCTT--------
 Consensus   TATCCGGGGAGGATTAAGGGGgtaTTTGGGTA.AATTAGG.GgTATTTGGGGCGAAAACAGCcGAAAACCTGTGTTtGGGTTTCGGCTGTCGGGagGgAAAGGAATTTTGCAAAGGTCTCa.........

             1821                                                                                                                            1950
1’-929603-   --------------------------------------TTGAACAAAAAAGAACCGCCCCGAATCAGGGC------------------------------------------------------------
3’-1072218   ----ACAAAAACAGAAACCTAAAGTCC-------------CGTCATTCCCGCGCAGGCGGGAATCCAGACC--------------------------------------------------CCCAACGCG
5-1591795-   TTTCACAAAAACAGAAAACCAAAAACAGCAACCTGAAATTCGTCATTCCCACGAAAGTGGGAATCCAGTGCGTTGAGTTTCAGCTATTTAGAATAAATTTTGAAACTCTAATCGCGTCATTCCCACGAAA
4’-1427636   --------------------------------------TTCGTCATTCCCGCGAAAGCGGGAATCTAGAATCT-------------------------------------------------CGGACTTT
 Consensus   ......................................ttcgtCAttcccgcg.a.gcggGAATC.aG......................................................c.......

             1951                                                                                                                            2080
1’-929603-   -------------------------------------------------------------------GGTTTTGCTTTGTGGCGGAAACGGTGG------------------------------------
3’-1072218   GCAGGAATC----------TATCGGAAATAACCGAAAC------------------CGGACGAACCTAGATTCCCGCTTTCGCGGGAATGACGGCAGGGTGGTTTCAGT----TGCTCCCGATAAATGCC
5-1591795-   GTGGGAATCCAGGACGCAAAATCTCAAGAAACCGTTTTACCCGATAAGTTTCCGCACCGACAACTCTAGATTCTCGCCTGCGCGGGAATGACGAATCCATCCATACGGAAACCTGCATCCCGTCATTCCC
4’-1427636   CAGATAATC-----------TTTGAATATTGCTGTTGTT-------------------CTAAGGTCTAGATTCCCGCTTTCGCGGGAATGACGGTTCAGTTGCTACGGT---------------------
 Consensus   .....aatc............t...a.....c.g...............................ctaGaTTc.CgctttcGCGGgAAtGacGg.....t...t.c.g......................

             2081                                                                                                                            2210
1’-929603-   -----------------GATTCGACTAAATT---------------------------------------------------------------------------------------------------
3’-1072218   GCCATCTCAAGTCTCGTCATTCCCTTAAAACAGAAAACCGAA----------------------------------------------------------------------------------------
5-1591795-   ACGAACCTGCATCCCGTCATTCCCACGAAAGTGGGAATCCAGTTTTTTGAGTTTCAGTCATTCCCGATAAATTGCCTTAGCATTGAATGTCTAGATTCCCGCCTGCGCGGGAATGACGGGATTTGAGATT
4’-1427636   --------TATTGTCAGGTTTCGGTTATGTTGGAATTTCG------------------------------------------------------------------------------------------
 Consensus   ...........t..c...aTTC...taaa...g.....c...........................................................................................

             2211                                                                                                                            2340
1’-929603-   ----------------------------------------------------------------------------------------------------------------------------------
3’-1072218   ---------ATCAGAAACCTAAAA--------TCCCGTCATTCCCGCGCAGGCGGGAATCTAGGTTT---------------------------------------------------------------
5-1591795-   GCGGCATTTATCAGGAGCAACAGAAGCCGCTCTGCCGTCATTCCCACGAAAGTGGGAATCCAGTTTTTTGAGTTTCAGTCATTCCCGATAAATTGCCTTAGCATTGAATGTCTAGATTCCCGCCTGCGCG
4’-1427636   ----------------------------------------------------------------------------------------------------------------------------------
 Consensus   ..................................................................................................................................
```

```
           2341                                                                                                                            2470
1'-929603- ----------------------------------------------------------------------------------------------------------------------------------
3'-1072218 --------------GTCGGCACGGAAACTT--------------------------------------------ATCGGGTAAAACGGTTTCTTTAGATT-----------------TTACGTT------
5-1591795- GGAATGACGAATCCATCCATACGGAAACCTGCACCACGTCATTCCCACGAACCTACATTCCGTCATTCCCACGAAAGTGGGAATCCAGTTTTTTGAGTTTCAGTCATTCCCGATAAATTGCCTTAGCATT
4'-1427636 ----------------------GGAAACTTA---------------------------------------------------------------------------------------------------
 Consensus ......................ggaaac.t....................................................................................................

           2471                                                                                                                            2600
1'-929603- -------TTATTTCATTGATTTAAATATAT-----------------------------------------------------------------------------------------------TTATT
3'-1072218 ------CTAGATTCCCGCCTGCGCGGGAATGACGA-------------------------------------------------------------------------------TGAAAAGATTGTTGTC
5-1591795- GAATGTCTAGATTCCCGCCTGCGCGGGAATGACGAATCCATCCGTACGGAAACCTGCATCCCGTCATTCCCACGAACCTACATTCCGTCATTCCCACGAAAGTGGGAATCCAGTTTTTTGAGTTTCAGTC
4'-1427636 -------TGAATTGAAACCTTTGCAAAAAT------------------------------------------------------------------------------------------------AGTC
 Consensus .......T..aTTc...ccT..gc...aAT.................................................................................................gTc

           2601                                                                                                                            2730
1'-929603- TCTTATGAAAATTTAATTTACCATAAAAA--------------CAGCC----------------------------------------------------------------------------------
3'-1072218 GCTTCGGATAAATTTTTGTCGCGTTGGGT-TCTAGATTCCCGCCTGCGCGGGAATGACGGCGGC--------------------------------------------------------GGGTTTCTGT
5-1591795- ATTTCCGATAAATTGCCTTAGCATTGAATGTCTAGATTCCCGCCTGCGCGGGAATGACGAATCCATCCGTACGAAAACCTGCACCACGTCATTCCCACGAAAGTGGGAATCCAGTTGCTTGAGTTTCAGT
4'-1427636 TGTTAACGAAATTTG---ACGCATAAAAA---------------TGCGCCAAAAAAT----------------------------------------------------------------------TTT
 Consensus ..TT..ga.AA.TT....t.gCaT..aa...............ctGCgc...aa...........................................................................t

           2731                                                                                                                            2860
1'-929603- ---------------------------------------------------------------------------------------------------------------------------ATATACA
3'-1072218 -TTTTCCGATAAATA-------------------------------------------------------------------------------CACACAAACTAAAATTTCGTCATTCCCATAAAAAAC
5-1591795- CATTTCCGATAAATTGCCTTAGCATTGAATGTCTAGATTCCCGCCTGCGCGGGAATGACGAATTCATCCGTACGGAAACCTGCACCACGTCATTCCCACGAACCTACATTCCGTCATTCCCACGAAAGTG
4'-1427636 CAATTGCCTAAAA-------------------------------------------------------------------------------------CCTTCCTAATATTGAGCA-------AAAAGTA
 Consensus ...tt.c...aaa.....................................................................................c...c..a...t....ca.......aaAa...

           2861                                                                                                                            2990
1'-929603- AAAATCTTGGAGTAACTATTGCATTACACTATTAGAAGAATGCCGTCTG---------------------------------------------AAGTGTTTTCGGACGGC-------------------
3'-1072218 AGAAAACCAAGTGA-GAATAACAATTCGTTGTAAACAAATAACTATTTGTTAATTTT----------------------------TATTAATATATGTAAAATCCCCCCCCCCCCGAAAGCT-------T
5-1591795- GGAATCCAGTGCGTTGAGTTTCAGTCATTTCCAATAAATTGCCTTAGTATTGAATGTCTGGATTCCCGCCTGCGCGGGAATGACGAATTCATCCGTACGGAAACCTGCATCCCGTCATTCCCACGAAAGT
4'-1427636 GGAAAAATCAGAAAAGTTTTGCATTTTGAAAATGAGATTGAGCATAAAATTT---------------------------------TAGTAACCTATGTTATTGCAAAGGTC---TCAATGCT-------A
 Consensus .gAA......g..a.g..Tt.CA.T....t...a..A.....C....t.tt...................................a.t.a...atgt.....C...c..C.....a...c.........

           2991                                                                                                                            3120
1'-929603- ---------ATTTCCCAAGCTGCCGGAAAAAGCTAAAATGCCC---------------------------------------------------------------------------------------
3'-1072218 AAGAATATAATTGTAAGCGTAACGATTATTTACGTTATGTTACCATA-----------------------------------------------------------------------------------
5-1591795- GGGAATCCAGTTTTTTGAGTTTCAGTCATTCCCGATAAATTGCCTTAGCATTGAATGTCTAGATTCCCGCCTGCGCGGGAATGACGGCGGAAATCTTGTTTATATTGAATCAAAAAAAACCTGCACCTTA
4'-1427636 AATATCTAGCAAAAAAGTGCGGTCAAAATTCACCGCACTTTTCGTTT-----------------------------------------------------------------------------------
 Consensus ...a......tt....g.G...c....Att.ac...A..Tt.Cc.t....................................................................................
```

```
              3121                                                          3182
1’-929603-    ---------GCAAACAGACAAGGAGCAGCGA
3’-1072218    ------TCCGACTACAATCCAAATTTTAGAG---------------------GTTTTAACTA
5-1591795-    ATCAGTTGGCGGTTTAGTCCGACTTTTGGGGTGCAGATCAAGCTTTCAGACGGTATTTCCTT
4’-1427636    ----------CGAACAATCGAAT
 Consensus    .............acA.tC.aa........................................
```

REP sequences are in green background
