# Supplementary material for: Bacterial repetitive extragenic palindromic sequences are DNA targets for Insertion Sequence elements
Source: BMC Genomics. 2006 Mar 24;7:62. doi: 10.1186/1471-2164-7-62 (PMC1525189; doi:10.1186/1471-2164-7-62)
Supplement: Additional File 12 — Copies of IS1106 in Neisseria meningitidis Z2491with type 2 association with REP sequences and their flanking regions. [file 1471-2164-7-62-S12.pdf]

REP sequences in flanking regions upstream IS1106 transposases in Neisseria meningitidis Z2491

```
             1                                                                                                                              130
 2'-727827   CCGTCATTCCCGCGCAGGCGGGAATCTAGTCT-GTTCGGTTTCAGTTATTTCCGATAAATGCCTGTTGCTTTTCATTTCTAGATTCCCACTTTCGTGGGAATGACGGGATTTTAGGTTTCTGATTTTGGT
3'-1063489   CCGTCATTCCCGCGCAGGCGGGAATCTAGTCT-GTTCGGTTTCAGTTATTTTCGATAAATGCCTGTTGCTTTTCATTTCTAGATTCACACTTTCGCGGGAATGACGA-ATTTTAGGTTTCTGATTTTGGT
 4-1179979   CCGTCATTCCCGCGAAAGCGGGAATCTAGGTTCGTCCGGTTTCGGTTATTTCCGATAGATTCCTGCCGCGTTGGGGGTCTGGATTCCCGCCTGCGCGGGAATGACGA-ATTTCAGGTTGCTGTTTTTGGT
 Consensus   CCGTCATTCCCGCGcAgGCGGGAATCTAGtcT.GTtCGGTTTCaGTTATTTcCGATAaATgCCTGttGCtTTtcattTCTaGATTCcCaCtTtCGcGGGAATGACGa.ATTTtAGGTTtCTGaTTTTGGT

             131                                                                                                                            260
 2'-727827   TTTCTGTCCTTGTGGGAATGACGGGATGTAG-GTTCGTAGGAATGA--------CGTGGTGCAGGTTTCCGTGCGGATGGATTCGTCATT-----------------------------------------
3'-1063489   TTTCTGTCCTTGTGGGAATGACGGGATATAG-GTTCGTAGGAATGA--------CGTGGTGCAGGTTTCCGTGCGGATGGATTCGTCATT-----CCCGCGCAGGCGGGAATCCAGACCTTAAGGCAGCG
 4-1179979   TTTCTGTTTTTGTGAAAATAACAGGATGAGATCTTTGCAAAATTCCTTTCCCTCCCGACAGCCGAAACCCAAACACAGGTTTTCGTCTATTTTCGCCCCAAATACCTCCTAATTTTACCCAAATACCCCC
 Consensus   TTTCTGTccTTGTGggAATgACgGGATgtag.gTTcGtAggAaTga........CgtggtGCaGgtttCCgtgCggAtGgaTTCGTCatT....ccc.......c....a.....acc..aa..c..c.

             261                                                                                                                            390
 2'-727827   ----------------------------------------------------------------------------------------------------------------------------------
3'-1063489   GCAATATTCAAAGATTATCTGAAAGTCTGAGATTCTAGATTCCCACTTTCGTGGGAATGACGGTTCA--------------GTTGCTACGGTTACTGTCAGGTTTCGGTTATGTTGGAATTTCGGGAAAC
 4-1179979   TTAACCCTCCCCGGATACCCGATAATCAGGCATCC--GGTCTCCTTTTAGGCGGCAGCGGGCGCACTTAGCTTGTTGGCGGCTTTCAACAGGTTCAAACACATCGCCTTCAGATGGCTTTGCGCACTCAC
 Consensus   ..aa...tc...g..ta.c.ga.a.tc.g..at.c..g.t..cc..tt..g.gg.a..g...g..c...............tt.c.ac.g.t.c...ca..t..c..t.a..t.g...t........ac

             391                                                                                                                            520
 2'-727827   ------------------------CCCGCGCAGGCGGGAATCTG--------------GAATTTCAATGCCTCAAGAATTTATCGGAAAAAACCAAAAC-------CCTTCCGTCATCATTCC--------
3'-1063489   TTAT--------GAATCGTCATTCCCGCGCAGGCGGGAATCTG--------------GAATTTCAATGCCTCAAGAATTTATCGGAAAAAACCAAAAC-------CCTTCCGTCATCATTCC--------
 4-1179979   TTTAATCAGTCCGAAATAGGCTGCCCGCGCATAGCGGAATTTACGGTGCAGCGTACCGAAGCTCTGTTCGACCACATATAGTGGATTAAATTTAAACCAGTACGGCGTTGCCTCGCCTTGCCGTACTATT
 Consensus   tt.........gaa......t.CCCGCGCAggcgGGAATcTg..............GAAttTCaaTgCctCaAgAatTtaTcGgaaAAAaccAAAaC.......CcTTcCgTCatCaTtCC........

             521                                                                                                                            650
 2'-727827   -----------CGCAAAAGCGGGAATCTAGAAATGAAAAGCAACAGGAATTTATCGGAAATGACCGAAA---CTGAACGGACTGGATTCCCGCTTTTGCGG-GAATGACGGCGACAGGGTT-GCTGTTAT
3'-1063489   -----------CGCAAAAGCGGGAATCTAGAAATGAAAAGCAACAGGAATTTATCGGAAATGACCGAAA---CTGAACGGACTGGATTCCCTCTTTTGCGG-GAATGACGGCGACAGGGTT-GCTGTTAT
 4-1179979   TGTACTGTCTGCGGCTTCGTCGCCTTGTCCTGATTTAAATTTAATCCACTATAACGGGTCTTCGACAAATACCGGTTGCGTTTGGTTTGCGTTTCCGACAGCGGACGGTTGCGGCAGGCTTTGCGCATAA
 Consensus   ...........CGcaaaaGcgGgaaTcTagaaATgaAAAgcaAcaggAaTtTAtCGGaaaTgaccgAAA...CtGaacgGacTGGaTTcCctcTtttgCgG.GaAtGacgGCGaCAGGgTT.GCtgtTAt

             651                                                                                                                            780
 2'-727827   AGTGGATGAACAAAAACCGGTACGGCGTTGTCTCGCCTTAGCTCGAAGAGAACGATTCTCTAAGGTGCTGAAGCACCAAGTGAATCGGTTC-CGTACTATCTGTACTGTCTGCGGCTTCGTCGTCTTGTC
3'-1063489   AGTGGATGAACAAAAACCGGTACGGCGTTGTCTCGCCTTAGTTCGAAGAGAACGATTCTCTAAGGTGCTGAAGCACCAAGTGAATCGGTTC-CGTACTATCTGTACTGTCTGCGGCTTCGTCGTCTTGTC
 4-1179979   TGCCGTCCAACAACTGATGTTCTTCCAGATGTTGCCGGTTTTCCGCACTGTCGTAGCCTTTGTCGGCATAGACGGTCGTACCTTTGGGCAGTCCTTCCAACAACGGCGGCAGGTGTTTGCACTCATGGGC
 Consensus   aGtgGatgAACAAaaaccGgTacggCgttgtcTcgCctTagttCGaAgaGaacgAttCTcTaagGtgcTgaAgcacCaagtgaaTcGGttc.CgTaCtAtCtgtactGtCtGcgGcTTcgtCgtcTtGtC

             781                                                                                                                            910
 2'-727827   CT--------GATTTTTGTTAATTCACTATATCGACATCGCCAAACGAAACTTCGTCATCGCCGTTTC-GTCTTTGTCTAAAACCAAAACCGAA----ACCAACAACCCCAAAGGTATCGCCCATACTAT
3'-1063489   CT--------GATTTTTGTTAATTCACTATATCGACATCGCCAAACGAAACTTCGTCATCGCCGTTTC-GTCTTTGTCTAAAACCAAAACCGAA----ACCAACAACCCCAAAGGTATCGCCCATACTAT
 4-1179979   ATTGGCGGGGGTAATGTGCAGTTTCTCGATATAGCCTTCCGCATCGGTACGGGTATGTTGTTTGTAACCGAGTTTGTAGAGATCCAACGGGCATCTTTGTCTTTACTCGGTGTGGTTTGGCCGCTGATTT
 Consensus   cT........GattTtTGttaaTTCaCtATATcGaCaTCgcCAaacGaAacttcgTcaTcgccGTttC.GtcTTTGTctAaAaCCAAaaccgAa....acCaacAacCccaaaGGTaTcGCCcaTacTaT
```

```
             911                                                                  Left End                                                  orf     1040
2'-727827    CGAATACCTTAAAAACACAAGGTC---------------------------GAGACCTTTGCAAAATTCCCCAAAATCCCCTAAATTCCCACCAAGCCATTTAGGGGATTTTCCATGAGCACCTTCTTCC
3'-1063489   CGAATACCTTAAAAACACAAGGTC---------------------------GAGACCTTTGCAAAATTCCCCAAAATCCCCCAA-TTCCCACCAAGACATTTAGGGGATTTCTCATGAGCACCTTCTTCC
4-1179979    GTCCTTCTTCGTCAACTTCTATGGCCTGGCGCTGTTTGCTGCCGGCGGTCTGAGACCTTTGCAAAATTCCCCAAAATCCCCTAAATTCCCACCAAGCCATTTAGGGGATTTTCCATGAGCACCTTCTTCC
Consensus    cgaaTaCcTtaaaAAacaCaAgGtC...........................GAGACCTTTGCAAAATTCCCCAAAATCCCCtAAaTTCCCACCAAGcCATTTAGGGGATTTtcCATGAGCACCTTCTTCC

             1041                                                          orf                                                          1170
2'-727827    GGCAAACCGCACAAGCCATGATTGCCAAACACATCGACCGTTTCCCGCTATTGAAGTTGGACCAGGTGATTGATTGGCAGCCGATCGAACAATACCTGAACCGTCAAAAAAACCGTTACCTTAGAGACCA
3'-1063489   GGCAAACCGCACAAGCCATGATTGCCAAACACATCGACCGCTTCCCACTATTGAAGTTGGACCAGGTGATTGATTGGCAACCAATCGAACAATACCTGAACCGTCAAAGAACCCGTTACCTTCGAGACCA
4-1179979    GGCAAACCGCACAAGCCATGATTGCCAAACACATCGACCGTTTCCCGCTATTGAAGTTGGACCAGGTGATTGATTGGCAGCCGATCGAACAATACCTGAACCGTCAAAAAAACCGTTACCTTAGAGACCA
Consensus    GGCAAACCGCACAAGCCATGATTGCCAAACACATCGACCGtTTCCCgCTATTGAAGTTGGACCAGGTGATTGATTGGCAgCCgATCGAACAATACCTGAACCGTCAAAaAAaCCGTTACCTTaGAGACCA

             1171                                                          orf                                                          1300
2'-727827    CCGCGGCCGTCCCGCCTACCCTCTGCTGTCCATGTTCAAAGCCGTCCTGCTCGGACAATGGCACAGCCTCTCCGATCCCGAACTCGAACACAGCCTCATCACCCGCATCGATTTCAACCTGTTTTGCCGT
3'-1063489   CCGCGGCCGTCCCGCCTACCCGCTGCTGTCCATGTTCAAAGCCGTCCTGCTCGGACAATGGCACAGCCTCTCCGATCCCGAACTCGAACACAGCCTCATCACCCGCATCGACTTCAACCTGTTTTGCCGT
4-1179979    CCGCGGCCGTCCCGCCTACCCTCTGCTGTCCATGTTCAAAGCCGTCCTGCTCGGACAATGGCACAGCCTCTCCGATCCCGAACTCGAACACAGCCTCATCACCCGCATCGATTTCAACCTGTTTTGCCGT
Consensus    CCGCGGCCGTCCCGCCTACCCtCTGCTGTCCATGTTCAAAGCCGTCCTGCTCGGACAATGGCACAGCCTCTCCGATCCCGAACTCGAACACAGCCTCATCACCCGCATCGAtTTCAACCTGTTTTGCCGT

             1301                                                          orf                                                          1430
2'-727827    TTCGACGAACTGATCATCCCCGATTACAGTACCTTATGCCGCTACCGCAACTGGCTGGCGCAAGACGACACCCTGTCCGAATTACTCAAACTGATTAACTGCCAACTGACCGAAAAAGGCCTAAAAATAG
3'-1063489   TTTGACGAACTGAGCATCCCCGATTACAGCACCTTATGCCGCTACCGCAACTGGCTGGCGCAAGACGACACCCTGTCCGAATTACTCAAACTGATTAACTGCCAACTGACCGAAAAAGGCCTAAAAATAG
4-1179979    TTCGACGAACTGATCATCCCCGATTACAGTACCTTATGCCGCTACCGCAACTGGCTGGCGCAAGACGACACCCTGTCCGAATTACTCAAACTGATTAACTGCCAACTGACCGAAAAAGGCCTAAAAATAG
Consensus    TTcGACGAACTGAtCATCCCCGATTACAGtACCTTATGCCGCTACCGCAACTGGCTGGCGCAAGACGACACCCTGTCCGAATTACTCAAACTGATTAACTGCCAACTGACCGAAAAAGGCCTAAAAATAG

             1431                                                          orf                                                          1560
2'-727827    AGAAAGCATCCGCCGCCGTCGTTGATGCCACCATTATCCAGACCGCCGGCAGCAAACAGCGTCAGGCCATAGAAGTTGACGAAGAAGGACAAATCAGCGGTCAAACCACACCGAGTAAGGACAGCGATGC
3'-1063489   AGAAAGCATCCGCCGCCGTCGTTGATGCCACCATTATCCAGACCGCCGGCAGCAAACAGCGTCAGGCCATAGAAGTTGACGAAGAAGGACAAATCAGCGGTCAAACCACACCGAGTAAGGACAGCGATGC
4-1179979    AGAAAGCATCCGCCGCCGTCGTTGATGCCACCATTATCCAGACCGCCGGCAGCAAACAGCGTCAGGCCATAGAAGTTGACGAAGAAGGACAAATCAGCGGTCAAACCACACCGAGTAAGGACAGCGATGC
Consensus    AGAAAGCATCCGCCGCCGTCGTTGATGCCACCATTATCCAGACCGCCGGCAGCAAACAGCGTCAGGCCATAGAAGTTGACGAAGAAGGACAAATCAGCGGTCAAACCACACCGAGTAAGGACAGCGATGC

             1561                                                          orf                                                          1690
2'-727827    CCGTTGGATCAAGAAAAACGGCCTCTACAAACTCGGTTACAAACAACATACCCGTACCGATGTGGAAGGCTATATCGAGAAACTGTACATCACTCCCACCAATGCCCATGAGTGCACACACCTGTCGCCG
3'-1063489   CCGTTGGATAAAGAAAAACGGCCTCTACAAACTCGGTTACAAACAACATACCCGTACCGATGCGGAAGGCTATATCGAGAAACTGTACATCACTCCCACCAATGCCCATGAGTGCACACACCTGTCGCCG
4-1179979    CCGTTGGATAAAGAAAAACGGCCTCTACAAACTCGGTTACAAACAACATACACGTACCGATGCGGAAGGCTATATCGAGAAACTGCACATTACTCCCGCCAATGCCCATGAGTGCAAACACCTGTCGCCG
Consensus    CCGTTGGATaAAGAAAAACGGCCTCTACAAACTCGGTTACAAACAACATACcCGTACCGATGcGGAAGGCTATATCGAGAAACTGtACATcACTCCCaCCAATGCCCATGAGTGCAcACACCTGTCGCCG

             1691                                                          orf                                                          1820
2'-727827    TTGTTGGAAGGACTGCCCAAAGGTACGACCGTCTATGCCGACAAAGGCTATGACAGTGCGGAAAACCGGCAACATCTGGAAGAACATCAGTTGCAGGACGGCATTATGCGCAAAGCCTGCCGCAACCGTC
3'-1063489   TTGTTGGAAGGACTGCCCAAAGGTACGACCGTCTATGCCGACAAAGGCTATGACAGTGCGGAAAACCGGCAACATCTGGAAGAACATCAGTTGCAGGACGGCATTATGCGCAAAGCCTGCCGCAACCGCC
4-1179979    TTGTTGGAAGGGTTACCCGAAGGTACGACCGTCTATGCCGACAAAGGCTACGACAGTGCGGAAAACCGGCAACATCTGGAAGAACATCAGTTGTTGGACGGCATTATGCGCAAAGCCTGCCGCAACCGCC
Consensus    TTGTTGGAAGGacTgCCCaAAGGTACGACCGTCTATGCCGACAAAGGCTAtGACAGTGCGGAAAACCGGCAACATCTGGAAGAACATCAGTTGcaGGACGGCATTATGCGCAAAGCCTGCCGCAACCGcC

             1821                                                          orf                                                          1950
```

```
2'-727827   CGCTGTCGGAAACGCAAACCAAACGCGACCGGTATTTGTCGAAGACCCGTGGCGATGTGTTTGAACCTGTTGAAAGCCGCCA----------------------ACAGGCTAAGTGCGCCCG------CT
3'-1063489  CGCTGTCGGAAGTGCAAACCAAACGCAACCGATATTTGTCGAAGACCCGTTATGTGGTTGAACAGAGCTTCGGTACGCTGCACCGTAAATTCCGCTACGCTCGGGCAGCCTATTTCGGACTGATTAAAGT
4-1179979   CGCTGTCGGAAGTGCAAACCAAACGCAACCGATATTTGTCGAAGACCCGTTATGTGGTTGAACAGAGCTTCGGTACGCTGCACCGTAAATTCCGCTACGCTCGGGCAGCCTATTTCGGACTGATTAAAGT
Consensus   CGCTGTCGGAAgtGCAAACCAAACGCaACCGaTATTTGTCGAAGACCCGTtatGtgGTtgaacAgagctTcGgtAcgCtgCAccgtaaattccgctacgctcgggCAGcCTAttTcgGaCtGattaaagT

            1951                            orf                       2032
2'-727827   GCCGCCCAAAGGCGACCGGATGCCTGATTATCGGGTATCTAGGGAG---------GATTAA
3'-1063489  GAGTGCGCAAAGCCACCTGAAAGCGATGTGTTTGAACCTGTTGAAAGCGGCCAACAGGTTAAGTGCGCCCGCTGCCGCCTAA
4-1179979   GAGTGCGCAAAGCCACCTGAAAGCGATGTGTTTGAACCTGTTGAAAGCGGCCAACAGGTTAAGTGCGCCCGCTGCCGCCTAA
Consensus   GagtgCgcAAAGcCacCtGaAaGCgatgTgtTtGaaccTgTtGaaAGcggccaacaGgTTAAgtgcgcccgctgccgcctaa
```

REP sequences in flanking regions downstream IS1106 transposases in *Neisseria meningitidis* Z2491

```
            1                                                    Rigth End                                                         130

1'-209578   AAAGGCGGCCCGGATGCCTGATTATCGGGTATCCGGGCAGGATTAAGGGGGGATTTGGGTAGAATTAGGAGGTATTTGG-GGCGAAAACAGCCGAAAACCTGTGTTGGGGTTTCGGTTGTTGGG-GGGAA
6-1673919   AAAGGCAGCCCGGATGCCTGATTATCGGGTATCCGGGGAGGATTAAGGGGGTATTTGGGTAGAATTAGGAGGTATTTGG-GGCGAAAACAGCCGAAAACCTGTGTTTGGGTTTCGGCTGTCGGGAGGGAA
5'-1550999  AAAGGCGGCCCGGATGCCTGATTATCGGGTATCCGGG---GATTAAGGGGGTATTTGGGTAGAATTAGGGAGTGATTGGTAGCGAAAACAGCTGAAAACCTGTGTTTGGGTTTCGGCTGTCGGGAGGGAA
Consensus   AAAGGCgGCCCGGATGCCTGATTATCGGGTATCCGGG.agGATTAAGGGGGtATTTGGGTAGAATTAGGagGTatTTGG.gGCGAAAACAGCcGAAAACCTGTGTTtGGGTTTCGGcTGTcGGGaGGGAA

            131        Rigth End                                                                                                   260

1'-209578   AGGAATTTTGCAAAGGTCTCCGTCGGGCATCTGCAGCCGTTATTCCCGCGCAGGCGGGAATCTAGTCTGTTCGGTTTCAGTTATTTCCGATAAATGCCTGTTGCTTTTCATTTCTAGATTCCCACTTTCG
6-1673919   GGGAATTTTGCAAAGGTCTCA-------------TCCTGTTATTTTCACAAAAACAGAAAAC-------------------------CAAAAACAGCAACCTGAAATTCGT-----CATTCCCGCGCAGG
5'-1550999  AGGAATGTTGCAAAGGTCTC------------------------------------------------------------------------------------TTTTCGT-----CATTCCCGC-----
Consensus   aGGAATtTTGCAAAGGTCTC................c.gttatt..c.c..a..c.g.aa.c.........................c.a.aa..gc....tg.ttTTCgT.....cATTCCCgC....g

            261                                                                                                                    390
1'-209578   TGGGAATGACGGTTCAGTTGCTACGGTTACTGTCAGGTTTCGGTTATGTTGGAATTTCGGGAAACTTATGAATCGTCATTCCCGCGCAGGCGGGAATCTAGACATTCAATGCTAAGGCAATTTATCGGGA
6-1673919   CGGGAATC------CAGTGCGTTGAGTTTCAG--------CTATTTAGAATAAATTTTGAAACTCTAATCG--CGTCATTCCCACGAAAGTTGGAATCCAGTTTCTTGAGTTTCAGTCATTCCCGATAAA
5'-1550999  --------------------------------------------------------------CACTTTT---CGTCATTCCCGCGAACGCGGGAATCTAGAATCTCGGACTTTCAGATAATCTTTGAAT
Consensus   .gggaat.......cagt...t...gtt.c.g........c..tt..g....aattt.g..acaCTtaT....CGTCATTCCCgCGaA.GcgGGAATCtAGa.tcTcga..tT.aggcaattc.t.gaaa

            391                                                                                                                    520
1'-209578   ATGACTGAAACTCAAAAAACTGGATTCCCACTTTCGTGGGAATGACGGGATTTTAGGTTTCTGATTTTGGTTTTCTGTTTTTGTAGGAATGATGAAATTTTGAGTTTTAGGAATTTACCGGAAA----AA
6-1673919   TTGCCTTAGCATTGAATGTCTAGATTCCCGCCTGCGCGGGAATGACGGCGGAGC-GGTTTCTGTTTTT----TCCGGTAAATACCCACAAGCTAAAATCCCGTTATTTTCACAAAAACAGAAAACCAAAA
5'-1550999  ATTGCTGTTGTTCTAAGGTCTAGATTCCCGCGTTCGCGGGAATGACGG------------------------TTCAGTTGCTACGGTTATTGTCAGGTTTTGGTTATGTTGGAATTTCGGGAAAC-----
Consensus   aTg.CTga...Tc.AA.gtCTaGATTCCCgC.TtCGcGGGAATGACGG.......ggtttctg.tttt....TtC.GTt..Tac.g..Atg.T.AaaTtttG.tttTtt.g.AattaC.GgAAAc...aa

            521                                                                                                                    650
1'-209578   ACAGAAACCGTTCTGTCGTCATTCCCGCGCAGGCGGGAATCTAGACATTCAATGCTAAGGCAATTTATCGGGAATGACTGAAACTCAAAAAACTGGATTCCCACTTTCGTGGGAATGACGGGATGTAGGT
```

```
6-1673919    ACAGCAACCTGAAATTCGTCATTCCCGCGCAGGCGGGAATCCAGTGTGTTGA-GTTTCAGCTATTTAGAATAAATTT-TGAAACTCTAATCGCGTCATTCCCACGAAAGTGGGAATCCAGTTTCTTGAGT
5'-1550999   ---------------TTATGAAT-------TGAGACCTTTGCAAAAATAGTCTGTTAACGAAATTTGACGCATAAAAATGCGCCAAAAAATTTTCAATTGCCTAAAACCTTCCTAA-------------
 Consensus   acag.aacc......TcgTcAtTcccgcgcaGgcgggaaTccAga.att..atGtTaa.GcaATTTa.cg.aaAt.a.TGaaaCtcaAAa..ct..ATTcCCac.aacgTgggaAt...g.....t..gt

             651                                                                                                                             780
1'-209578    TCGTGGGAATGACGGGATGTAGGTTTCCGTACGGATGGATTCGTCATTCCCGCGCAGGCGGGAATCTAGACCTTGGGATAACAGCAATATTCAAAGATTATCTGAAAGTTTGAGATTCTGGATTCCCACT
6-1673919    TTCAGTCATTTCCGATAAATTGCCTTAGCATTGAATG---TCTAGATTCCCGCCTGCGCGGGAATGACGGCGGAGCGGTTTCTGTTTTTTCCGGTAAATACCCACAAG---------CTAAAATCCTGTT
5'-1550999   ------------------------------------------------------------------------TATTGAGCAAAAAGTAGGAAAAATCAGAAA------------AGTTTTGCATT
 Consensus   t...g..a.t..cg..a..t.g..tt......g.atg...tc...attcccgc....gcgggaat...g.c...g.g.tttcaGcaatat.cag.aAatAtC.gaAAg.........ctagatTcccatT

             781                                                                                                                             910
1'-209578    TTCGTGGGAATGACGGGATTAGAGTTTCAAAATTTATTCTAAATAGCTGAAGCTCAACGCACTGGATTCCCGCCTGCGCGGGAATGACGAATTTTAGGTTTCTGATT--TTGTTTTTCTGTTTTTGTGGG
6-1673919    ATTTTCACAAAAACAGAAAACCAAAAACAGCAACCTG---AAATTCGTCATTCCCGCGCAGGCGGGAATCTGGTTCGTTCGGTTTCGCTTGTTTTAAGTTTCGGGTAACTTCCACTTCGTCATTCCCGCG
5'-1550999   TTGAAAATGAGATTGAGCATAAAATTTTAGTAACCTATGTTATTGCAAAGGTCTC-----------------------------------------------------------TTTTCGTCATTCCCGCC
 Consensus   tT..t.a.aA.aacgggaata.AatttcAg.Aacct.t.taAaT.c.t.a.tCtC.......gg....c.g..t.....gg..t..c...tttta.gtttc.g.t...tt..ttTTCgtcaTTcccGcg

             911                                                                                                                             1040
1'-209578    AATGATGAAATT-------TTGAGTTTTAGGAATTTATCGGAAAAAACAGAAACCGCTCTGCCGTCATTCCCG-CGCAGGCGGGAATCTAGACCTTAGAACAACAGTAATATTCAAAGATTATCTGAAAG
6-1673919    CAAGTGGGAATCCAGTGCGTTGAGTTTCAGCTATTTA-GAATAAATTTTGAAACTCTAATCGCGTCATTCCCA-CGAAAGTGGGAATCCAGTTTTTTGAGTTTCAGTCATTCCCGATAAATTGCCTTAGC
5'-1550999   ACTTTT----------------------------------------------------CGTCATTCCCCTCGAAAGCGGGAATCTAGAATCTCGGACTTTCAGATAATCTTTGAATATTGCTGTTG
 Consensus   aatgttg.aat........ttgagttt.ag..attta.....aaa....gaaac.....t..CGTCATTCCC..CGaAaGcGGGAATCtAGa.ttT.Gaacttcagtaatatcc.a.aAttttcct.a.g

             1041                                                                                                                            1170
1'-209578    TCCGGGATTCTGGATTCCCACTTTCGCGGGAATGACGAATTTTAGGTTTCTGATTTTGGTTTTCTGTTTTTGTGGGAATGATGAAATTTTGAGTTTTAGGAATTTATCGGAAAAAACAGAAACCGCTCCG
6-1673919    ATTGAATGTCTAGATTCTCGCCTGCGCGGGAATGACGAATCCATCCATACGGAAACCTGCATCCCGTCATTCC-------------------------------------------CACGAACCTGTATC
5'-1550999   TTCTAAGGTCTAGATTTCCGCGTTCGCGGGAATGACGGTTCAGTTGCTACGGTTATTGTCA-------------------------------------------------------GGTTTCGGTTATG
 Consensus   ttcgaa.gTCTaGATTccCgC.TtCGCGGGAATGACGaaTc..t.g.TaCgGatattggcat.c.gt..tt.........................................cag.aaCcg.Tatg

             1171                                                                                                                            1300
1'-209578    CCGTCATTCCCGCGCAGGCGGGAATCCAGACCTTAGAACAACAGTAATATTCAAAGATTATCTGAAAGTCCGGGATTCTGGATTCCCACTTTCGTGGGAATGACGGGATGTAGGTTTCCGTGCGGATGGA
6-1673919    CCGTCATTCCCACGAAAGTGGGAATCCAGCTTTTTGAGTTTCAGTCATTTCCGATAAATTGCCTTAGCATTGAATGTCTAGATTCCCGCCTGCGCGGGAATGACGGGATTTGAAATTGCGGC------AT
5'-1550999   TTGGAATTTCGGGAAACTTATGAATTGAGACCTTTGCAAAA----------------------ATAGTCTGTTAACGAAATTTGACGCATAAAAATGCGCCAAAAAATTTTCAATTGC-----------
 Consensus   ccGtcATTcCcgcgaA.gtggGAATccAGaccTTtGaa.aacagt.at.t.c.a..a.t..c..aaagtctG..a.tctagaTTccCgC.T.cg.ggGaatgAcgggATtT..aaTTgCg.........

             1301                                                                                                                            1430
1'-209578    TTCGTCATTCCCGCGCAGGCGGGAATCTAGACCTTAGAACAACAGCAATATTCAAAGATTATCTGAAAGTCCGGGATTCTGGATTCCCACTTTTGTGGGAATGACGGGATTTTAGGTTTCTGTTTTTGGT
6-1673919    TTATCGGGAGCAACAGAAGCCGCTCTGCCGTCATTCCCACGAAAGTGGGAATCCAGCTTTTTGAGT-----------------TTCAGTCATTTCCGATAAATTGCC----TTAGCATTGAATGTCTAGA
5'-1550999   -------------CTAAAACCTTCCTAATATTGAGCAAAAAGTAGGAAAAATCAGAAAAGTTTTGC-------------------------ATTTTGAAAATGAGA-----TTGAGCATAAAATTTTAGT
 Consensus   tt........c..C..AagCcg..cT...gtc.ttc.aAcaa.AG.aa.AaTCaaa.atttT.tG.................ttc...c.tTT.tGa.AAtgag......TTagg.tT.aattTtTaGt

             1431                                                                                                                            1560
1'-209578    TTTCTGTTTTTGTAGGAATGATGAAATTTTGAGTTTTAGGAATTTATCGGAAAAAACAGAAACCGCTCTGCCGTCATTCCCGCGCAGGCGGGAATCTAGACATTCAATTCTAAGGCAATTTATCGGGAAT
```

```
6-1673919   TTCCCGCCTGCGCGGGAATGACGGGATTT-GAGATTGCGGCATTTATCAGGAGCAACAGAAGCCGCTCTGCCGTCATTCCCACGAAAGTGGGAATCCAGTTTTTTGAGTTTCAGTCATTCCCGATAAATT
5’-1550999  AACCTATGTTATTGCAAAGGTCTCGAATT-------------------------------------------GTCATTCCCACGCAGGCGGGAATCTAGTCTGTTCGGTTTCAGTTATTTCCGATAAATT
 Consensus  ttcCtgt.Tt.gtgggAAtGacg.gAtTT.gag.tt..gg.atttatc.g.a..aacagaa.ccgctctgccGTCATTCCCaCGcAgGcGGGAATCtAGtcttTt.agTtTcAGtcAtTtccgataaAtT
```

```
            1561                                        1606
1’-209578   GACTGAAACTCAAAAAACTGGATTCCTACTTTCGTGGGAATGACGG
6-1673919   GCCTTAGCATTGAATGTCTGGATTCCCGCCTGCGCGGGAATGACGG
5’-1550999  CCTGCTGCTTTTTATTTCTAGATTCCCACTTTCGTGGGAATGACGA
 Consensus  gcct.agc.Tt.aAt.tCTgGATTCCcaCtTtCGtGGGAATGACGg
```

```
>7’-1683921
ACAAAAACCAGTACGGCGTTGCCTCGCCTTAGCTCAAAGAGAACGATTCTCTAAGGTGCTGAAGCACCAAGTGAATCGGTTCCGTACTATTTGTACTGTCTGCGGCTTTGCCGCCTTGTCCTGATTTTTGTTAATCCACTATATG
AATCGTCATTCCCGCGCAGGCGGGAATCCAGACATTCAATGCTAAGGCAATTTATCGGGAATGACTGAAACTCAAAAAGCTGGATTCCCACTTTCGTGGGAATGACGGGATTAGAGTTTCAAAATTTATTCTAAATAGCTGAAAC
TCAACGCACTGGATTCCCGCCTGCGCGGGAATGACGAATTTCAGGTTGCTGTTTTTGGTTTTCTATTTTTGTGAAAATAACGGGATTTCAGCTTGTGGGTATTTACCAGAAAAAACAGAAACCGCTCCGCCGTCATTCCCGCGCA
GGCGGGAATCTAGACATTCAATGCTAAGGCAATTTATCGGAAATGACTGAAACTCAAAAAACTGGATTCCCACTTTCGTGGGAATGACACGATTAGAGTTTCAAAATTTATTCTAAATAGCTGAAACTCAAAAAACTGGATTCCC
GCCTGCGCGGGAATGACGAAGTGGAAGTTACCCGAAATTTAAAACAAGCGAAACCGAACGAGCCGGATTCCCGCTTGCGTGGGAATGACGAATTTCAGGTTGCTGTTTTTGGTTTTCTGTTTTTGTGAAAATAACGGGATTTTAG
CTTGTGGGTATTTACCGGAAAAAACAGAAACCGCTCCGCCGTCATTCCCGCGCAGGCGGGAATCTAGACATTCAATGCTAAGGCAATTTATCGGAAATGACTGAAACTCAAAAAGCTGGATTCCCACTTTCGTGGGAATGACGAA
GTGGAAGTTACCCGAAACTTAAAACAAGCGAAACCGAACGAACTAGATTCCCACTTTCGTGGGAATGACGGCAGAGCGGCTTCTGTTGCTCCCGATAAATGCCGCAATCTCAAATCCCGTCATTCCCGCGCAGGCGGGAATCTAG
GTCTGTCAGTGCGGAAACTTATCAGGTAAAACGGTTTCTTGAGATTTTGCGTCCTGGATTCCCACT
```

The copy 7 is not aligned with the copies 1, 6 and 5 because is a remnant without the end of the IS.
REP sequences are in green background
