# Supplementary material for: Bacterial repetitive extragenic palindromic sequences are DNA targets for Insertion Sequence elements
Source: BMC Genomics. 2006 Mar 24;7:62. doi: 10.1186/1471-2164-7-62 (PMC1525189; doi:10.1186/1471-2164-7-62)
Supplement: Additional File 13 — IS1397 inserted into an E. coli REP element in E. coli CFT073 genome [file 1471-2164-7-62-S13.pdf]

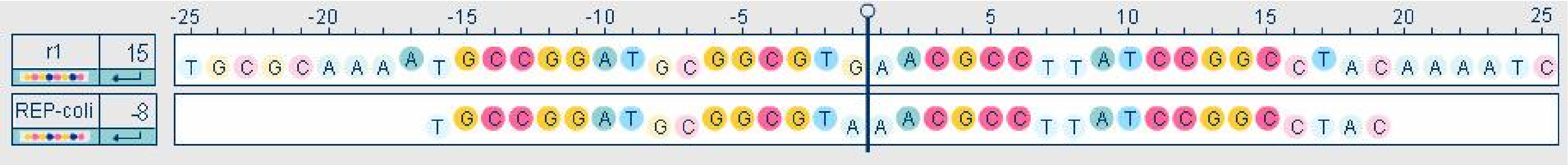

>1-Reconstructed

AAAAGCCAGCCTCGTGCGCAAAATGCCGGATGCGGCGTG|AACGCCTTATCCGGCCTACAAAATCGTGCTAATTCAAAA

>REP31 (E. coli)

TGCCGGATGCGGCGTA|AACGCCTTATCCGGCCTAC
