# Supplementary material for: Bacterial repetitive extragenic palindromic sequences are DNA targets for Insertion Sequence elements
Source: BMC Genomics. 2006 Mar 24;7:62. doi: 10.1186/1471-2164-7-62 (PMC1525189; doi:10.1186/1471-2164-7-62)
Supplement: Additional File 14 — Analyzed genomes [file 1471-2164-7-62-S14.pdf]

Pseudomonadaceae

Pseudomonas syringae

Pseudomonas syringae pv. tomato str. DC3000 (AE016853 NC_004578)

Pseudomonas aeruginosa

Pseudomonas aeruginosa PAO1 (AE004091 NC_002516)

Pseudomonas putida

Pseudomonas putida KT2440 (AE015451 NC_002947)

Enterobacteriaceae

Escherichia coli

Escherichia coli CFT073 (AE014075 NC_004431)

Escherichia coli K12 (U00096 NC_000913)

Escherichia coli O157:H7 (BA000007 NC_002695)

Escherichia coli O157:H7 EDL933 (AE005174 NC_002655)

Salmonella enterica

Salmonella enterica subsp. enterica serovar Typhi str. CT18 (AL513382 NC_003198)

Salmonella typhimurium

Salmonella typhimurium LT2 (AL513382 NC_003198)

Shigella flexneri

Shigella flexneri 2a str. 2457T (AE014073 NC_004741)

Shigella flexneri 2a str. 301 (AE005674 NC_004337)

Neisseriaceae

Neisseria meningitidis

Neisseria meningitidis MC58 (AE002098 NC_003112)

Neisseria meningitidis Z2491 (AL157959 NC_003116)

Rhizobiaceae

Agrobacterium tumefaciens

Agrobacterium tumefaciens str. C58 (AE008688 NC_003304)

Agrobacterium tumefaciens str. C58 (AE007869 NC_003062)

Sinorhizobium meliloti

Sinorhizobium meliloti 1021 (AL591688 NC_003047)

Deinococcaceae

Deinococcus radiodurans

Deinococcus radiodurans R1 (AE000513 NC_001263)

Rickettsiaceae

Rickettsia conorii

Rickettsia conorii str. Malish 7 (AE006914 NC_003103)

Mycobacteriaceae

Mycobacterium tuberculosis

Mycobacterium tuberculosis CDC1551 (AE000516 NC_002755)

We have selected all the available annotated genomes with reported presence of REP sequences. The 19 genomes with these features corresponding to 13 species of bacteria are showed above.
