# Supplementary material for: Bacterial repetitive extragenic palindromic sequences are DNA targets for Insertion Sequence elements
Source: BMC Genomics. 2006 Mar 24;7:62. doi: 10.1186/1471-2164-7-62 (PMC1525189; doi:10.1186/1471-2164-7-62)
Supplement: Additional File 15 — Canonical REP sequences corresponding to each analyzed species [file 1471-2164-7-62-S15.pdf]

Species	REP sequence
Pseudomonas syringae	GGACGCGGAGCGTCCAGAACGGCATGCCGACGCAGAGCGTCGCACGATAGT
Pseudomonas aeruginosa	GGTAGGGCGGATAACCGCTCGCGGTTATCCGCCG
Pseudomonas putida	CCGGCCTCTTCGCGGGTAAGCCCGCTCCTACAGGG
Escherichia coli	TGCCGGATGCGGCGTAAACGCCTTATCCGGCCTAC
Salmonella enterica typhi	TGCCGGATGGCGCTTCGCTTATCCGGCCTAC
Shigella flexneri	TGCCGGATGGCGCTTCGCTTATCCGGCCTAC
Salmonella typhimurium	TGCCGGATGGCGCTTCGCTTATCCGGCCTAC
Neisseria meningitidis	CCGTCATTCCCACGAAAGTGGGAATCTAGAA
Agrobacterium tumefaciens	ACGTCATCCTCGGGCTTGTCCCGAGGATCTGCAA
Sinorhizobium meliloti	ACCTTCTCCCCGCAAGCGGGGCGAAGG
Deinococcus radiodurans	GGGAGAGGGCCTTGCGAAGCAAGGGGTGAGGG
Rickettsia conorii	TATGTCATTCCCGGCAAAGCGGGAATCCAGT
Mycobacterium tuberculosis	GCGAGCAGACGCAAAATCGCCCATTTCGTACCCGAAATGGGCGATTTTGCGTCTGCTCGC
